# Janus‐Type Electrostatic Potential Gradient‐Activated Dynamic Zn^2+^‐Coordinating Nitrogen Sites in Molecularly Locked Nanocellulose Separators for Stable Zinc‐Ion Batteries

**DOI:** 10.1002/advs.75368

**Published:** 2026-04-29

**Authors:** Jie Liang, Shuxin Yang, Shuaiqi Zhao, Haitao Yang, Jingyuan Li, Jiaming Dong, Junwen Duan, Hao Yang, Yue Li, Yaxin Wang, Meilin Li, Ying Liu, Zhitao Shen, Rong Liu, Ruirui Cao, Fumin Li, Minshen Zhu, Yang Huang

**Affiliations:** ^1^ Henan Key Laboratory of Quantum Materials and Quantum Energy School of Quantum Information Future Technology Henan University Kaifeng China; ^2^ Advanced Materials Thrust The Hong Kong University of Science and Technology (Guangzhou) Guangzhou China; ^3^ Research Center For Materials Architectures and Integration of Nanomembranes (MAIN) Chemnitz University of Technology Chemnitz Germany

**Keywords:** azole‐terminated cellulose separators, dendrite‐free Zn anode, electrostatic potential gradient, ion transport regulation

## Abstract

Heterogeneous electric field distributions, uneven Zn^2+^ ion flux, and interfacial crosstalk typically trigger irreversible Zn redox reactions, accelerating the degradation of Zn‐metal energy storage systems. Herein, we present an interfacial stabilization strategy that addresses key challenges in Zn redox chemistry through the rational design of electrostatic potential gradients within cellulose‐based separators. The functionalization of cellulose nanofibrils (CNFs) with precisely arranged electron‐donating polyethyleneimine (PEI) and electron‐accepting benzimidazole moieties establishes a well‐defined bidirectional electron transfer network. PEI‐mediated electron donation to both the CNF matrix and benzimidazole ring stabilizes the surface charge environment via the interfacial dipole effect, while synergistic electron migration toward the π‐conjugated benzimidazole ring increases electron density at coordination‐active nitrogen sites, thereby enhancing Zn^2+^ binding affinity. The strengthened Zn‐N interactions lower the desolvation energy barrier, accelerate Zn^2+^ transport kinetics, and suppress parasitic interfacial reactions, collectively enabling homogeneous Zn deposition and improved interfacial stability. Consequently, the Zn||Zn symmetric cells exhibit exceptional reversibility over 600 h at 20 mA cm^−2^ and 20 mAh cm^−2^. When paired with MnO_2_ cathode, the full pouch cell retains 85.8% of its capacity after 4000 cycles at ∼10 C. This work highlights that molecular functionalization of separators enables next‐generation aqueous Zn batteries.

## Graphical abstract

A multifunctional benzimidazole‐terminated CNF separator is constructed via a PEI‐mediated covalent locking strategy, generating dense zincophilic sites that accelerate Zn^2+^ transport and desolvation while enabling uniform Zn (002) deposition and suppressing side reactions. This work offers strategic insights for the rational design of safe, durable, and high‐performance aqueous Zn‐ion batteries.

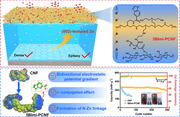

## 1 Introduction

Aqueous zinc‐ion batteries (ZIBs) are widely viewed as a sustainable successor to lithium‐ion technology for grid‐scale storage and next‐generation wearables, thanks to the earth abundance, low cost, and intrinsic safety [1, 2, 3]. Yet their commercial trajectory is stalled by rampant dendrite formation, interfacial corrosion, and hydrogen evolution, each of which reduces Coulombic efficiency and shortens cycle life [4, 5, 6, 7, 8, 9]. Efforts to date have concentrated almost exclusively on the anode/electrolyte interface via architected current collectors [10, 11, 12], protective coatings [13, 14, 15], electrolyte additives [16, 17, 18, 19, 20], and hydrogel matrices [21, 22, 23], while little attention has been given to the separator, a component positioned directly against that interface. Separators, functioning as both physical barriers and ionic pathways, are critical for ensuring interfacial stability and efficient ion transport in ZIBs [24, 25, 26]. Among currently available options, glass fiber (GF) separators are widely adopted owing to their superior electrolyte absorption capability and high ionic conductivity. However, conventional GF separators suffer from several limitations, including high cost, inherent brittleness, excessive thickness (200 µm), and inadequate resistance to Zn dendrite formation [27, 28]. Although the incorporation of inorganic or organic materials can improve mechanical strength and mitigate dendrite formation to some extent, these modifications still fail to achieve uniform Zn^2+^ ion distribution or stabilize the interfacial electric field, both of which are critical for stable Zn plating/stripping behavior [29, 30, 31, 32].

**FIGURE 1:**
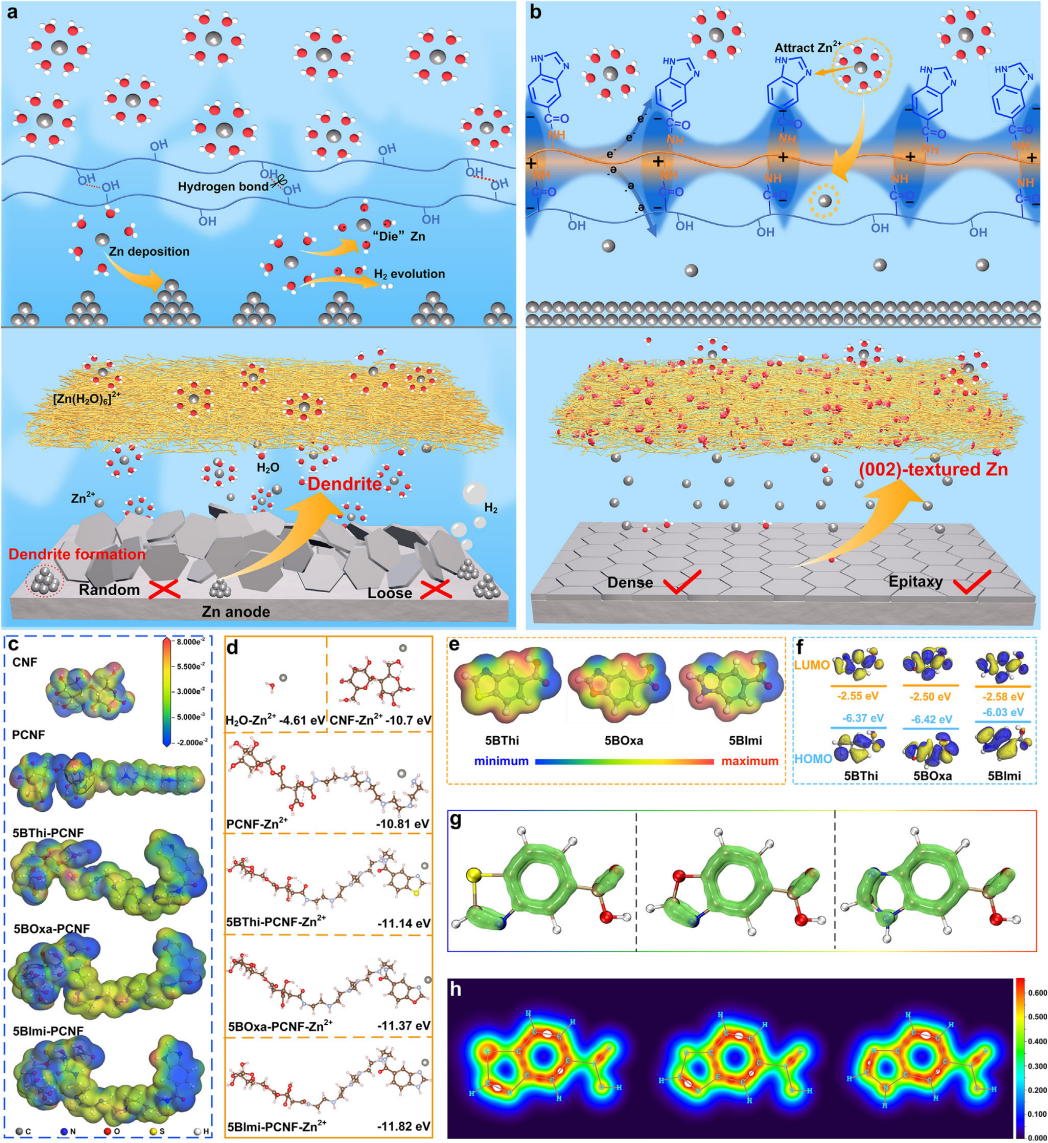
Molecular engineering of the functional CNF separators for controlled Zn deposition. Graphic illustrations of Zn plating mechanism on the bare Zn anodes with the (a) CNF and (b) 5BImi‐PCNF separators. (c) The calculated ESP distributions of CNF, PCNF, 5BThi‐PCNF, 5BOxa‐PCNF, and 5BImi‐PCNF separators. (d) The calculated binding energies of different functional separators to Zn^2+^ ion. (e) Molecular electrostatic potential images of 5BThi, 5BOxa, and 5BImi. (f) LUMO‐HOMO plots of 5BThi, 5BOxa, and 5BImi molecules. (g) π‐electron‐localization functional images of 5BThi, 5BOxa, and 5BImi. (h) π‐electron localized orbital locator profiles of 5BThi, 5BOxa, and 5BImi.

Cellulose nanofiber (CNF) separators, derived from the defibrillation of cellulosic biomass, have been recognized as a promising approach to address the longstanding dilemma between low separator thickness and excellent dendrite‐tolerance capability [33, 34]. The abundant hydroxyl groups present in CNFs not only enhance aqueous electrolyte uptake and interfacial wettability, but also reinforce the mechanical integrity of the separator by forming van der Waals interactions and intramolecular hydrogen bonds within the fibrous matrix, thereby allowing for a reduction in separator thickness [35, 36, 37]. Notwithstanding, the exposure of CNF to aqueous electrolytes readily triggers its internal rearrangement of the hydrogen‐bonding network, giving rise to swelling, structural deformation, and severe disruption of ion transport channels. This degradation reduces ion conductivity, which significantly impairs Zn deposition uniformity (Figure 1a), especially at high capacities and current densities, ultimately compromising cycling stability and areal capacity retention [25, 38].

The introduction of polar functional groups with strong zincophilicity onto CNF fibrils has been shown to improve structural stability and ion transport properties [39, 40]. The presence of these groups induces steric hindrance, which effectively suppresses hydrogen‐bond rearrangement within the nanofiber network and helps preserve the porous architecture under mechanical stress [41, 42, 43]. The strong Zn^2+^ affinity of the functional groups regulates Zn nucleation and steers preferential deposition along the thermodynamically favored (002) plane, which exhibits superior corrosion resistance owing to its lower surface energy and higher atomic density compared to the (100) and (101) planes [44, 45, 46]. However, despite these advancements, the random spatial distribution of zincophilic groups frequently often leads to electrostatic heterogeneity throughout the CNF framework. This heterogeneity causes localized charge accumulation and the formation of potential wells, which distort the overall electric field and hinder the selective adsorption of Zn^2+^ ions in the presence of competing anions. Consequently, both the uniformity of zinc deposition and long‐term electrochemical stability are adversely affected.

Herein, we present a spatially modulated electrostatic regulation strategy to optimize the electrostatic environment in CNF‐based separators, aimed at enhancing Zn anode stability. Through sequentially grafting electron‐donating polyethyleneimine (PEI) and electron‐accepting π‐conjugated azole derivatives (benzothiazole (5BThi), benzoxazole (5BOxa), and benzimidazole (5BImi)) onto the CNF surface, a Janus‐type architecture with an intrinsic molecular‐scale electrostatic potential gradient (CNF‐PEI‐azole) is developed. The amine‐rich PEI chains act as local electron reservoirs, donating electron density to the surface of CNFs through interfacial dipole effects, generating a uniform surface charge distribution along the CNF backbone. The excess electrons from PEI are partially transferred to adjacent electron‐deficient azole groups, forming an internal charge transfer equilibrium that minimizes local potential fluctuations and stabilizes the overall electrostatic environment. Meanwhile, the formed electron‐rich azole units act as moderate Lewis bases for Zn^2+^ coordination, with their π‐conjugated backbones and delocalized nitrogen lone pairs ensuring stable Zn─N binding. Among these, the 5BImi‐PCNF separator exhibits superior Zn─N coordinating ability due to its dual sp^2^‐hybridized nitrogen atoms in a conjugated π‐electron system, where enhanced charge delocalization creates stronger Zn^2+^ coordination sites. The reinforced Zn─N interactions effectively lower the Zn^2+^ diffusion energy barrier, regulate ionic flux, and homogenize the local electric field distribution, which collectively guide the epitaxial growth of Zn along the thermodynamically stable (002) plane (Figure 1b). Concurrently, it promotes the desolvation of Zn^2+^, which decreases the availability of water molecules at the deposition interface and minimizes water‐induced side reactions. Consequently, the Zn||Zn symmetric cell with the 5BImi‐PCNF separator can be stably operated for approximately 5500 h at 1 mA cm^−2^ and 1 mAh cm^−2^. Even under harsh conditions of 20 mA cm^−2^ and 20 mAh cm^−2^, the Zn||Zn symmetric cell with the 5BImi‐PCNF separator can still sustain an extended cycle period of over 600 h. When paired with a MnO_2_ cathode, the Zn||MnO_2_ full cell incorporating the 5BImi‐PCNF separator exhibits satisfactory rate performance and long cycling stability. This work provides a holistic perspective on the role of azole‐functionalized separators in promoting the application of metal batteries.

## 2 Results and Discussion

The spatial distribution of electron density within separators modulates the internal electric field, directly influencing Zn^2+^ ion transport kinetics in the electrolyte phase. To analyze these electron density variations in the 5BImi‐PCNF separator, the surface electrostatic potential (ESP) of functional CNF separators was calculated, where deep blue regions denote negative ESP domains and yellow–green regions correspond to positive ESP domains. As shown in Figure 1c, the pristine CNF displays intrinsic electrostatic heterogeneity, characterized by localized electron‐rich regions surrounding the hydrophilic ─OH groups, contrasted with electron‐deficient zones distributed along the carbon backbone. Upon PEI modification, the PCNF structure evolves into a dual‐polarity system, where the electronegative regions of CNF (arising from ─OH groups) coexist with electropositive regions contributed by the ─NH_2_ moieties of PEI. The formation of interfacial dipoles results from electron transfer from PEI's nitrogen lone pairs to the electron‐withdrawing sites of CNF, establishing an internal electrostatic gradient that enhances charge carrier interactions at the molecular interface. Subsequent azole incorporation further refines this electronic structure, producing a Janus‐type electrostatic potential landscape composed of CNF‐derived electronegative domains (left), PEI‐centered electropositive regions (middle), and azole‐derived electronegative domains (right). This hierarchical organization facilitates a bidirectional electron flow, wherein electrons migrate from PEI to both the CNF matrix and the azole rings. The resulting asymmetric electrostatic field establishes a directional potential gradient that serves as a driving force for ion transport across the CNF‐based separator. Moreover, the conjugated azole chains in 5BImi‐PCNF introduce extended electron delocalization, leading to a greater number of regions with strong electronegativity. This enhanced distribution of electron‐rich sites significantly improves the coordination interaction between azole rings and Zn^2+^ ions, thereby optimizing ion conduction channels and contributing to more uniform and efficient Zn^2+^ ion transport across the separator. Density functional theory (DFT) calculations were performed to elucidate the interaction mechanism and quantify adsorption energies of CNF‐based functionalized separators toward Zn^2+^ ion (Figure 1d). The binding energy of 5BImi‐PCNF to Zn^2+^ ion is determined to be −11.82 eV, which is significantly more negative than those of H_2_O (−4.61 eV), CNF (−10.7 eV), PCNF (−10.81 eV), 5BThi‐PCNF (−11.14 eV), and 5BOxa‐PCNF (−11.37 eV). This markedly stronger adsorption affinity indicates that 5BImi‐PCNF can offer abundant zincophilic sites for initial Zn nucleation and subsequent uniform Zn growth. To further elucidate the electronic origins of the enhanced zincophilicity in azole‐functionalized CNF separators, we conducted a detailed analysis of the electron density distribution of the three azole molecules using ESP mapping (Figure 1e). The results reveal that the negative electron density is primarily concentrated around the nitrogen atoms of the heterocyclic rings, with the exception of the regions near the carboxyl groups. However, 5BImi exhibits a significantly lower ESP value at the nitrogen site than 5BThi and 5BOxa, owing to the extended π‐conjugation between the imidazole ring and the benzene framework, which indicates its enhanced ability to attract Zn^2+^ ions. The molecular orbital energy levels of 5BThi, 5BOxa, and 5BImi were presented in Figure 1f. Compared to 5BThi and 5BOxa, 5BImi displays the lowest unoccupied molecular orbital (LUMO) energy level and the highest occupied molecular orbital (HOMO) energy level, reflecting superior electron‐accepting capability and a stronger propensity for Zn^2+^ adsorption, consistent with the adsorption energy results from DFT calculations. The influence of π‐conjugation properties on the electron‐accepting ability of azole‐functionalized CNF separators was investigated using the π‐electron localization function (ELF‐π) (Figure 1g), which provides insight into electron delocalization across the conjugated systems [47]. In 5BImi, the two sp^2^‐hybridized nitrogen atoms within the imidazole ring enable the most extensive electron delocalization, as indicated by a continuous electron cloud distribution spanning both the benzene ring and the imidazole moiety. This enhanced delocalization significantly improves its electron‐accepting capacity, thereby increasing the efficiency of electron transfer from PEI to 5BImi. In contrast, 5BThi and 5BOxa show less extensive π‐electron delocalization, with noticeable discontinuities in electron density around the sulfur and oxygen heteroatoms. These disruptions in the conjugated pathway arise from differences in electronegativity and orbital hybridization of sulfur and oxygen relative to nitrogen, which impair π‐conjugation and reduce their effectiveness in accepting electrons from donors such as PEI. Additionally, the π‐electron delocalization pathway was analyzed using the π‐electron‐localized orbital locator (π‐LOL) (Figure 1h) [47]. The red regions in the π‐LOL maps indicate areas of strong π‐electron delocalization, corresponding to efficient charge transport pathways within the conjugated systems. Among the azole derivatives, 5BImi displays the most uniform and intense red distribution across its molecular framework, indicating highly delocalized π‐electrons and well‐balanced charge density. The extensive π‐delocalization in 5BImi significantly enhances its electron‐accepting ability from PEI, thereby increasing electron cloud density at the imidazole nitrogen atoms. The elevated electron density strengthens the formation of robust and stable Zn─N coordination bonds via delocalized nitrogen lone pairs, promoting efficient Zn^2+^ transport and enhancing interfacial stability. In contrast, 5BThi and 5BOxa exhibit localized electron density around sulfur and oxygen atoms, respectively, suggesting weaker π‐conjugation. This restricted delocalization leads to diminished electron‐accepting capacity and reduced electron density at the coordination sites, weakening Zn─N interactions and impeding Zn^2+^ ion conduction. Thus, the enhanced electron delocalization in 5BImi plays a critical role in the formation of Janus‐type electrostatic potential gradients within the separator, contributing to better performance in Zn‐based electrochemical systems.

**FIGURE 2:**
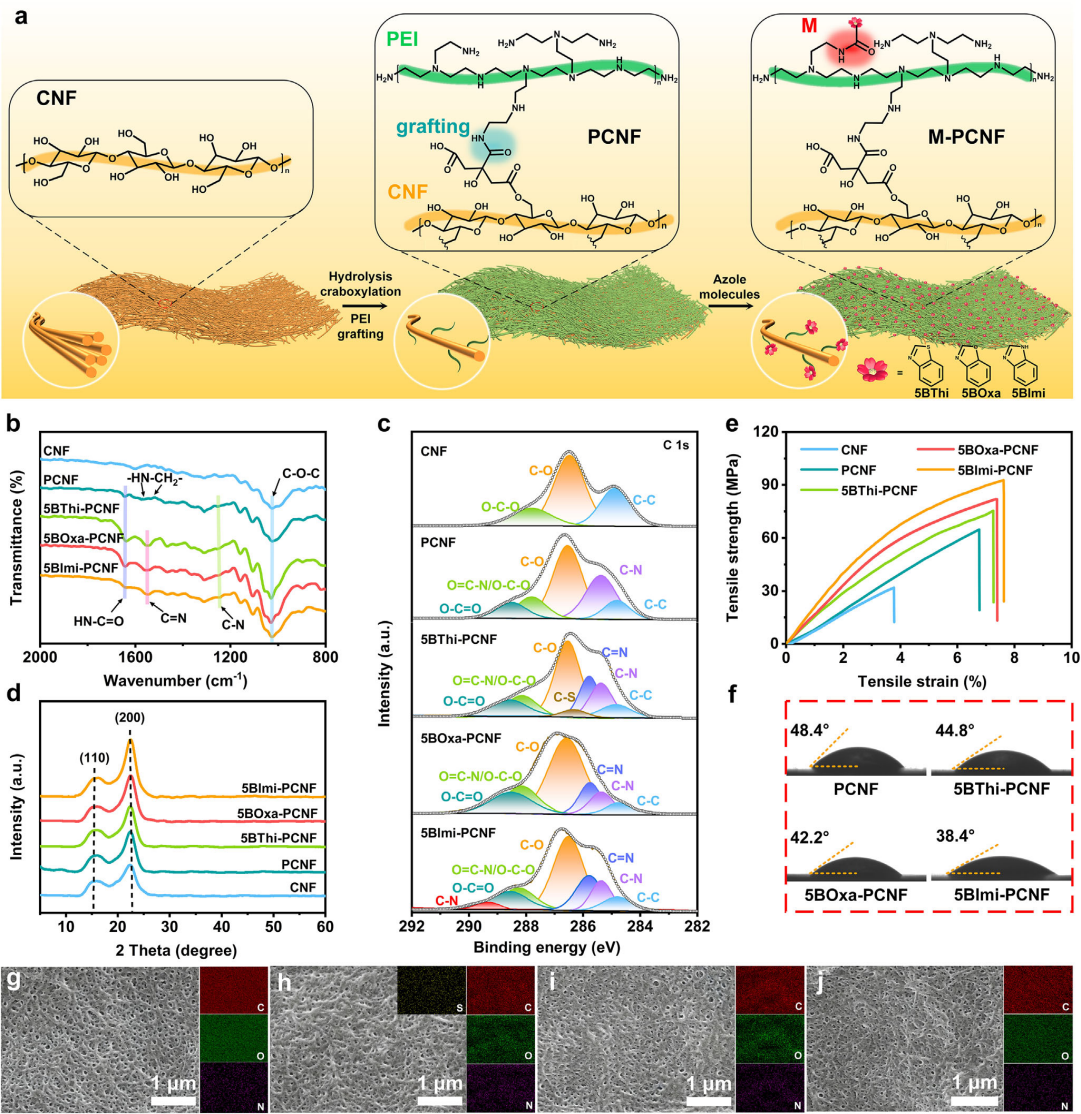
Preparation and characterization of the functional CNF separators. (a) Schematic illustration of the CNF, PCNF, 5BThi‐PCNF, 5BOxa‐PCNF, and 5BImi‐PCNF separators` fabrication. (b) FTIR spectrum, (c) high‐resolution C 1s XPS spectra, and (d) XRD patterns of the CNF, PCNF, 5BThi‐PCNF, 5BOxa‐PCNF, and 5BImi‐PCNF separators. (e) Tensile stress–strain curves of the CNF, PCNF, 5BThi‐PCNF, 5BOxa‐PCNF, and 5BImi‐PCNF separators. (f) Contact angle tests of the PCNF, 5BThi‐PCNF, 5BOxa‐PCNF, and 5BImi‐PCNF separators. SEM images and corresponding EDS mappings of the (g) PCNF, (h) 5BThi‐PCNF, (i) 5BOxa‐PCNF, and (j) 5BImi‐PCNF separators.

Cost‐effective, mass‐produced bleached pulp was selected as the starting material for the fabrication of CNF, PCNF, and M‐PCNF (Figure 2a). To enable efficient surface chemical modification, the pulp was initially treated with a bespoke hydrated multi‐carboxylic acid deep eutectic solvent (H‐DES), which disrupted the hydrogen bonding network within the cellulose chains and introduced reactive carboxyl groups along the polymer backbone [48]. The resulting carboxylated cellulose nanofibers (C‐CNF) were then cross‐linked with polyethyleneimine (PEI), a branched polyamine rich in amino functionalities, through a combination of electrostatic interactions and covalent amide bond formation [49]. This process produces a stable, cross‐linked PCNF network with enhanced mechanical properties and continuous PEI coverage over the accessible CNF surface. Subsequently, individual azole compounds were introduced and covalently anchored onto the PCNF surface via a carbodimide‐mediated amidation reaction, where the carboxyl group of the azole reacts with the primary amine group of the PEI to form stable amide linkages. This surface functionalization endows the nanocellulose matrix with enhanced chemical functionality and tunable reactivity for potential advanced applications. Finally, the free‐standing M‐PCNF separators, designed as 5BThi‐PCNF, 5BOxa‐PCNF, and 5BImi‐PCNF, were fabricated via vacuum filtration (Figure S1) [50]. The chemical properties of CNF, PCNF, and M‐PCNF separators were thoroughly examined using Fourier transform infrared (FTIR), X‐ray photoelectron spectroscopy (XPS), and X‐ray diffraction (XRD). Specifically, compared to the CNF sample, the FTIR spectra of PCNF display three new absorption peaks at 1637, 1563, and 1242 cm^−1^, corresponding to the stretching vibration of O═C─NH, the bending vibration of N─H and the stretching vibration of C─N, respectively, signifying the successful cross‐linking of PEI with the CNF structure (Figure 2b; Figure S2) [49, 51, 52]. After the introduction of 5BThi, 5BOxa, or 5BImi molecules, the stretching vibrations of O═C─NH become more pronounced and shift to 1646 cm^−1^. Additionally, a new absorption peak at 1542 cm^−1^ emerges, which is attributed to the C═N stretching vibration from the azole rings [53]. This further validates the efficient grafting of heterocyclic azole compounds onto the PCNF framework. Concurrently, XPS analysis corroborates these structural changes and the shift in the chemical state of C/N elements (Figure 2c). Notably, the presence of two distinct peaks at 287.8 eV (O═C─N) and 285.4 eV (C─N) in PCNF, which are absent in CNF, indicates the successful incorporation of PEI into the PCNF structure [49]. Upon azole functionalization, the modified variants (5BThi‐PCNF, 5BOxa‐PCNF, and 5BImi‐PCNF) display a noticeable shift of O═C─N to 288.2 eV, along with the emergence of C═N bond (285.8 eV). In particular, beyond the characteristic N absorption peak attributable to PCNF, the high‐resolution deconvolution of the N 1s spectrum in 5BImi‐PCNF reveals two distinct N bonding states, namely C─N─C (400.1 eV) and C═N (398.7 eV), while 5BThi‐PCNF and 5BOxa‐PCNF contain only C═N, located at 399.2 and 399.7 eV, respectively (Figure S3) [53, 54]. The S signal observed in the full XPS spectrum, coupled with the clear identification of spin‐orbital peaks at 165.2 eV (2p_1/2_) and 163.9 eV (2p_3/2_) in the S 2p spectrum, indicates the successful formation of 5BThi‐PCNF. These findings firmly support the covalent integration of imidazole, oxazole, and thiazole moieties into PCNF [53].

Furthermore, XRD analysis of the functionalized separators display identical characteristic diffraction signatures at 16.2 and 22.6°, indexed to the (110) and (200) crystallographic planes of CNF, respectively [48]. These observations substantiate that the structural integrity of CNF crystalline framework remains intact upon covalent attachment of PEI and heterocyclic azole functionalities (Figure 2d). The tensile strength of the various separators was evaluated, as shown in Figure 2e. Among them, the 5BImi‐PCNF separator demonstrates the highest strength at 92.7 MPa, significantly surpassing that of CNF (31.9 MPa), as well as PCNF (64.7 MPa), 5BThi‐PCNF (75.5 MPa), and 5BOxa‐PCNF (82.0 MPa). Notably, even after infiltration with aqueous electrolyte, the 5BImi‐PCNF separator maintains a high tensile strength of 78.2 MPa (Figure S4). In contrast, the mechanical properties of the other separators rapidly degrade in the wet state, especially for the CNF separator, whose strength decreases to only 23.9 MPa. This superior wetting mechanical stability is mainly attributed to the covalent PEI‐azole framework in 5BImi‐PCNF, which provides greater resistance to water‐induced softening than the hydrogen‐bonded network in pristine CNF. Coupled with its efficient electrolyte uptake and robust retention, this structural integrity collectively stabilizes the electrode‐electrolyte interface, thereby enabling sustained electrochemical cycling performance (Figure 2f; Figures S5 and S6) [55]. To decouple the influence of H‐DES preprocessing and subsequent molecular functionalization on the structural evolution and porosity, we systematically compared the pristine CNF, C‐CNF, and functionalized separators using SEM and pore structure analysis. The H‐DES treatment subtly refines the CNF scaffold, reducing the average fiber diameter from 19.8 to 17.7 nm and slightly adjusting the pore size (from ∼74 to ∼77 nm), while maintaining the integrity of the porous framework. In stark contrast, the subsequent grafting of azole moieties (5BThi, 5BOxa, and 5BImi) induces a pronounced pore refinement, narrowing the average diameter to below 60 nm while strictly preserving the nanofibrous morphology (Figure 2g–j; Figures S7–S9). This structural density, though contributing to the suppression of Zn dendrites, is a direct consequence of the steric hindrance and enhanced intermolecular interactions introduced by the functional group. Furthermore, azole molecular grafting results in only a marginal thickness increase from 37 to ∼40 µm, leaving the separators significantly more compact than commercial glass fiber (GF) separators (Figure S10). This suggests that the grafting process induces minimal structural expansion, allowing the functional CNF separators to retain their inherent compactness and high density.

**FIGURE 3:**
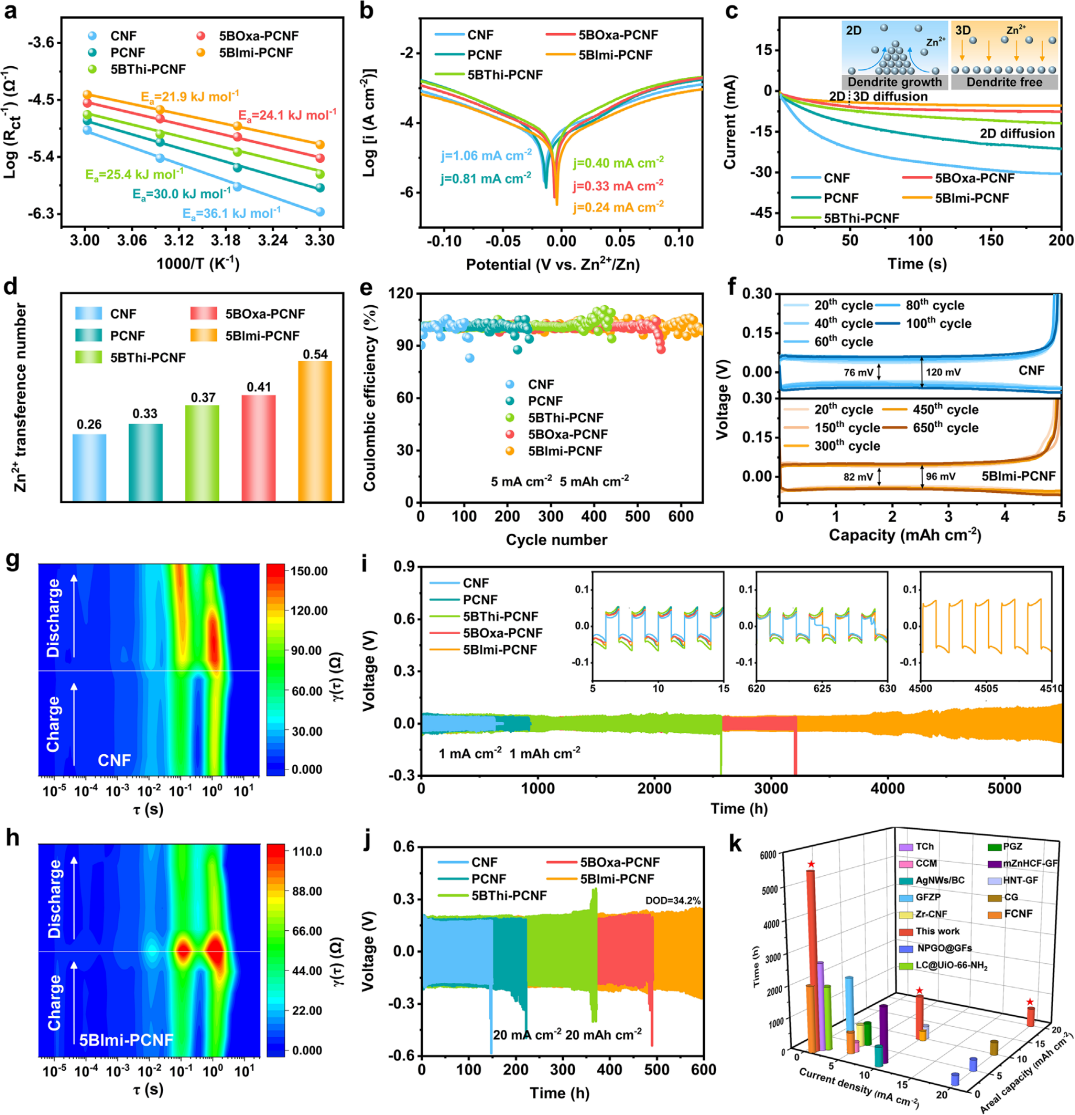
Electrochemical performance of the Zn||Cu cells and Zn||Zn cells with functionalized CNF separators. (a) Arrhenius curves and calculated E_a_ values with the use of CNF, PCNF, 5BThi‐PCNF, 5BOxa‐PCNF, and 5BImi‐PCNF separators. (b) Tafel curves of the Zn||Zn symmetric cells with CNF, PCNF, 5BThi‐PCNF, 5BOxa‐PCNF, and 5BImi‐PCNF separators. (c) Chronoamperometry test of Zn electrode with CNF, PCNF, 5BThi‐PCNF, 5BOxa‐PCNF, and 5BImi‐PCNF separators. (d) Zn^2+^ transference numbers of the Zn||Zn symmetric cells with CNF, PCNF, 5BThi‐PCNF, 5BOxa‐PCNF, and 5BImi‐PCNF separators. (e) CE measurements of the Zn||Cu cells with the CNF, PCNF, 5BThi‐PCNF, 5BOxa‐PCNF, and 5BImi‐PCNF separators at 5 mA cm^−2^ and 5 mAh cm^−2^, and (f) corresponding voltage profiles at various cycles. DRT plots for the charge and discharge processes of Zn||Zn cells with (g) CNF and (h) 5BImi‐CNF separators. Long‐term cycling performances of the Zn||Zn cells with the CNF, PCNF, 5BThi‐PCNF, 5BOxa‐PCNF, and 5BImi‐PCNF separators at (i) 1 mA cm^−2^ and 1 mAh cm^−2^ and (j) 20 mA cm^−2^ and 20 mAh cm^−2^. (k) Comparison analysis of cyclic reversibility with recent studies, showing current density, areal capacity, and cycle lifespan.

To investigate the interfacial stability effects imparted by functional CNF separators, systematic electrochemical studies were conducted. Specifically, temperature‐dependent electrochemical impedance spectroscopy (EIS) tests were performed on Zn||Zn cells at temperatures ranging from 30°C to 60°C to determine the desolvation activation energy (E_a_) (Figure 3a; Figure S11). The E_a_ associated with the 5BImi‐PCNF separator is calculated to be 21.9 kJ mol^−1^, lower than that of CNF (36.1 kJ mol^−1^), PCNF (30.0 kJ mol^−1^), 5BThi‐PCNF (25.4 kJ mol^−1^), and 5BOxa‐PCNF (24.1 kJ mol^−1^). This significant reduction of the energy barrier originates from the abundant nitrogen sites within the imidazole moieties, which effectively weaken the Zn─O coordination in the hydrated clusters, accelerating the desolvation process. Consequently, these enhanced kinetics not only facilitate Zn deposition but also suppress water‐induced side reactions by reducing the interfacial availability of active water molecules [56]. To further evaluate the correlation between the deposition quality and side reaction suppression across different separators, the corrosion behavior was systematically investigated. As further depicted in Figure 3b, the corrosion current density of the cell with the 5BImi‐PCNF separator (0.24 mA cm^−2^) is lower compared to that with CNF (1.06 mA cm^−2^), PCNF (0.81 mA cm^−2^), 5BThi‐PCNF (0.40 mA cm^−2^), and 5BOxa‐PCNF (0.33 mA cm^−2^), suggesting better corrosion resistance. This contributes to the excellent electrochemical stability windows of 5BImi‐PCNF separator (Figure S12). Moreover, the Zn growth mechanism with different separators was analyzed by chronoamperometry (CA) (Figure 3c). The current density of cell with the CNF separator continuously rises within 200 s, signifying an uncontrolled and rapid 2D diffusion process. As for the cell with 5BImi‐PCNF separator, there is a comparatively restricted 2D diffusion followed by a sustained 3D current diffusion in the subsequent stage, along with a lower steady‐state current. This clearly demonstrates that the 5BImi‐PCNF separator effectively suppresses nucleation overgrowth, facilitates uniform Zn deposition, and mitigates the tip effects. The Zn^2+^ ion transference number (t_Zn_^2+^), determined from the CA curves and corresponding Nyquist plots of Zn||Zn cells with different separators, further supports this conclusion [57]. As presented in Figure 3d and Figure S13, the t_Zn_^2+^ with 5BImi‐PCNF separator (0.54) system considerably exceeds that obtained with CNF (0.26), PCNF (0.33), 5BThi‐PCNF (0.37), and 5BOxa‐PCNF (0.41) separators. This trend is further validated by the thickness‐controlled comparison illustrated in Figure S14, where 5BImi‐PCNF exhibits lower impedance and a higher Zn^2+^ transference number than CNF at a matched thickness of 40 µm. These results indicate that the enhanced Zn^2+^ transport primarily originates from chemical functionalization rather than thickness variation. Additionally, Zn||Cu asymmetric cells incorporating CNF, PCNF, 5BThi‐PCNF, 5BOxa‐PCNF, and 5BImi‐PCNF separators were also subjected to Coulombic efficiency (CE) measurements, aiming to elucidate their influence on the reversibility of the Zn anode [58]. As illustrated in Figure 3e, the initial CE with the 5BImi‐PCNF separator reaches 98.3%, after which the CE rapidly stabilizes at 99.9% from the 5^th^ to the 650^th^ cycle. In sharp contrast, the use of the pristine CNF separator leads to a considerably lower initial CE of only 90.4%, with a short circuit failure occurring before 113 cycles. Notably, the life span of the Zn||Cu cell with 5BImi‐PCNF separator (1300 h) is longer than that of the Zn||Cu cells with the PCNF, 5BThi‐PCNF, and 5BOxa‐PCNF separators. While the larger pores of CNF separator initially provide a mass‐transport advantage that lowers the voltage gap (76 mV), this transient benefit is rapidly negated by its high intrinsic desolvation barrier and unregulated Zn deposition. Conversely, 5BImi‐PCNF fundamentally optimizes interfacial kinetics via nitrogen‐rich sites that lower the activation energy for Zn^2+^ transfer, ensuring a homogenized flux and superior long‐term reversibility (Figure 3f; Figures S15 and S16) [59].

To evaluate the charge transfer process of the Zn||Zn symmetric cells using different functional CNF separators, in situ EIS measurements were conducted to analyze the impedance variations of Zn||Zn cells during the third cycle. The resulting Nyquist plots were subsequently transformed into relaxation time distribution (DRT) to gain a more comprehensive understanding of the interfacial kinetic processes, as shown in Figure 3g,h and Figure S17. The signals observed within the time range of 10^−3^ to 1 s are primarily assigned to the charge transfer impedance at the electrodes interface (R_ct_), while those exceeding 1 s are closely associated with the diffusion impedance of Zn^2+^ ions within the Zn deposition electrode [60]. Notably, the use of the 5BImi‐PCNF separator not only significantly leads to a substantial reduction in both charge transfer and diffusion resistance, but also enhances the reversibility of Zn stripping/plating, in comparison to cells utilizing the CNF separator. To further validate these interfacial effects, an assessment of the cycling stability of the Zn||Zn symmetric cells with different functional CNF separators was systematically carried out. Under the condition of 1 mA cm^−2^ and 1 mAh cm^−2^, the Zn||Zn cell incorporating 5BImi‐PCNF separator can offer stable operation for over 5500 h, while the Zn||Zn cells with the CNF, PCNF, 5BThi‐PCNF, and 5BOxa‐PCNF separators exhibit significant voltage fluctuations after 628, 920, 2569, and 3200 h, respectively (Figure 3i). To decouple the influence of the monolithic architecture from the chemical composition, a laminated control separator (CNF‐PEI‐5BImi) was fabricated by physically stacking discrete layers. This laminated counterpart fails prematurely after only ∼458 h with significantly increased polarization (Figure S18). This dramatic performance disparity confirms that the efficacy of 5BImi‐PCNF stems from its continuous, molecular‐scale electrostatic gradient, as evidenced by the systematic shifts in surface and Zeta potential (Figures S19 and S20), rather than a mere additive effect of its components. This result suggests the critical role of structural integration in modulating the local ion flux and providing a regulated interfacial environment for uniform Zn^2+^ deposition. Benefiting from its rapid interfacial reactive kinetics, the 5BImi‐PCNF separator delivers improved cycling stability, particularly under high current density conditions, as evidenced in Figure 3j and Figures S21 and S22. When the current density and areal capacity are increased to 10 mA cm^−2^ and 10 mAh cm^−2^, the Zn||Zn cell still works stably for 1400 h with 5BImi‐PCNF separator (Figure S22). However, the Zn||Zn cell with the CNF separator rapidly fails after only 375 h, and its performance is inferior to that of the Zn||Zn cells with the PCNF, 5BThi‐PCNF, and 5BOxa‐PCNF separators. Notably, the 5BImi‐PCNF separator even enables stable Zn plating/stripping operation for up to 600 h when operated at a high current density of 20 mA cm^−2^ and a capacity of 20 mAh cm^−2^ (Figure 3j; Figure S23), further confirming its exceptional electrochemical reversibility. Figure S24 illustrates the rate performance of the Zn||Zn symmetric cells based on different functional CNF separators across a wide current density range from 1 to 20 mA cm^−2^. The Zn||Zn cell with the 5BImi‐PCNF separator delivers stable cycling over the entire range of current densities, accompanied by minimum voltage hysteresis. Nevertheless, the Zn||Zn cells with the CNF, PCNF, 5BThi‐PCNF, and 5BOxa‐PCNF separators experience a gradual increase in voltage hysteresis, particularly at a current density of 10 mA cm^−2^ and an areal capacity of 10 mAh cm^−2^. The superior performance of 5BImi‐PCNF stems from the synergistic electronic and steric advantages of the benzimidazole moiety. Compared to 5BOxa, 5BImi exerts a more potent resonance electron‐donating effect, enriching the electron density of pyridine‐like nitrogen sites to strengthen Zn^2+^ coordination. Relative to 5BThi, the imidazole ring enables more efficient 2p–2p orbital overlap, bypassing the mismatched 3p–2p interactions of thiazole, while the smaller nitrogen radius minimizes interfacial steric hindrance to enhance site accessibility [61]. Collectively, these molecular properties lower the Zn^2+^ desolvation barrier and accelerate transport kinetics, ensuring homogenized zinc deposition. Consequently, the 5BImi‐PCNF separator achieves outstanding cycling stability and rate capability that outperforms most reported separators, owing to its strong ability to suppress Zn dendrites and parasitic side reactions (Figure 3k; Table S1, Figure S25) [34, 39, 62, 63, 64, 65, 66, 67, 68, 69, 70, 71].

**FIGURE 4:**
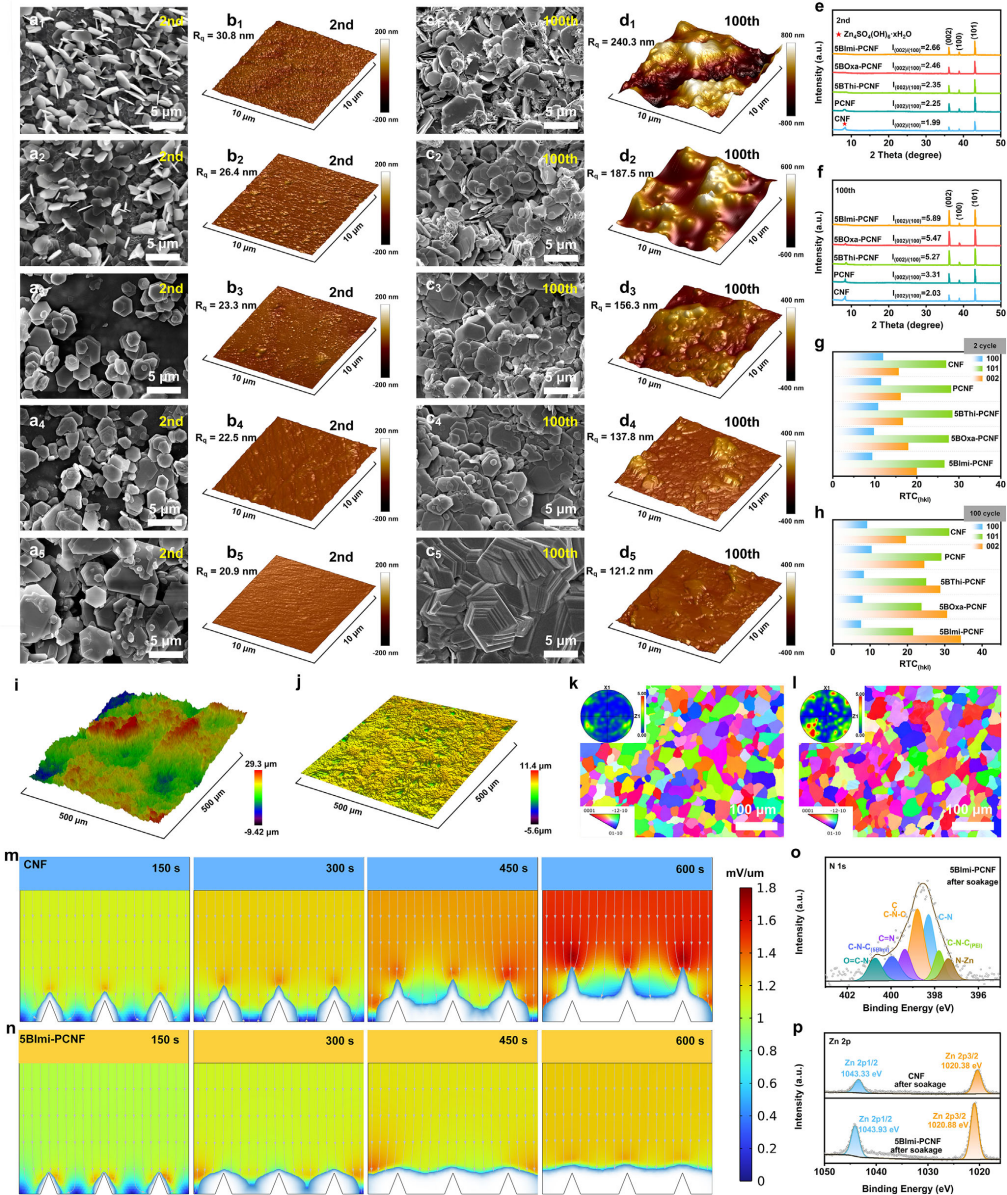
Impact of functional CNF separator on Zn plating/stripping behavior. SEM images and AFM images of the Zn anodes with a_1_‐d_1_) CNF, a_2_‐d_2_) PCNF, a_3_‐d_3_) 5BThi‐PCNF, a_4_‐d_4_) 5BOxa‐PCNF, and a_5_‐d_5_) 5BImi‐PCNF separators after various cycles at 1 mA cm^−2^ and 1 mAh cm^−2^. XRD patterns of the Zn anodes with CNF, PCNF, 5BThi‐PCNF, 5BOxa‐PCNF, and 5BImi‐PCNF separators after (e) 2 cycles and (f) 100 cycles at 1 mA cm^−2^ and 1 mAh cm^−2^. The RTC values of deposited Zn anodes with CNF, PCNF, 5BThi‐PCNF, 5BOxa‐PCNF, and 5BImi‐PCNF separators after (g) 2 cycles and (h) 100 cycles. 3D CLSM images of the cycled Zn anode with (i) CNF and (j) 5BImi‐PCNF separators. EBSD images of Zn anode after the 100^th^ cycle with (k) CNF and (l) 5BImi‐PCNF separators, respectively, inset is the corresponding polar diagrams of the EBSD image in (k) and (l), respectively. COMSOL simulations of the electric field distribution and morphological evolution with the (m) CNF and (n) 5BImi‐PCNF separators during Zn plating process. XPS analysis of (o) N 1s and (p) Zn 2p of soaked 5BImi‐PCNF separator.

To gain deeper insights into the mechanism responsible for the significant improvement in electrochemical reversibility enabled by the 5BImi‐PCNF separators, scanning electron microscopy (SEM) and atomic force microscopy (AFM) characterizations were first employed to examine the surface morphology evolution of Zn anodes after varying numbers of cycles in the Zn||Zn cells operated at 1 mA cm^−2^ and 1 mAh cm^−2^ (Figure 4a–d). Obviously, the surface of Zn anode with CNF separator is covered with numerous protruding bulges and loosely attached lamellae after the initial 2 cycles, implying the presence of non‐uniform Zn deposition and byproduct formation (Figure 4e). Over the subsequent 100 cycles, successive uneven plating/stripping processes primarily occur on these substantial protrusions and pits, which leads to the gradual accumulation of randomly oriented dendrites with sharp, blade‐like edges on the bare Zn surface. These protrusions result in significant surface height variations, which increase the risk of separator penetration, and can trigger issues such as short circuits. Quantitative AFM analysis further confirms the distinct interfacial evolution enabled by the different separators. The Zn anode with the CNF separator exhibits severe roughening, with Rq increasing from 30.8 nm after 2 cycles to 240.3 nm after 100 cycles, whereas PCNF only partially mitigates this deterioration, with Rq increasing from 26.4 to 187.5 nm. In contrast, the functionalized separators maintain smoother Zn surfaces throughout cycling, and 5BImi‐PCNF delivers the lowest roughness after prolonged cycling, with an initial Rq of 20.9 nm after 2 cycles and a final value of 121.2 nm after 100 cycles. These results indicate that 5BImi‐PCNF promotes more uniform Zn deposition and effectively suppresses interfacial roughening. The crystallographic evolution of cycled Zn anodes was also investigated through XRD (Figure 4e,f). Notably, after cycling with the 5BImi‐PCNF separator, the (001) diffraction peak corresponding to Zn_4_SO_4_(OH)_6_·xH_2_O (ZSH) byproducts is significantly weaker compared to those observed with the CNF, PCNF, 5BThi‐PCNF, and 5BOxa‐PCNF separators. These findings are further corroborated by XPS analysis (Figure S26), where no characteristic Zn 2p signals of ZSH are detected for the cycled Zn anode with 5BImi‐PCNF separator, confirming the effective suppression of interfacial side reactions. Since ZSH formation is inherently driven by water‐induced corrosion and HER, both of which elevate the local OH^−^ concentration near the Zn anode surface, its absence supports the stabilizing effect of the 5BImi‐PCNF separator at the Zn/electrolyte interface [72, 73]. Moreover, as the number of cycles increases, the I_(002)/(100)_ ratio, which represents the diffraction intensity of the Zn (002) peak relative to the Zn (100) peak, gradually rises for all five separators. Notably, the cycled Zn anode with the 5BImi‐PCNF separator exhibits a higher I_(002)/(100)_ ratio compared to the other separators, including CNF, PCNF, 5BThi‐PCNF, and 5BOxa‐PCNF (Figure S27). Additionally, the relative texture coefficient (RTC) for the (002) plane of the 5BImi‐PCNF separator increases steadily with cycling, while the RTC values for the (101) and (100) planes decrease over time (Figure 4g,h; Figure S28). These results suggest that the 5BImi‐PCNF separator effectively promotes preferential Zn growth along the (002) plane. Laser confocal scanning microscopy (LCSM) technique was further applied to intuitively investigate the surface characteristic of cycled Zn anodes with CNF and 5BImi‐PCNF separators. As depicted in Figure 4i, the 3D LCSM image of the cycled Zn with CNF reveals a distinctly coarse surface, characterized by a high surface roughness of 29.3 µm. In stark contrast, for the cycled Zn anode with the 5BImi‐PCNF separator, the corresponding surface roughness value is markedly diminished to as low as 11.4 µm, which demonstrates the ability of 5BImi‐PCNF separator to mitigate water‐induced side reactions and suppress Zn dendrite formation (Figure 4j). To further investigate this behavior, electron backscatter diffraction (EBSD) was conducted to characterize the crystallographic orientation of cycled Zn anodes. As shown in Figure 4k, the 2D color‐coded orientation map of cycled Zn anode with the CNF separator displays a poorly defined crystal structure and a low indexing rate, likely due to disorder and random Zn deposition during cycling. Conversely, the cycled Zn anode with the 5BImi‐PCNF separator clearly reveals the presence of red microscale (002) crystal domains, indicating a strong preferential orientation along the (002) plane (Figure 4l). This observation is further supported by the pole figure analysis, which shows a more intense and focused (002) signal in the 5BImi‐PCNF sample. The distinct morphological orientation is mechanistically significant, as established in prior studies, for enabling uniform Zn^2+^ ion flux distribution, consequently suppressing parasitic chemical corrosion and HER activity at the electrode‐electrolyte interface.

To gain a deep understanding of the role of the 5BImi‐PCNF separator in modulating Zn deposition behavior, finite element simulations were performed using COMSOL software to analyze the interfacial electric field dynamic (Figure 4m,n; Table S2). As the plating time increases, the electric field becomes increasingly chaotic and its intensity intensifies at the apex of the crystal Zn seeds with the CNF separator, leading to the uneven Zn deposition and random concentration distribution. However, the employment of the 5BImi‐PCNF separator contributes to a more uniform current density distribution in the localized region near the Zn electrode surface, and thus induces a more homogeneous and compact morphology than the case with the CNF separator. This improvement can be primarily attributed to the presence of imidazole groups embedded within the 5BImi‐PCNF structure, which act as ion‐mediated sites and effectively reduce the Zn^2+^ diffusion barrier, thereby facilitating efficient Zn^2+^ ion transport (Figure S29). To further validate these findings, in situ optical microscopy was employed to directly visualize the Zn plating morphologies using two different separators. As shown in Figure S30, for Zn anode with the CNF separator, distinct protrusions are observed after 15 min of plating. These irregular protrusions continue to develop throughout the plating process, clearly revealing a highly non‐uniform deposition pattern. In striking contrast, the Zn anode with the 5BImi‐PCNF separator displays an exceptionally planar and uniform deposition morphology, which demonstrates the effective suppression of protrusion formation during the plating process. The significantly improved uniformity of Zn deposition observed with the 5BImi‐PCNF separator is attributed to nitrogen‐mediated coordination chemistry. FTIR spectra collected after 21‐day immersion in 2 m ZnSO_4_ confirms the long‐term structural stability of the framework, while the characteristic redshift of the C═N vibration (∼1542 cm^−1^) reveals the dynamic coordination between nitrogen lone pairs and Zn^2+^ (Figure S31) [74]. XPS analysis show a distinct N‐Zn peak at 397.4 eV and a positive shift of the C═N binding energy from 398.7 to 399.3 eV, evidencing a significant redistribution of electron density upon Zn^2+^ anchoring at the pyridinic nitrogen sites (Figure 4o,p). Concurrently, the Zn 2p spectra displays a more intense signal for the 5BImi‐PCNF separator compared to the pristine CNF separator, indicating superior Zn^2+^ adsorption capacity. This enhancement arises from nitrogen‐containing functional groups that generate localized electron‐rich domains, which serve as preferential coordination sites for Zn^2+^ ions through electrostatic interactions [75]. It is worth noting that the enhanced Zn‐N interaction in 5BImi‐PCNF is unlikely to simply immobilize Zn^2+^. Instead, the coordinated N sites are likely to act as transient relays for Zn^2+^ transport. By promoting the partial desolvation of hydrated Zn^2+^ and stabilizing the intermediate Zn‐N interactions, the benzimidazole unit reduces the barrier to ion transfer and creates a more ordered transport pathway. This interpretation is consistent with the low migration activation energy derived from the temperature‐dependent ionic conductivity measurements (Figure S32) [76]. To further evaluate the chemical stability of the grafted functional layer, post‐cycling characterizations were conducted on separators recovered from both Zn||Zn symmetric cells. For the 5BImi‐PCNF separator cycled in a Zn||Zn cell at 20 mA cm^−2^ and 20 mAh cm^−2^ for 100 h, most characteristic FTIR and XPS signals of 5BImi‐PCNF remain essentially unchanged, whereas only the C═N band exhibits moderate shifts, consistent with Zn─N coordination rather than structural degradation (Figure S33). Consequently, the resulting coordination network in the 5BImi‐PCNF separator effectively regulates ion distribution and reduces diffusion barriers, thereby enhancing Zn^2+^ adsorption efficiency and facilitating more uniform interfacial ion transport, ultimately contributing to stable and dendrite‐free Zn deposition.

**FIGURE 5:**
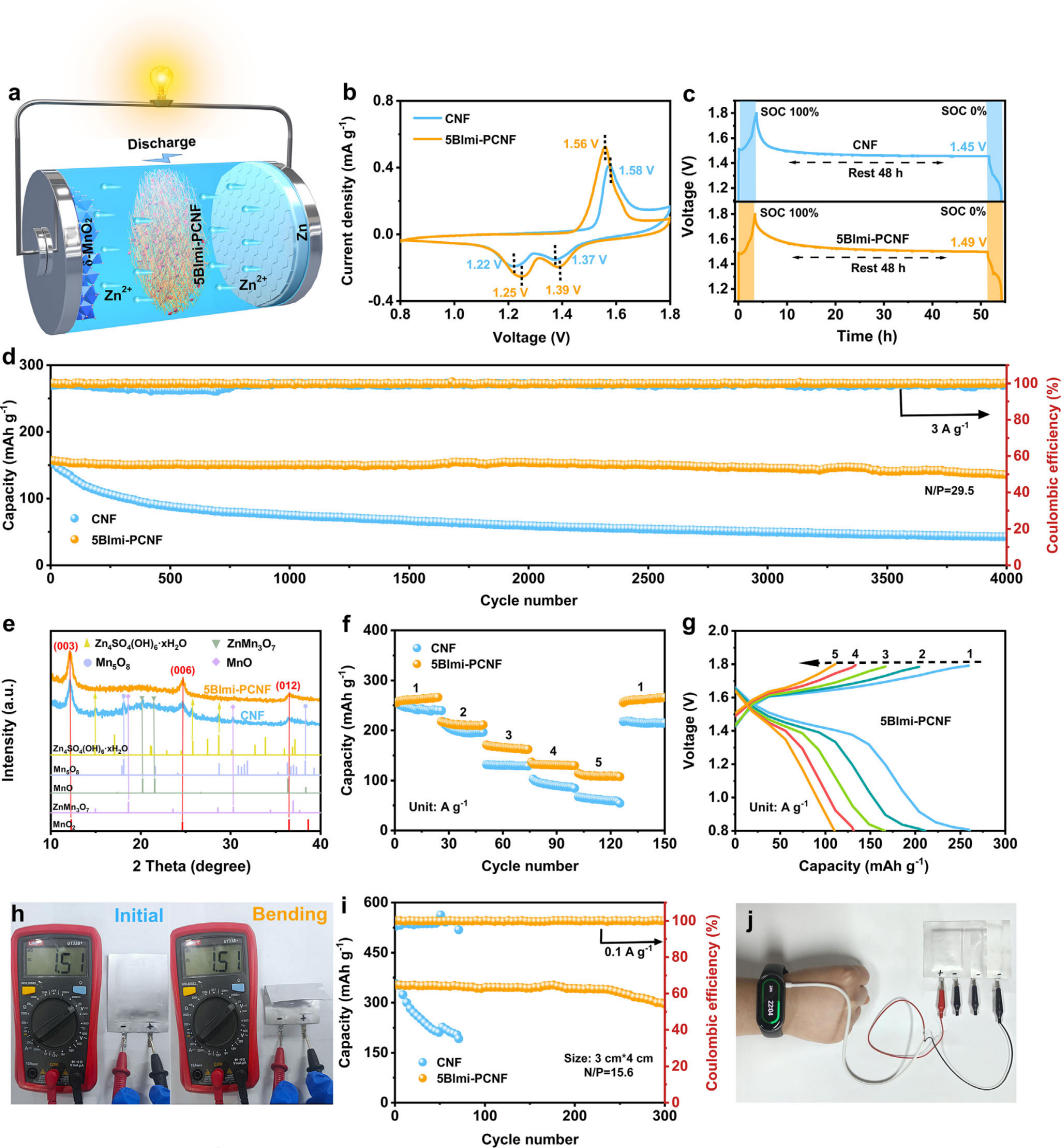
Electrochemical performance of Zn||MnO_2_ full cells. (a) Schematic diagram for the Zn||MnO_2_ full cells with 5BImi‐PCNF separator. (b) CV curves, (c) self‐discharge behavior, and (d) cycling performance of the Zn||MnO_2_ full cells with CNF and 5BImi‐PCNF separators. (e) XRD patterns of the MnO_2_ cathodes in Zn||MnO_2_ full cells assembled with the CNF and 5BImi‐PCNF separators after cycling. (f) Rate capabilities and (g) corresponding voltage‐capacity curves of Zn||MnO_2_ full cells with 5BImi‐PCNF separator at various current densities. (h) Open‐circuit voltages of the flexible Zn||MnO_2_ pouch cell under different bending conditions. (i) Cycling performance of the Zn||MnO_2_ pouch cells with the CNF and 5BImi‐PCNF separators at 0.1 A g^−1^. (j) Photo of powering an electronic watch by the pouch‐type Zn||MnO_2_ cell with 5BImi‐PCNF separator.

To further assess the electrochemical performance and practical applicability of the 5BImi‐PCNF separator, Zn||MnO_2_ full cells were fabricated with Zn foil as the anode and MnO_2_ as the cathode material (Figure 5a; Figures S34 and S35). The cyclic voltammetry (CV) curves of Zn||MnO_2_ full cells, utilizing both CNF and 5BImi‐PCNF separators, exhibit two distinct pairs of redox peaks, which correspond to the successive insertion and extraction of H^+^ and Zn^2+^ ions into/from the MnO_2_ cathode during the discharge/charge cycles (Figure 5b). Interestingly, a remarkable enhancement in self‐discharge behavior is detected upon the incorporation of 5BImi‐PCNF separator. The full cell using the 5BImi‐PCNF separator can still retain 99.2% of its original capacity, outperforming that using the CNF separator (95.3%) (Figure 5c), which suggests that the 5BImi‐PCNF effectively inhibits undesirable side reactions, such as Zn dendrite formation and Mn‐based oxide dissolution. The advantages of 5BImi‐PCNF separator were also corroborated by its exceptional capacity retention during long‐term cycling under higher current densities (Figure 5d; Figures S36 and S37). Specifically, the Zn||MnO_2_ full cell with the 5BImi‐PCNF separator can deliver an improved capacity retention of 85.8% after 4000 cycles at 3 A g^−1^. Compared with the previously reported ZIBs, the Zn||MnO_2_ full cell with the 5BImi‐PCNF separator shows a larger capacity and more extended cyclic stability, owing to the reversible plating/stripping electrochemistry induced by 5BImi‐PCNF (Figures S38–S40). Additionally, the morphological analysis shows that the cycled Zn anode with CNF separator is covered by disordered, cluttered dendrites along with accumulated particles, while the cycled Zn anode with 5BImi‐PCNF retains a smooth and uniform surface morphology, which ensures efficient and continuous transport of Zn^2+^ ions during repeated cycling (Figure S41). To further probe the effect of the separator on the cathode structural evolution, post‐cycling XRD and SEM analyses were conducted on the cycled MnO_2_ cathodes. XRD patterns reveal that the cathode paired with the CNF separator exhibits distinct diffraction peaks corresponding to irreversible parasitic phases, including Zn_4_SO_4_(OH)_6_·xH_2_O, ZnMn_3_O_7_, MnO, and Mn_5_O_8_, indicative of severe cathode dissolution, sulfate incorporation, and reductive decomposition [77, 78]. In stark contrast, these secondary phases are virtually absent in the cathode cycled with the 5BImi‐PCNF separator, demonstrating effective suppression of Mn‐based side reactions (Figure 5e). Consistently, SEM images show extensive flake‐like deposits on the cathode surface after cycling with the CNF separator, whereas the cathode cycled with the 5BImi‐PCNF separator largely preserves its initial flower‐like morphology (Figure S42). These observations suggest that the 5BImi‐PCNF separator effectively stabilizes the MnO_2_ cathode/electrolyte interface and mitigates Mn‐related side reactions during cycling. Furthermore, the Zn||MnO_2_ full cell with the 5BImi‐PCNF separator exhibits significantly enhanced rate capability compared to the CNF‐based full cell, while maintaining high energy density across a wide range of current densities (Figure 5f,g; Figures S43 and S44).

To further substantiate the application prospect of 5BImi‐PCNF separator, flexible Zn||MnO_2_ pouch cells were constructed with dimensions of ∼3 cm × 4 cm and a high active material mass loading of ∼12 mg cm^−2^. Owing to the high mechanical flexibility of the 5BImi‐PCNF separator, the pouch cell is able to maintain an open‐circuit voltage of 1.51 V even when folded at an angle of ∼90° (Figure 5h). Impressively, the pouch cell with the 5BImi‐PCNF separator delivers a high capacity retention of 83.7% after 300 cycles at 0.1 A g^−1^ (Figure 5i). In contrast, the pouch cell with CNF separator suffers from premature short‐circuiting after only 70 cycles, caused by the rampant dendrite formation and uncontrolled side reactions on the Zn anode. Furthermore, a series connection of multiple pouch cells with the 5BImi‐PCNF separator can easily power an electronic watch with a rated voltage of 3.87 V, providing clear evidence of the promising applicability of the designed ZIBs, as shown in Figure 5j. These results confirm that the 5BImi‐PCNF separator enables cells with significantly enhanced cycling stability and rate performance, with broad application prospects in the commercialization of metal batteries.

## 3 Conclusion

In summary, we have developed a CNF‐based separator with a Janus‐type electrostatic potential gradient by covalently integrating electron‐donating PEI and electron‐accepting azole moieties. The CNF‐PEI‐azole sandwich structure establishes a bidirectional electron transfer system, in which localized electron donation from PEI to both the CNF matrix and the azole surface, mediated by the interfacial dipole effect, results in a redistribution of surface charge density, thereby stabilizing the interfacial electrostatic environment. Concurrently, delocalized electron transfer from PEI to the azole groups via π‐conjugation enhances electron density at nitrogen sites and improves coordination reactivity. Combined theoretical calculations and experimental analyses reveal that the 5BImi‐PCNF exhibits the strongest Zn^2+^ coordination due to its optimized electron density distribution and favorable binding energetics relative to other azole analogues. The strengthened Zn‐N interactions accelerate Zn^2+^ transport, facilitate Zn^2+^ desolvation, and homogenize the electric field distribution, thereby promoting preferential Zn deposition along the (002) plane and significantly suppressing parasitic side reactions. Consequently, the Zn||Zn cell with the 5BImi‐PCNF separator demonstrates reduced overpotential and extended lifetimes of 5500 h at 1 mA cm^−2^ and 1 mAh cm^−2^, and maintains reliable performance even under a high current density of 20 mA cm^−2^ and a high areal capacity of 20 mAh cm^−2^. More encouragingly, when applied to Zn||MnO_2_ batteries, the utilization of the 5BImi‐PCNF separator enables a substantial capacity retention of 85.8% after 4000 cycles, along with excellent rate capability. This study puts forward a comprehensive perspective on the construction of functional separators for the protection of metal anodes, facilitating widespread application of aqueous metal batteries.

## Conflicts of Interest

The authors declare no conflicts of interest.

## Supporting information




**Supporting File**: advs75368‐sup‐0001‐SuppMat.docx.

## Data Availability

The data that support the findings of this study are available on request from the corresponding author. The data are not publicly available due to privacy or ethical restrictions.

## References

[1] F. Wang , O. Borodin , T. Gao , et al., “Highly Reversible Zinc Metal Anode for Aqueous Batteries,” Nature Materials 17 (2018): 543–549, 10.1038/s41563-018-0063-z. 29662160

[2] Y. Shang and D. Kundu , “A Path Forward for the Translational Development of Aqueous Zinc‐Ion Batteries,” Joule 7 (2023): 244–250, 10.1016/j.joule.2023.01.011.

[3] Z. Hou , Y. Gao , H. Tan , and B. Zhang , “Realizing High‐Power and High‐Capacity Zinc/Sodium Metal Anodes Through Interfacial Chemistry Regulation,” Nature Communications 12 (2021): 3083, 10.1038/s41467-021-23352-0. PMC8149847 34035276

[4] X. Zheng , Z. Liu , J. Sun , et al., “Constructing Robust Heterostructured Interface for Anode‐Free Zinc Batteries with Ultrahigh Capacities,” Nature Communications 14 (2023): 76, 10.1038/s41467-022-35630-6. PMC9816316 36604413

[5] Q. Yang , Q. Li , Z. Liu , et al., “Dendrites in Zn‐Based Batteries,” Advanced Materials 32 (2020): 2001854, 10.1002/adma.202001854. 33103828

[6] B. Li , X. Zhang , T. Wang , et al., “Interfacial Engineering Strategy for High‐Performance Zn Metal Anodes,” Nano‐Micro Letters 14 (2022): 6, 10.1007/s40820-021-00764-7. PMC8640001 34859312

[7] Q. Yang , G. Liang , Y. Guo , et al., “Do Zinc Dendrites Exist in Neutral Zinc Batteries: A Developed Electrohealing Strategy to In Situ Rescue In‐Service Batteries,” Advanced Materials 31 (2019): 1903778, 10.1002/adma.201903778. 31517400

[8] Q. Zhang , J. Luan , Y. Tang , X. Ji , and H. Wang , “Interfacial Design of Dendrite‐Free Zinc Anodes for Aqueous Zinc‐Ion Batteries,” Angewandte Chemie International Edition 59 (2020): 13180–13191, 10.1002/anie.202000162. 32124537

[9] R. Zhao , Y. Yang , G. Liu , et al., “Redirected Zn Electrodeposition by an Anti‐Corrosion Elastic Constraint for Highly Reversible Zn Anodes,” Advanced Functional Materials 31 (2021): 2001867, 10.1002/adfm.202001867.

[10] C. Li , X. Xie , H. Liu , et al., “Integrated ‘All‐in‐One’ Strategy to Stabilize Zinc Anodes for High‐Performance Zinc‐Ion Batteries,” National Science Review 9 (2022): nwab177, 10.1093/nsr/nwab177. 35265341 PMC8900688

[11] Q. Cao , Y. Gao , J. Pu , et al., “Gradient Design of Imprinted Anode for Stable Zn‐ion Batteries,” Nature Communications 14 (2023): 641, 10.1038/s41467-023-36386-3. PMC9902526 36746943

[12] Y. An , Y. Tian , H. Shen , Q. Man , S. Xiong , and J. Feng , “Two‐Dimensional MXenes for Flexible Energy Storage Devices,” Energy & Environmental Science 16 (2023): 4191–4250, 10.1039/D3EE01841E.

[13] Z. Zhao , J. Zhao , Z. Hu , et al., “Long‐Life and Deeply Rechargeable Aqueous Zn Anodes Enabled by a Multifunctional Brightener‐Inspired Interphase,” Energy & Environmental Science 12 (2019): 1938–1949, 10.1039/C9EE00596J.

[14] Z. Zhao , R. Wang , C. Peng , et al., “Horizontally Arranged Zinc Platelet Electrodeposits Modulated by Fluorinated Covalent Organic Framework Film for High‐Rate and Durable Aqueous Zinc Ion Batteries,” Nature Communications 12 (2021): 6606, 10.1038/s41467-021-26947-9. PMC8595410 34785684

[15] Y. Zhang , Y. Zhang , J. Deng , et al., “In Situ Electrochemically‐Bonded Self‐Adapting Polymeric Interface for Durable Aqueous Zinc Ion Batteries,” Advanced Functional Materials 34 (2024): 2310995, 10.1002/adfm.202310995.

[16] H. Du , Y. Dong , Q. Li , et al., “A New Zinc Salt Chemistry for Aqueous Zinc‐Metal Batteries,” Advanced Materials 35 (2023): 2210055, 10.1002/adma.202210055. 36637812

[17] H. Yang , K. Fang , J. Duan , et al., “Selective Facet Shielding Induced Epitaxial Deposition Along the Zn (101) Plane for Highly Reversible Zn‐Ion Batteries,” Energy Storage Materials 75 (2025): 103995, 10.1016/j.ensm.2024.103995.

[18] Z. Liu , R. Wang , Q. Ma , et al., “A Dual‐Functional Organic Electrolyte Additive with Regulating Suitable Overpotential for Building Highly Reversible Aqueous Zinc Ion Batteries,” Advanced Functional Materials 34 (2024): 2214538, 10.1002/adfm.202214538.

[19] Y. Shang , V. Kundi , I. Pal , et al., “Highly Potent and Low‐Volume Concentration Additives for Durable Aqueous Zinc Batteries: Machine Learning‐Enabled Performance Rationalization,” Advanced Materials 36 (2024): 2309212, 10.1002/adma.202309212. 38041711

[20] T. Xue , Y. Mu , Z. Zhang , et al., “Enhanced Zinc Deposition and Dendrite Suppression in Aqueous Zinc‐Ion Batteries Via Citric Acid‐Aspartame Electrolyte Additives,” Advanced Energy Materials 15 (2025): 2500674, 10.1002/aenm.202500674.

[21] S. Liu , J. Vongsvivut , and Y. Wang , et al., “Monolithic Phosphate Interphase for Highly Reversible and Stable Zn Metal Anode,” Angewandte Chemie International Edition 62 (2023): 202215600, 10.1002/anie.202215600. PMC10108278 36446737

[22] K. H. Shin , D. Ji , J. M. Park , Y. S. Joe , H. S. Park , and J. Kim , “Structural Composite Hydrogel Electrolytes for Flexible and Durable Zn Metal Batteries,” Advanced Functional Materials 34 (2024): 2309048, 10.1002/adfm.202309048.

[23] F. Mo , Z. Chen , G. Liang , et al., “Zwitterionic Sulfobetaine Hydrogel Electrolyte Building Separated Positive/Negative Ion Migration Channels for Aqueous Zn‐MnO_2_ Batteries with Superior Rate Capabilities,” Advanced Energy Materials 10 (2020): 2000035, 10.1002/aenm.202000035.

[24] L. Yang , M. Zhou , Y. Xie , X. Shen , S. Liang , and G. Fang , “Separators in Aqueous Zinc‐Ion Batteries: Interfacial Chemistry and Optimization Strategies,” Energy Storage Materials 67 (2024): 103271, 10.1016/j.ensm.2024.103271.

[25] Y. Zong , H. He , Y. Wang , et al., “Functionalized Separator Strategies Toward Advanced Aqueous Zinc‐Ion Batteries,” Advanced Energy Materials 13 (2023): 2300403, 10.1002/aenm.202300403.

[26] H. Ma , H. Chen , M. Chen , et al., “Biomimetic and Biodegradable Separator with High Modulus and Large Ionic Conductivity Enables Dendrite‐Free Zinc‐Ion Batteries,” Nature Communications 16 (2025): 1014, 10.1038/s41467-025-56325-8. PMC11760366 39856065

[27] Y. Su , B. Liu , Q. Zhang , et al., “Printing‐Scalable Ti_3_C_2_T_x_ MXene‐Decorated Janus Separator with Expedited Zn^2+^ Flux Toward Stabilized Zn Anodes,” Advanced Functional Materials 32 (2022): 2204306, 10.1002/adfm.202204306.

[28] Y. Lei , Q. Li , Q. Liu , et al., “Low‐Cost Separator with Dust‐Free Fabric Composite Cellulose Acetate Toward Stable Dendrite‐Free Aqueous Zinc‐Ion Batteries,” Chemical Engineering Journal 479 (2024): 147846, 10.1016/j.cej.2023.147846.

[29] R. Xue , Z. Wang , N. Yao , et al., “Multiscale Interfacial Regulation of Zn‐V_2_O_5_ Pouch Cell via Ultrathin Molecular‐Engineered Separator,” Advanced Functional Materials 34 (2024): 2400959, 10.1002/adfm.202400959.

[30] S. Liu , Q. Han , C. He , et al., “Ion‐Sieving Separator Functionalized by Natural Mineral Coating Toward Ultrastable Zn Metal Anodes,” ACS Nano 18 (2024): 25880–25892, 10.1021/acsnano.4c09678. 39236748

[31] X. Zhang , J. Li , K. Qi , et al., “An Ion‐Sieving Janus Separator Toward Planar Electrodeposition for Deeply Rechargeable Zn‐Metal Anodes,” Advanced Materials 34 (2022): 2205175, 10.1002/adma.202205175. 35901519

[32] Q. Yang , L. Li , T. Hussain , et al., “Stabilizing Interface pH by N‐Modified Graphdiyne for Dendrite‐Free and High‐Rate Aqueous Zn‐Ion Batteries,” Angewandte Chemie International Edition 61 (2022): 202112304, 10.1002/anie.202112304. 34799952

[33] C. Chen and L. Hu , “Nanocellulose Toward Advanced Energy Storage Devices: Structure and Electrochemistry,” Accounts of Chemical Research 51 (2018): 3154–3165, 10.1021/acs.accounts.8b00391. 30299086

[34] Y. Li , X. Peng , X. Li , et al., “Functional Ultrathin Separators Proactively Stabilizing Zinc Anodes for Zinc‐Based Energy Storage,” Advanced Materials 35 (2023): 2300019, 10.1002/adma.202300019. 36787635

[35] Z. Wang , P. Tammela , M. Strømme , and L. Nyholm , “Cellulose‐based Supercapacitors: Material and Performance Considerations,” Advanced Energy Materials 7 (2017): 1700130, 10.1002/aenm.201700130.

[36] H. Yu , D. Sun , W. Dong , et al., “All‐In‐One Flexible Thermoelectric Yarns for Integrated Energy Harvesting, Adaptive Personal Thermal Management, and Self‐Powered Sensing,” Advanced Functional Materials 36 (2026): 25233, 10.1002/adfm.202525233.

[37] T. Li , C. Chen , A. H. Brozena , et al., “Developing Fibrillated Cellulose as a Sustainable Technological Material,” Nature 590 (2021): 47–56, 10.1038/s41586-020-03167-7. 33536649

[38] X. Li , J. Li , Q. Yang , et al., “Homogenizing Zn2+ Transport and Deposition by Molecular Aggregation State Regulation of Cellulose Separator for High‐Performance Aqueous Zn‐Ion Batteries,” Advanced Functional Materials 36 (2025): 19947, 10.1002/adfm.202519947.

[39] S. Yang , Y. Zhang , Y. Zhang , et al., “Designing Anti‐Swelling Nanocellulose Separators with Stable and Fast Ion Transport Channels for Efficient Aqueous Zinc‐Ion Batteries,” Advanced Functional Materials 33 (2023): 2304280, 10.1002/adfm.202304280.

[40] Y. Liu , S. Liu , X. Xie , et al., “A Functionalized Separator Enables Dendrite‐Free Zn Anode Via Metal‐Polydopamine Coordination Chemistry,” Infomat 5 (2023): 12374, 10.1002/inf2.12347.

[41] B. Li , Y. Zeng , W. Zhang , et al., “Separator Designs for Aqueous Zinc‐Ion Batteries,” Science Bulletin 69 (2024): 688–703, 10.1016/j.scib.2024.01.011. 38238207

[42] H. Wu , Z. Xu , R. Cao , et al., “Multipurpose Smart Textile with Integration of Efficient Energy Harvesting, All‐Season Switchable Thermal Management and Self‐Powered Sensing,” Advanced Functional Materials 35 (2025): 09281, 10.1002/adfm.202509281.

[43] Y. Zong , H. He , Y. Wang , et al., “Functionalized Separator Strategies Toward Advanced Aqueous Zinc‐Ion Batteries,” Advanced Energy Materials 13 (2023): 2300403, 10.1002/aenm.202300403.

[44] N. Wang , S. Zhai , Y. Ma , et al., “Tridentate Citrate Chelation Towards Stable Fiber Zinc‐Polypyrrole Battery with Hybrid Mechanism,” Energy Storage Materials 43 (2021): 585–594, 10.1016/j.ensm.2021.10.004.

[45] Z. Cao , X. Zhu , S. Gao , et al., “Ultrastable Zinc Anode by Simultaneously Manipulating Solvation Sheath and Inducing Oriented Deposition With PEG Stability Promoter,” Small 18 (2022): 2103345, 10.1002/smll.202103345. 34862723

[46] Z. Miao , Q. Liu , W. Wei , et al., “Unveiling Unique Steric Effect of Threonine Additive for Highly Reversible Zn Anode,” Nano Energy 97 (2022): 107145, 10.1016/j.nanoen.2022.107145.

[47] T. Lu and Q. Chen , “A Simple Method of Identifying π Orbitals for Non‐Planar Systems and a Protocol of Studying π Electronic Structure,” Theoretical Chemistry Accounts 139 (2020): 25, 10.1007/s00214-019-2541-z.

[48] X. Shi , Z. Wang , S. Liu , et al., “Scalable Production of Carboxylated Cellulose Nanofibres Using a Green and Recyclable Solvent,” Nature Sustainability 7 (2024): 315–325, 10.1038/s41893-024-01267-0.

[49] H.‐Y. Mi , X. Jing , Q. Zheng , et al., “High‐Performance Flexible Triboelectric Nanogenerator Based on Porous Aerogels and Electrospun Nanofibers for Energy Harvesting and Sensitive Self‐Powered Sensing,” Nano Energy 48 (2018): 327–336, 10.1016/j.nanoen.2018.03.050.

[50] J. Zhao , Y. Xu , S. Ma , et al., “A Minimalist Binary Vaccine Carrier for Personalized Postoperative Cancer Vaccine Therapy,” Advanced Materials 34 (2022): 2109254, 10.1002/adma.202109254. 34984753

[51] H.‐Y. Mi , X. Jing , B. N. Napiwocki , B. S. Hagerty , G. Chen , and L.‐S. Turng , “Biocompatible, Degradable Thermoplastic Polyurethane Based on Polycaprolactone‐Block‐Polytetrahydrofuran‐Block‐Polycaprolactone Copolymers for Soft Tissue Engineering,” Journal of Materials Chemistry B 5 (2017): 4137–4151, 10.1039/C7TB00419B. 29170715 PMC5695921

[52] H. Ma , H. Chen , W. Liu , et al., “Coupling Polyethyleneimine Grafting with Macropore Filling in Ultrathin Cellulose Separator to Enable Robust Aqueous Zinc‐Based Batteries,” Journal of Energy Chemistry 114 (2026): 826–834, 10.1016/j.jechem.2025.11.001.

[53] Y. Mou , X. Wu , C. Qin , et al., “Linkage Microenvironment of Azoles‐Related Covalent Organic Frameworks Precisely Regulates Photocatalytic Generation of Hydrogen Peroxide,” Angewandte Chemie International Edition 62 (2023): 202309480, 10.1002/anie.202309480. 37462327

[54] Q. Meng , Z. Xue , S. Chen , M. Wu , and P. Lu , “Smart Antimicrobial Pickering Emulsion Stabilized by pH‐Responsive Cellulose‐Based Nanoparticles,” International Journal of Biological Macromolecules 233 (2023): 123516, 10.1016/j.ijbiomac.2023.123516. 36754260

[55] G. Wu , R. Zhu , W. Yang , et al., “Extension of Aqueous Zinc Battery Life Using a Robust and Hydrophilic Polymer Separator,” Advanced Functional Materials 34 (2024): 2316619, 10.1002/adfm.202316619.

[56] C. Li , R. Kingsbury , L. Zhou , A. Shyamsunder , K. A. Persson , and L. F. Nazar , “Tuning the Solvation Structure in Aqueous Zinc Batteries to Maximize Zn‐Ion Intercalation and Optimize Dendrite‐Free Zinc Plating,” ACS Energy Letters 7 (2022): 533–540, 10.1021/acsenergylett.1c02514.

[57] Y. Yang , H. Hua , Z. Lv , et al., “Reconstruction of Electric Double Layer for Long‐Life Aqueous Zinc Metal Batteries,” Advanced Functional Materials 33 (2023): 2212446, 10.1002/adfm.202212446.

[58] Z. Liu , R. Wang , Y. Gao , et al., “Low‐Cost Multi‐Function Electrolyte Additive Enabling Highly Stable Interfacial Chemical Environment for Highly Reversible Aqueous Zinc Ion Batteries,” Advanced Functional Materials 33 (2023): 2308463, 10.1002/adfm.202308463.

[59] Z. He , J. Guo , F. Xiong , et al., “Re‐imagining the daniell cell: Ampere‐hour‐level rechargeable Zn–Cu batteries,” Energy & Environmental Science 16 (2023): 5832–5841, 10.1039/D3EE02786D. 38076637 PMC10698845

[60] M. Yan , C. Xu , Y. Sun , H. Pan , and H. Li , “Manipulating Zn Anode Reactions Through Salt Anion Involving Hydrogen Bonding Network in Aqueous Electrolytes with PEO Additive,” Nano Energy 82 (2021): 105739, 10.1016/j.nanoen.2020.105739.

[61] J. Huang , B. Qiu , F. Xu , et al., “Steric Hindrance Manipulation in Polymer Electrolytes Toward Wide‐Temperature Solid‐State Lithium Metal Batteries,” ACS Energy Letters 10 (2025): 1921–1930, 10.1021/acsenergylett.4c03602.

[62] Q. Wang , J. Zhao , J. Zhang , et al., “Biomass Chitin Nanofiber Separators Proactively Stabilizing Zinc Anodes for Dendrite‐Free Aqueous Zinc‐Ion Batteries,” Advanced Functional Materials 34 (2024): 2405957, 10.1002/adfm.202405957.

[63] H. Ma , J. Yu , M. Chen , et al., “Amino‐Enabled Desolvation Sieving Effect Realizes Dendrite‐Inhibiting Thin Separator for Durable Aqueous Zinc‐Ion Batteries,” Advanced Functional Materials 33 (2023): 2307384, 10.1002/adfm.202307384.

[64] Z. Zheng , S. Guo , M. Yan , Y. Luo , and F. Cao , “A Functional Janus Ag Nanowires/Bacterial Cellulose Separator for High‐Performance Dendrite‐Free Zinc Anode Under Harsh Conditions,” Advanced Materials 35 (2023): 2304667, 10.1002/adma.202304667. 37730093

[65] Y. Chen , G. Zhou , X. Huang , et al., “Alleviating Salt Depletion at the Zinc Anode Interface by an Ion‐Releasing Separator to Achieve Ultra‐Stable Zinc Anode,” Energy Storage Materials 78 (2025): 104247, 10.1016/j.ensm.2025.104247.

[66] K. Zhu , L. Wu , C. Guo , et al., “Multiscale Ion‐Sieving Separator with Selective Zn^2+^ Channels and Excellent Zn^2+^ Desolvation Kinetics for Dendrite‐Free and Kinetics‐Enhanced Zinc Metal Batteries,” Advanced Functional Materials 33 (2023): 2305098, 10.1002/adfm.202305098.

[67] Y. Zhang , Z. Liu , X. Li , L. Fan , Y. Shuai , and N. Zhang , “Loosening Zinc Ions from Separator Boosts Stable Zn Plating/Striping Behavior for Aqueous Zinc Ion Batteries,” Advanced Energy Materials 13 (2023): 2302126, 10.1002/aenm.202302126.

[68] J. Cao , D. Zhang , C. Gu , et al., “Manipulating Crystallographic Orientation of Zinc Deposition for Dendrite‐free Zinc Ion Batteries,” Advanced Energy Materials 11 (2021): 2101299, 10.1002/aenm.202101299.

[69] Y. Wu , M. Xie , K. Fu , et al., “Realizing Lean‐Electrolyte Zinc‐Ion Batteries via An Ultrathin and Cost‐Effective Separator,” Advanced Functional Materials 36 (2025): 27567, 10.1002/adfm.202527567.

[70] Y. Tan , D. Chen , T. Yao , et al., “Tailoring Zn^2+^ Flux by an Ion Acceleration Layer Modified Separator for High‐Rate Long‐Lasting Zn Metal Anodes,” Advanced Science 11 (2024): 2407410, 10.1002/advs.202407410. 39377257 PMC11600266

[71] S. Liu , Q. Han , C. He , et al., “Ion‐Sieving Separator Functionalized by Natural Mineral Coating Toward Ultrastable Zn Metal Anodes,” ACS Nano 18 (2024): 25880–25892, 10.1021/acsnano.4c09678. 39236748

[72] R. Yao , L. Qian , Y. Sui , et al., “A Versatile Cation Additive Enabled Highly Reversible Zinc Metal Anode,” Advanced Energy Materials 12 (2022): 2102780, 10.1002/aenm.202102780.

[73] F. Wang , H. Lu , H. Zhu , et al., “Mitigating the Interfacial Concentration Gradient by Negatively Charged Quantum Dots Toward Dendrite‐Free Zn Anodes,” Energy Storage Materials 58 (2023): 215–221, 10.1016/j.ensm.2023.03.032.

[74] F. Chen , K. Zhang , Y. Yuan , et al., “Ion‐Conductive Metallo‐Covalent Organic Frameworks Constructed with Tridentate Ligand and Zn Nodes,” Journal of the American Chemical Society 145 (2023): 25341–25351, 10.1021/jacs.3c09114. 37956115

[75] J. Cao , X. Rao , S. Qian , et al., “Dynamic Zn^2+^‐Coordinating Oxygen Sites and Electric Field Modulation in Boron‐Integrated Cellulose Nanofiber Separators for Stable Zinc‐Ion Batteries,” Advanced Energy Materials 15 (2025): 03368, 10.1002/aenm.202503368.

[76] C. Lin , S.‐H. Kim , Q. Xu , et al., “High‐Voltage Asymmetric Metal–Air Batteries Based on Polymeric Single‐Zn^2+^‐Ion Conductor,” Matter 4 (2021): 1287–1304, 10.1016/j.matt.2021.01.004.

[77] M. Chen , M. Yang , X. Han , J. Chen , P. Zhang , and C.‐P. Wong , “Suppressing Rampant and Vertical Deposition of Cathode Intermediate Product via PH Regulation Toward Large‐Capacity and High‐Durability Zn//MnO_2_ Batteries,” Advanced Materials 36 (2024): 2304997, 10.1002/adma.202304997. 37707488

[78] J. Chen , M. Chen , H. Chen , et al., “Wood‐Inspired Anisotropic Hydrogel Electrolyte with Large Modulus and Low Tortuosity Realizing Durable Dendrite‐Free Zinc‐Ion Batteries,” Proceedings of the National Academy of Sciences 121 (2024): 2322944121, 10.1073/pnas.2322944121. PMC11126919 38748586

