# Supplementary material for: Janus‐Type Electrostatic Potential Gradient‐Activated Dynamic Zn2+‐Coordinating Nitrogen Sites in Molecularly Locked Nanocellulose Separators for Stable Zinc‐Ion Batteries
Source: Adv Sci (Weinh). 2026 Apr 29;13(39):e75368. doi: 10.1002/advs.75368 (PMC13335745; doi:10.1002/advs.75368)
Supplement: Supplementary file 1 — Supporting File: advs75368‐sup‐0001‐SuppMat.docx. [file ADVS-13-e75368-s001.docx]

Copyright WILEY-VCH Verlag GmbH & Co. KGaA, 69469 Weinheim, Germany, 2016.

Supporting Information

**Janus-Type Electrostatic Potential Gradient-Activated Dynamic Zn^2+^-Coordinating Nitrogen Sites in Molecularly Locked Nanocellulose Separators for Stable Zinc-Ion Batteries**

*Jie Liang, Shuxin Yang, Shuaiqi Zhao, Haitao Yang, Jingyuan Li, Jiaming Dong, Junwen Duan, Hao Yang, Yue Li, Yaxin Wang, Meilin Li, Ying Liu*, Zhitao Shen, Rong Liu, Ruirui Cao*, Fumin Li, Minshen Zhu*, Yang Huang**

1. Liang, S. Yang, S. Zhao, H. Yang, J. Li, J. Dong, J. Duan, H. Yang, Dr. Y. Liu, Dr. Z. Shen, Dr. R. Liu, Dr. R. Cao, Dr. F. Li
2. Henan Key Laboratory of Quantum Materials and Quantum Energy, School of Quantum Information Future Technology, Henan University, Kaifeng, 475000 China

E-mail: yliu344@outlook.com; rrcao0403@vip.henu.edu.cn

1. Wang, M. Li, Dr. Y. Huang

Advanced Materials Thrust, The Hong Kong University of Science and Technology (Guangzhou), Guangzhou, 511400 China

1. mail: [yanghuang@hkust-gz.edu.cn](mailto:yanghuang@hkust-gz.edu.cn)

Y. Li, Dr. M. Zhu

Research Center for Materials, Architectures and Integration of Nanomembranes (MAIN), Chemnitz University of Technology, 09126 Chemnitz, Germany

E-mail: [minshen.zhu@main.tu-chemnitz.de](mailto:minshen.zhu@main.tu-chemnitz.de)

**Experimental Section**

*Materials*: Bleached kraft pulp, sourced from polar wood, was utilized as the initial raw material to prepare CNF. Citric acid (≥99.5%) was purchased from Shanghai Maclean Biochemical Co. Choline chloride (≥98%), N-hydroxysuccinimide (NHS), 1-ethyl-3-(3-dimethylaminopropyl) carbodiimide (EDC), N-methy1-2-pyrrolidone (NMP, 99.5%), MnSO_4_·H_2_O (99%), ZnSO_4_·7H_2_O (99%), 5-benzimidazolecarboxylic acid (5BImi), benzothiazole-5-carboxylic acid (5BThi), and 5-benzoxazolecarboxylic acid (5BOxa) were purchased from Shanghai Aladdin Biochemical Technology Co. Branched polyethyleneimine was purchased from Alfa Aesar. KMnO_4_ was obtained from Nanjing Reagent. Polyvinylidene fluoride (PVDF), carbon black and aluminum plastic packaging film were procured from Canrd. Zn, Ti, and Cu foils were provided by Qinghe Haoxuan Metal Materials Co., Ltd.

*Preparation of C-CNF*: Citric acid, choline chloride, and deionized water were meticulously mixed in a 3:1:1 mass ratio and heated to 80 °C for 30 min to produce a homogeneous, transparent liquid, designated as H-DES.^[1]^ Subsequently, 0.45 g of bleached kraft pulp was slowly introduced into a round-bottom flask with 10 mL of the pre-prepared H-DES solution. The mixture was then subjected to reflux at 130 °C for 3 h under continuous stirring. After the pre-treatment process, the pulp was separated and thoroughly washed with deionized water. Finally, the resulting product was ultrasonically dispersed to yield carboxylated carbon nanofiber (C-CNF) solution.

*Preparation of PCNF*: 4.6 mg of NHS was dissolved in 2 mL of deionized water. This solution was then added to 30 mL of the C-CNF solution, followed by stirring for 15 min. Subsequently, 7.6 mg of EDC and PEI (10% of the weight of C-CNF), which had been dissolved in 4 mL of deionized water, were introduced into the C-CNF solution. The mixture was then stirred for an additional 5 min to initiate the grafting reaction.^[2]^ After the reaction was completed, the product was washed with deionized water until the pH reached neutral. Finally, it was ultrasonically dispersed to obtain polyethyleneimine-grafted cellulose nanofibers (PCNF) solution.

*Preparation of azole derivatives end-capped CNF (M-PCNF)*: M-PCNF were synthesized via an amide condensation reaction, using EDC·HCl and NHS as the condensation agents. In a typical synthesis, 2 mmol of azole molecules (either 5BImi, 5BThi, or 5BOxa individually), 4 mmol of NHS, and 3 mmol of EDC·HCl were dissolved in 10 mL of DMSO and stirred for 1 h. Subsequently, 10 mL of PCNFs was added and stirred vigorously for another 48 h at 25 ℃. Following this, the mixture was precipitated using diethyl ether, and the viscous product was collected after evaporating excess diethyl ether.^[3]^ To further purify the product, gradient dialysis was conducted with deionized water, adjusting the pH from 3 to 7. Finally, a fluffy solid product was obtained after lyophilization and punched into 20 mm discs for use as separators.

*Preparation of positive electrode materials*: The δ-MnO_2_ positive electrode was synthesized via a previously reported hydrothermal method.^[4]^ Specifically, 0.95 g of KMnO_4_ was dissolved in 60 mL of deionized water, and 0.17 g of MnSO_4_·H_2_O was dissolved in 20 mL of DI water. The MnSO_4_·H_2_O solution was then added dropwise to the KMnO_4_ solution under continuous stirring for 1 h. Subsequently, the resulting mixture was transferred into a 100 mL Teflon-lined stainless steel autoclave and heated at 160 °C for 12 h. After cooling to room temperature, the black δ-MnO_2_ precipitate was collected through centrifugation, and then dried under vacuum at 80 °C overnight. The cathode slurry was fabricated by thoroughly mixing δ-MnO_2_ powder (70 wt%), carbon black (20 wt%), and PVDF (10 wt%) in NMP, then cast on a carbon paper. The electrodes, containing 12 mg cm^-2^ of δ-MnO_2_, were punched into 10 mm discs after being dried at 80 ℃ overnight.

*Materials characterization*: The morphological and structural characteristics of the separators and electrodes were investigated using a field emission scanning electron microscope (FE-SEM, JEOL JSM-7001F) and a 3D laser confocal scanning microscope (LCSM, JEOL JSM-7500F). The phase composition of the Zn deposits was analyzed by X-ray diffraction (XRD, Bruker D8 Advance). The functional groups and molecular interactions within the separators were investigated using Fourier transform infrared spectra (FTIR, PerkinElmer Spectrum 100) over the wavenumber range of 200-4000 cm^-1^. The chemical composition of the separators was examined through X-ray photoelectron spectroscopy (XPS) using an ESCALAB 250Xi system (Thermo Fisher Scientific). The tensile properties of the separators were measured with SANS EUT2203 universal testing machine. The wettability of the separators in ZnSO₄ electrolyte was assessed using an optical contact angle measurement system (Kruss DSA30). The surface topography of cycled Zn electrodes using different separators was analyzed via atomic force microscopy (AFM, Bruker Dimension ICON).

*Electrochemical Characterization*: Zn||Zn symmetric and Zn||Cu asymmetric cells were assembled using 2 M ZnSO_4_ aqueous electrolyte, CNF, P-CNF, 5BThi-PCNF, 5BOxa-PCNF or 5BImi-PCNF separator, and Zn foil as the negative electrode. For each cell, 80 µL of electrolyte was added to the separator. Zn||MnO_2_ batteries were fabricated with δ-MnO_2_ positive electrode, Zn foil negative electrode, and 2 M ZnSO_4_ + 0.1 M MnSO_4_ aqueous electrolyte*.* All electrochemical measurements, such as electrochemical impedance spectroscopy (EIS), linear sweep voltammetry (LSV), linear polarization (LP) curves, and cyclic voltammetry (CV) were performed using a multichannel LAND battery testing system (CT3001A) at a controlled temperature of 25℃. For exsitu analysis, the coin cells were first disassembled to retrieve the electrodes, which were then washed with deionized water and naturally dried in ambient air.

*Density functional theory calculations*: All DFT calculations were executed on the Vienna Ab Simulation Initio Package (VASP).^[5,6]^ These calculations were based on the generalized gradient approximation (GGA), specifically adopting the Perdew-Burke-Ernzerhof (PBE) functional. The interaction between the ionic cores and valence electrons was modeled using the projector augmented wave (PAW) method. A plane-wave basis set with a kinetic energy cutoff of 450 eV was used throughout the simulations. To address the partial occupancies of the Kohn-Sham orbitals, the Gaussian smearing technique was applied with a smearing width of 0.05 eV. Geometry optimizations were considered converged when the forces on the atoms were below 0.02 eV Å^-1^ and the energy difference between steps was smaller than 1×10^-5^ eV. To minimize interlayer interactions, a vacuum layer of 18 Å was introduced perpendicular to the slab surface. All structural optimization were conducted using a gamma-centered k-point grid of 1 × 1 × 1. The weak interaction was modeled using the DFT + D3 method, incorporating the empirical correction based on Grimme's scheme. The electrostatic potential (ESP) of different separators was analyzed using the Multiwfn package for calculation and the VMD package for visualization. The adsorption energy (*E_ads_*) was calculated based on the following formula:

*E_ads_ = E_total_* *– E_substrate_ – E*_adsorbate_

where *E_total_* is the energy of absorption structure, *E_substrate_* is the energy of the isolated substrate, and *E_adsorbate_* is the energy of adsorbate.

*Finite element simulations*: Finite element analysis was conducted using COMSOL Multiphysics with the "Tertiary Current Distribution" and "Phase Field" modules to investigate the dynamic behavior of Zn deposition on electrodes with different separators.^[7,8]^ The dimensions of the entire two-dimensional model were specified as 5.0 × 5.0 μm. A transient simulation of the process was performed in a region filled with 2 M ZnSO_4_ electrolyte. The transfer of Zn^2+^ ions was simulated according to Fick's law. A galvanostatic boundary condition was applied at a constant current density of 1 mA cm^-2^ and transient simulations were performed for more than 400 s to ensure that the system reached steady-state conditions. The ionic conductivity and Zn^2+^ diffusion coefficient were assigned to 2.52 mS cm^-1^ and 1 × 10^-11^ m^2^ s^-1^ for CNF, compared to 5.80 mS cm^-1^ and 5 × 10^-11^ m^2^ s^-1^ for 5BImi-PCNF, respectively. The interfacial overpotentials were further calibrated to 0.047 V for CNF and 0.058 V for 5BImi-PCNF. All simulation parameters are summarized in Table S1. The relationship between the diffusion coefficient and the electric mobility, the equilibrium potential of the electrode surface, and the reaction at the electrode surface were modeled using the Nernst-Einstein relation, the Nernst equation, and the Butler-Volmer kinetics expression, respectively. In this system, the two phases and three components were differentiated using a non-conserved order parameter, *ξ*, where *ξ* = 0 represented the electrolyte and *ξ* = 1 corresponded to the Zn metal electrode. The concentration of each species was denoted as *c_i_* (with *i* = Zn, Zn^2+^, and SO_4_^2-^). The local electrostatic potential was represented by *ϕ_i_*, where *i* = Zn and e, indicating the Zn metal electrode and the electrolyte, respectively. Additionally, the displacement field was denoted by *u*. The total free energy of this system was expressed by the following equation:

$$\text{F}\text{=}\int_{\text{V}} \text{ }\left[ \text{ }\text{f}_{\text{grad}}\text{ }\text{(}\text{ξ}\text{) + }\text{f}_{\text{ch}\text{ }}\text{(}\text{ξ}\text{,}\text{c}_{\text{i}}\text{) + }\text{f}_{\text{elec}}\text{ }\text{(}\text{ξ}\text{,}\text{c}_{\text{i}}\text{,}\text{ϕ}_{\text{i}}\text{) +}{\text{ }\text{f}}_{\text{els}}\text{ }\text{(}\text{ξ}\text{,}\text{u}\text{)} \right]\text{d}\text{V}$$

where *f_grad_*, *f_ch_*, *f_elec_*, and *f_els_* are the local energy density from the gradient, chemical, electrostatic, and elastic contributions, respectively. The additional equations utilized in the computational simulations of this work were derived from those proposed in previous reports.

**Supporting Tables**

**Table S1.** Comparison of the lifespans of Zn anodes.


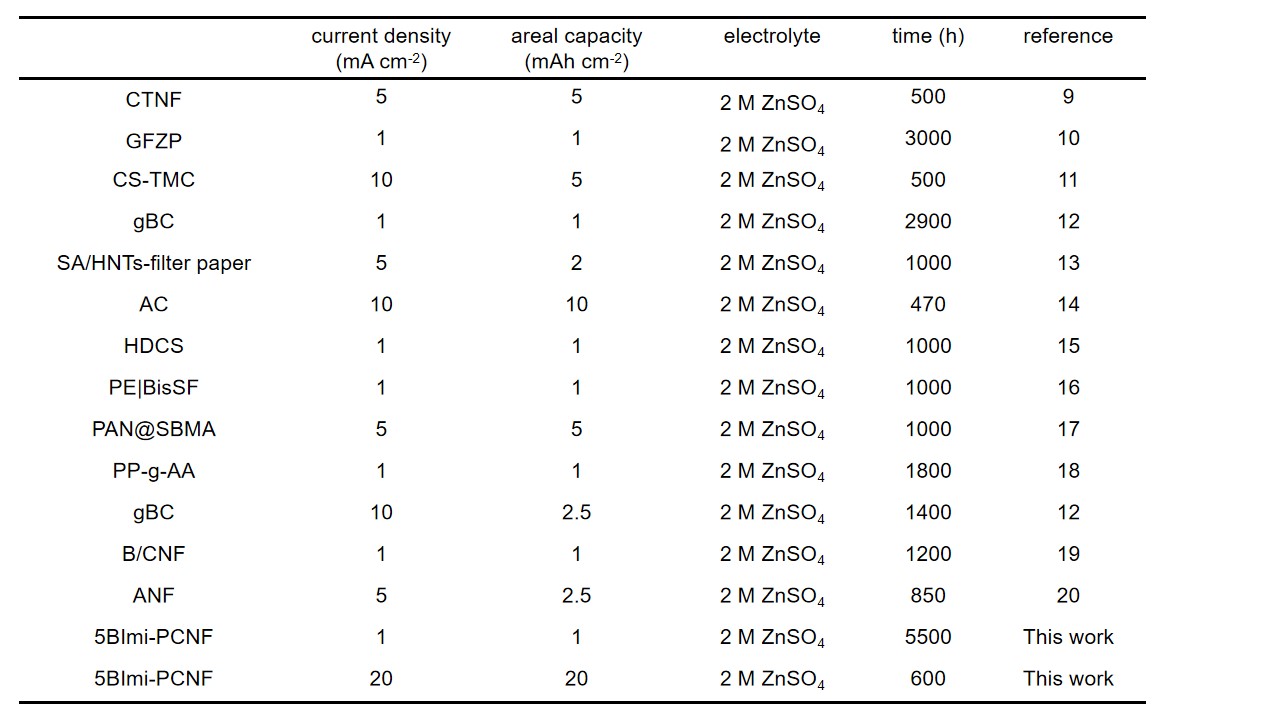


As summarized in Table S1, the Zn||Zn symmetric cell using the 5BImi‑PCNF separator delivers a significantly extended cycle life, outperforming several recently reported counterparts such as CTNF, GFZP, CS-TMC, gBC, SA/HNTs-filter paper, AC, HDCS, PE|BisSF, PAN@SBMA, PP-g-AA, B/CNF and ANF-based systems.^[9–20]^ This superior durability is primarily ascribed to the enhanced mechanical robustness and optimized Zn^2+^-conducting channels of the 5BImi-PCNF framework, which collectively ensure rapid ion transport and maintain interfacial integrity throughout extended cycling.

**Table S2.** The relevant parameters employed in the COMSOL simulations.

**
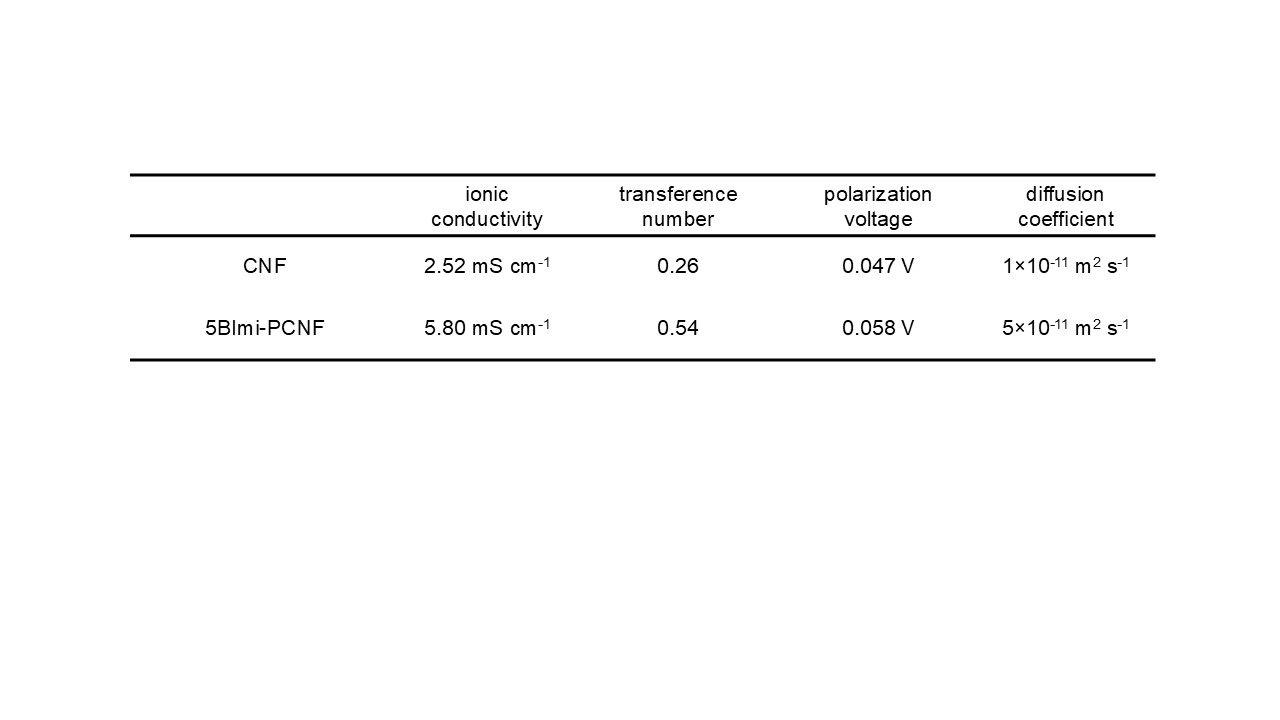
**

**Supporting Figures**


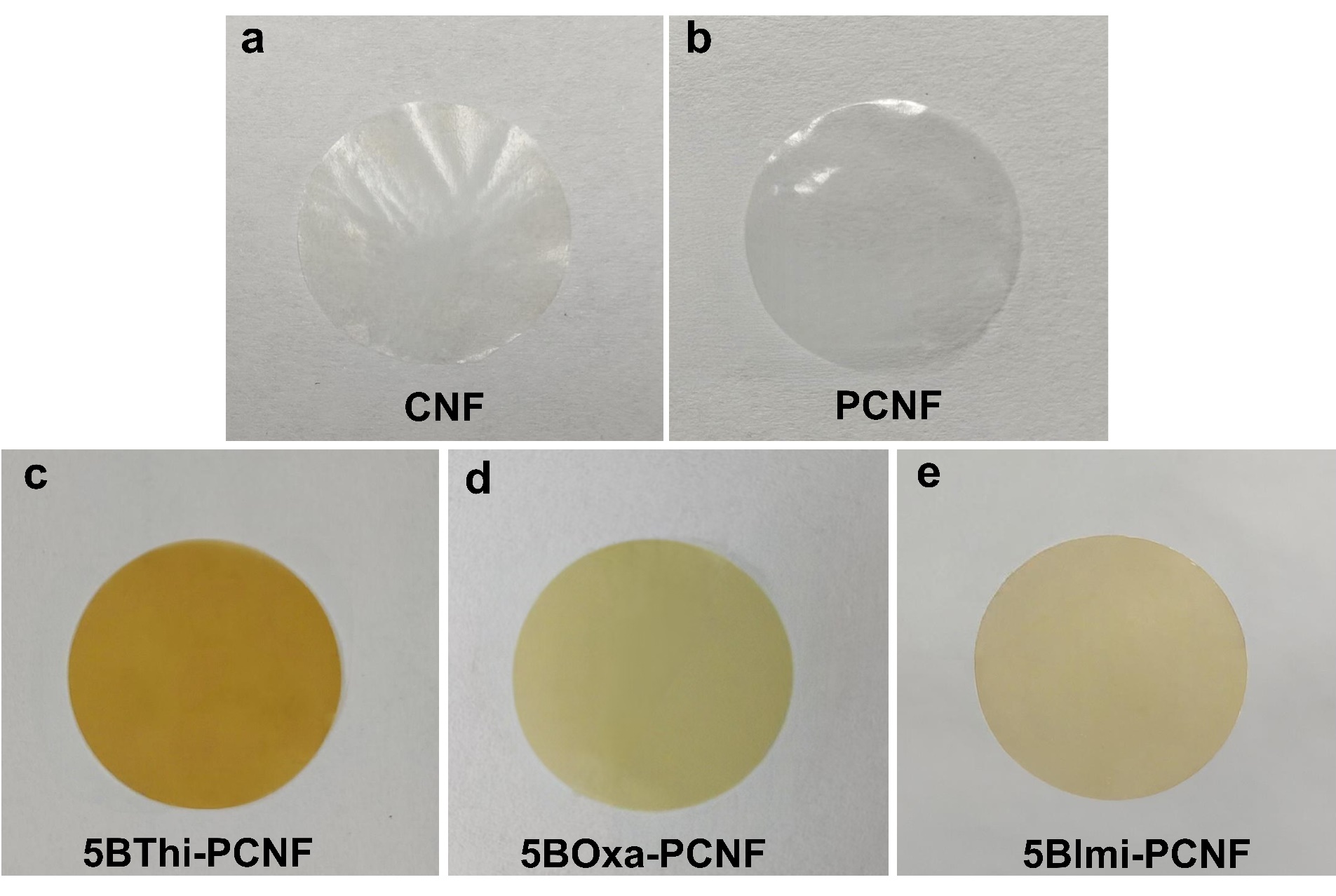


**Figure S1.** Photographs of (a) CNF, (b) P-CNF, (c) 5BThi-PCNF, (d) 5BOxa-PCNF, and (e) 5BImi-PCNF separators.

As shown in Figure S1, the 5BThi-PCNF, 5BOxa-PCNF, and 5BImi-PCNF separators exhibit distinct coloration compared to the pristine CNF and PCNF, indicating the successful introduction of the azole groups. Moreover, the uniform distribution of colors across the membrane suggests that the surface modifications are homogeneous.





**Figure S2.** (a) FTIR spectra, (b) XRD pattern, and (c) cross-sectional SEM image of the C-CNF separator.

As illustrated in Figure S2, the FTIR spectrum confirms the successful incorporation of the carboxyl groups, as evidenced by the C=O stretching vibrations observed at 1,735 cm^-1^. In addition, XRD analysis demonstrates that the characteristic diffraction peaks of CNF at 16.2° and 22.6°, corresponding to the (110) and (200) crystal planes, respectively, are retained in the modified C-CNF. These findings indicate that the modification process does not alter the crystalline structure of CNF, thereby preserving its overall chemical integrity. Cross-sectional SEM imaging confirms that the C-CNF separator possesses a uniform thickness of approximately 35.8 μm. The H-DES treatment significantly improves the dispersion and homogeneity of the nanofiber, establishing a robust structural foundation for subsequent functionalization.

**
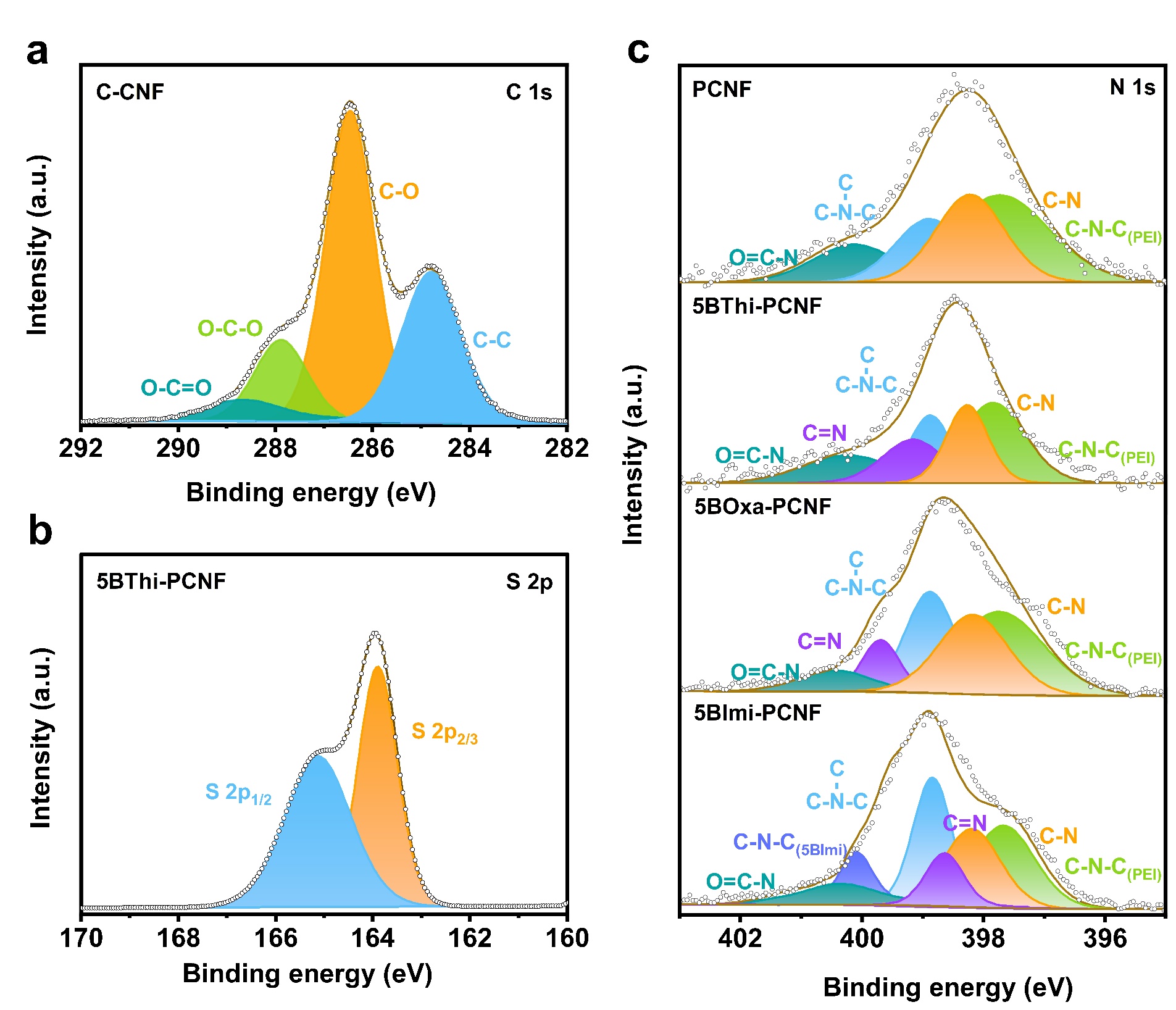
**

**Figure S3.** (a) High-resolution C 1s spectra of the CNF separator. (b) High-resolution S2p spectra of the 5BThi-PCNF separator. (c) High-resolution N 1s spectra of PCNF, 5BThi-PCNF, 5BOxa-PCNF, and 5BImi-PCNF separators.


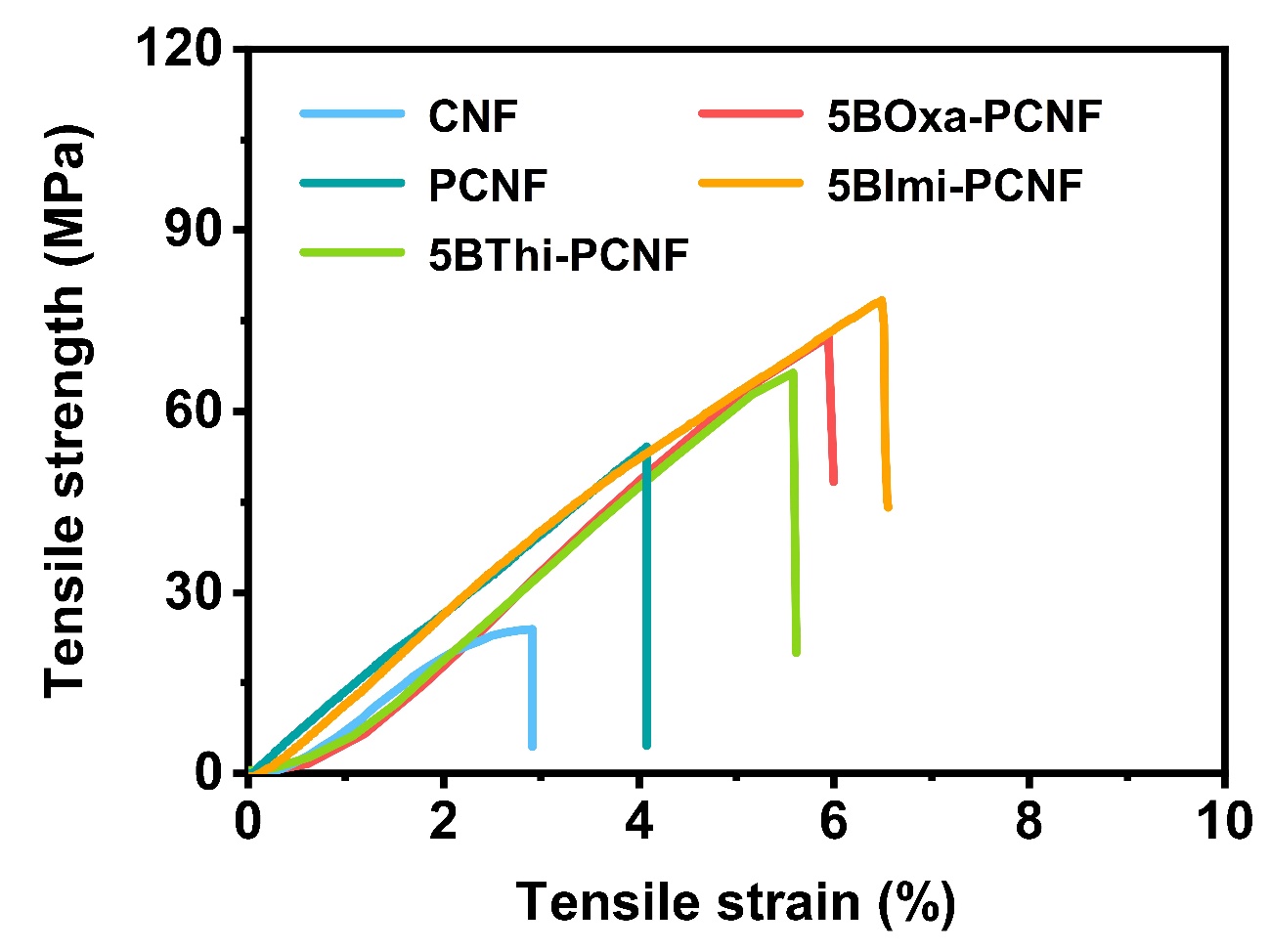


**Figure S4.** The wet-state stress-strain curves of the CNF, PCNF, 5BThi-PCNF, 5BOxa-PCNF, and 5BImi-PCNF separators after infiltrating with the aqueous electrolyte.


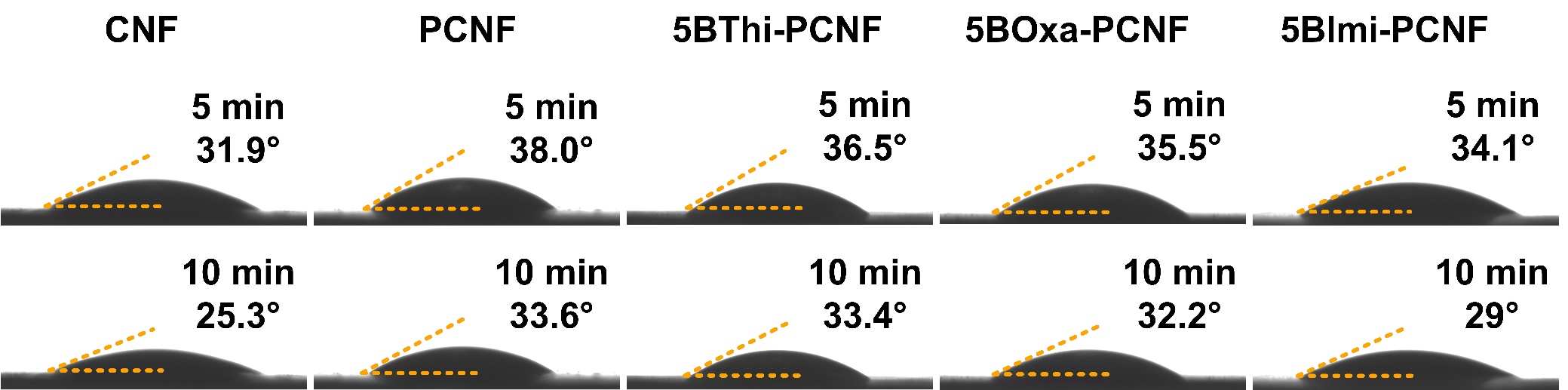


**Figure S5.** Contact angle tests for CNF, PCNF, 5BThi-PCNF, 5BOxa-PCNF, and 5BImi-PCNF separators.

As illustrated in Figure S5, the contact angle between the 5BImi-PCNF separator and the 2M ZnSO_4_ electrolyte decreases with increasing soaking time up to 10 min, and becomes lower than that observed for the PCNF, 5BThi-PCNF, and 5BOxa-PCNF separators. This result indicates that the 5BImi-PCNF separator exhibits improved wettability, which is likely due to its enhanced adsorption capacity.


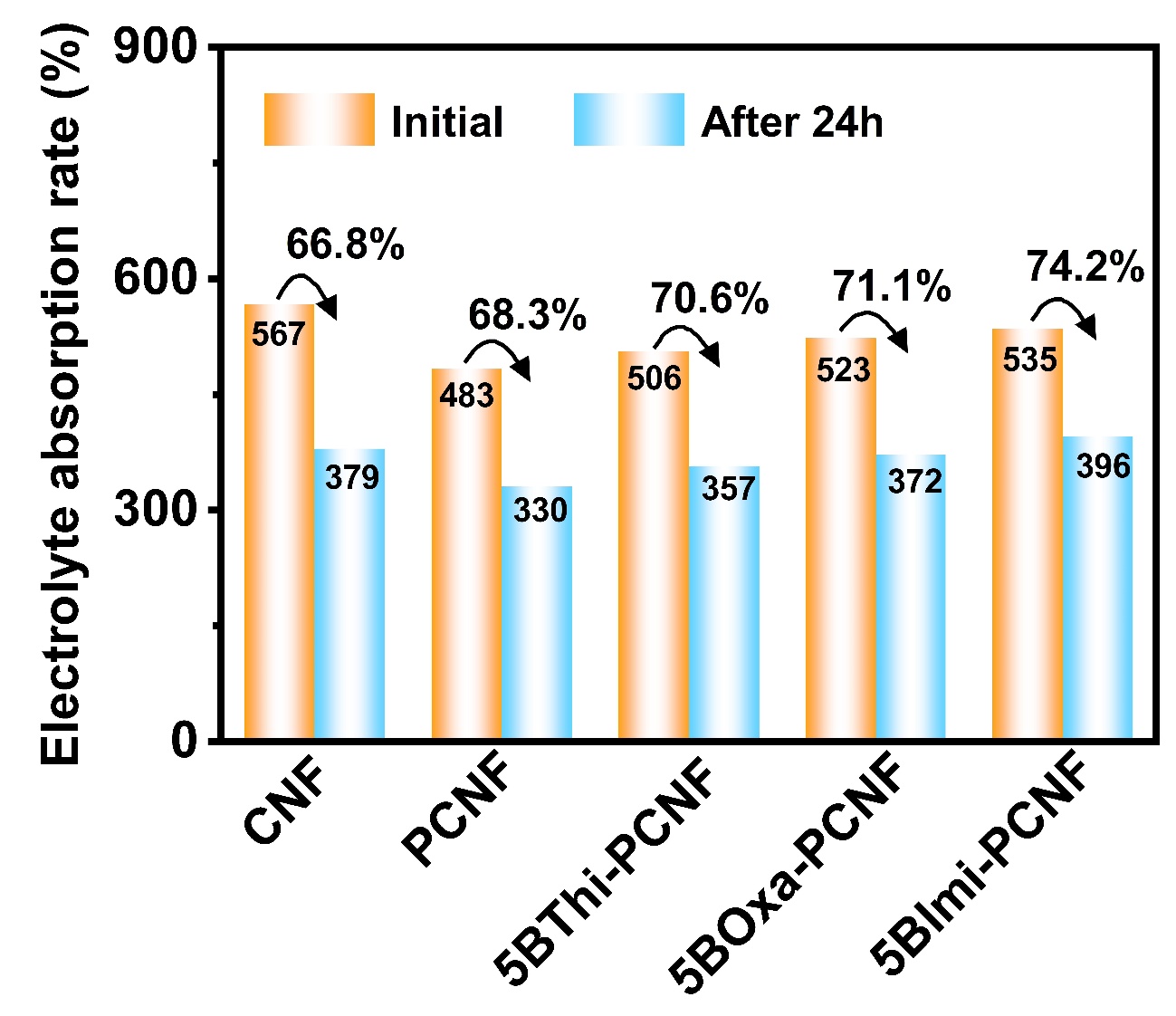


**Figure S6.** The electrolyte absorption percentages and the electrolyte retention rates after 24 h of the CNF, PCNF, 5BThi-PCNF, 5BOxa-PCNF, and 5BImi-PCNF separators.

As shown in Figure S6, all separators display excellent electrolyte absorption capabilities with absorption rates exceeding 100%, thus fulfilling the requirements for practical applications in electrochemical systems. Notably, after 24 h at a constant temperature of 25 °C, the 5BImi-PCNF separator exhibits the highest electrolyte retention rate of 74.2%, surpassing those of CNF (66.8%), PCNF (68.3%), 5BThi-PCNF (70.6%), and 5BOxa-PCNF (71.1%). Such an enhanced retention capability clearly indicates that the 5BImi-PCNF separator is exquisitely tailored for stable operation under conditions of limited electrolyte availability.


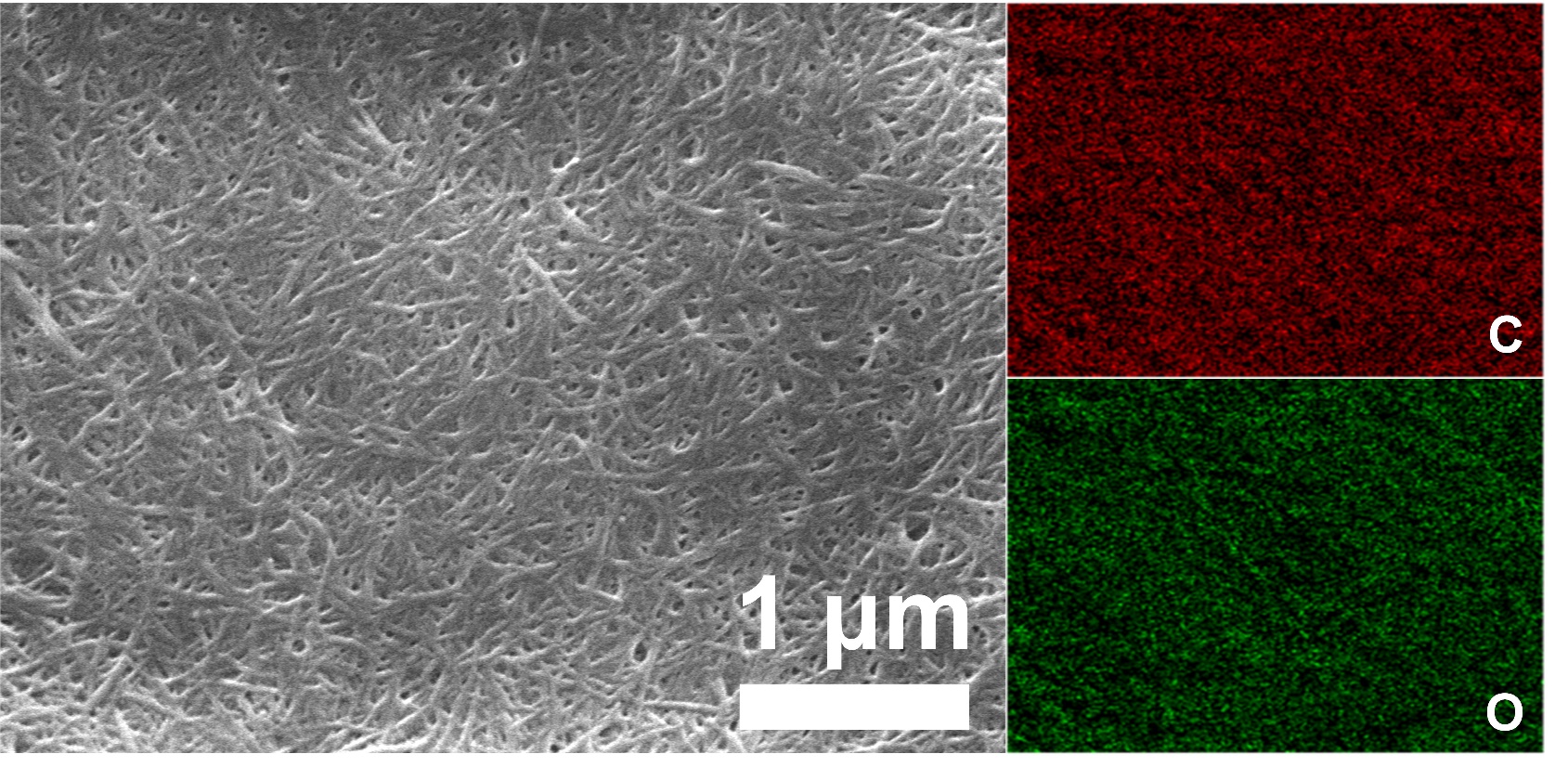


**Figure S7.** SEM image and corresponding EDS mapping images of the CNF separator.

As illustrated in Figure S7, the as-prepared CNF separator possesses a densely entangled nanofiber network structure. Elemental mapping conducted via energy-dispersive X-ray spectroscopy (EDS) confirms a uniform spatial distribution of carbon (C) and oxygen (O) across the entire nanofiber matrix, thereby substantiating compositional homogeneity and the successful synthesis of the CNF separator.





**Figure S8.** Diameter distribution histograms of the (a) CNF, (b) C-CNF, (c) PCNF, (d) 5BThi-PCNF, (e) 5BOxa-PCNF, and (f) 5BImi-PCNF separators.

As illustrated in Figure S8, the pristine CNF displays fiber diameters ranging from approximately 10 to 35 nm, with an average diameter of 19.8 nm. Carboxylation leads to an improved fiber dispersion and a slight reduction of the average diameter to 17.7 nm. Following surface grafting with PEI, the average fiber diameter increases to 23.4 nm, which indicates successful conjugation of PEI. Further functionalization with azole-based groups results in a slight increase of the average diameter to approximately 25.0 nm. This progressive increase in fiber dimension provides compelling evidence for the stepwise covalent immobilization of both PEI and azole moieties onto the CNF backbone.





**Figure S9.** Pore size distribution profiles of the (a) CNF, (b) C-CNF, (c) PCNF, (d) 5BThi-PCNF, (e) 5BOxa-PCNF, and (f) 5BImi-PCNF separators.

As shown in Figure S9, the CNF separator displays a narrow pore size distribution, with over 90% of the pores ranging between 20 and 140 nm. The average pore diameter is approximately 74 nm, suggesting a moderate porosity that promotes efficient electrolyte infiltration while effectively suppressing Zn dendrite formation. While carboxylation slightly increases the average pore diameter to 77 nm due to improved fiber dispersion, subsequent PEI grafting leads to a more compact microstructure with an average pore size of 58 nm. The integration of the azole functional group into the PCNF matrix, as exemplified by the variants 5BThi-PCNF, 5BOxa-PCNF, and 5BImi-PCNF, results in a further reduction in pore size, with the majority of pores measured to be predominantly below 50 nm. This structural change can be attributed to the steric hindrance and enhanced intermolecular interactions introduced by the azole moieties, which reduce the fiber spacing and compress the porous network.


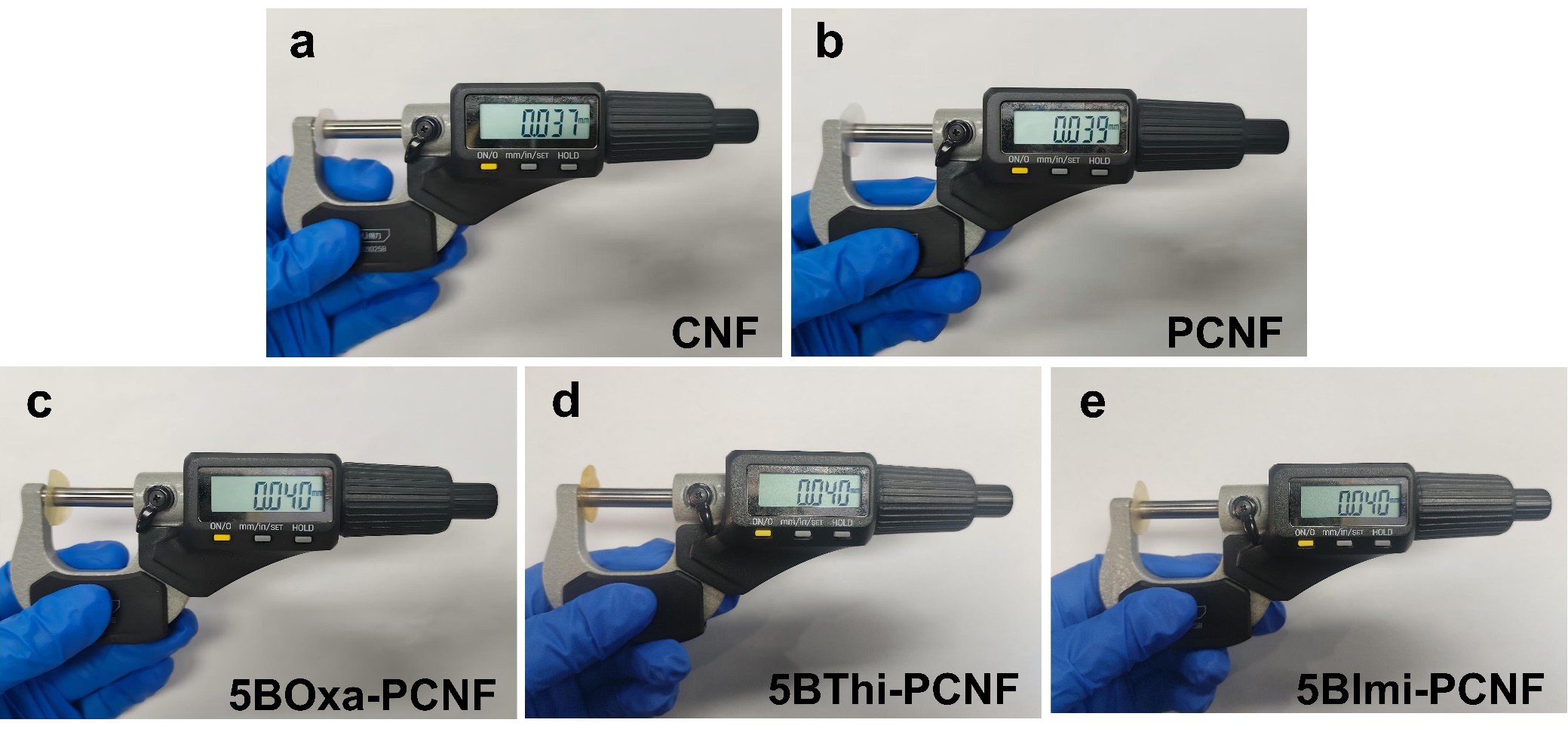


**Figure S10.** Digital images showing the thickness of the (a) CNF, (b) PCNF, (c) 5BThi-PCNF, (d) 5BOxa-PCNF, and (e) 5BImi-PCNF separators.

As presented in Figure S10, the CNF separator exhibits a thickness of 37 μm. Upon modification, the thicknesses of the PCNF, 5BThi-PCNF, 5BOxa-PCNF, and 5BImi-PCNF separators increase to ~ 40 μm. This progressive increase in thickness is primarily attributed to the grafting of functional groups, which promotes the formation of branched polymer structures on the fiber surface, thereby enhancing the overall bulk of the separator.


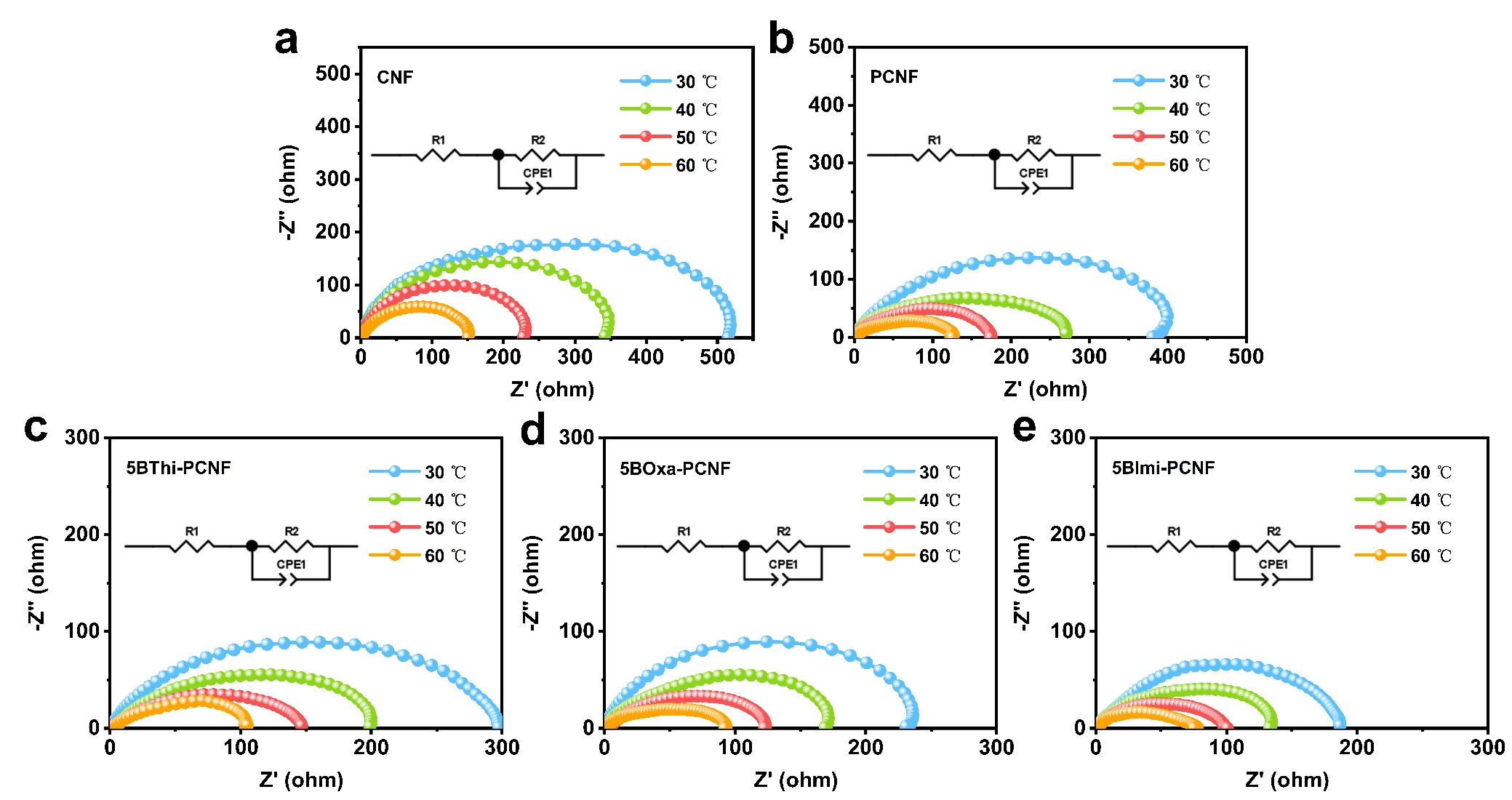


**Figure S11.** EIS spectra of Zn||Zn symmetric cells with (a) the CNF, (b) PCNF, (c) 5BThi-PCNF, (d) 5BOxa-PCNF, and (e) 5BImi-PCNF separators at different temperatures.

As shown in Figure S11, the impedance of Zn||Zn symmetric cells using the five different separators decreases progressively with increasing temperature, indicating enhanced reaction kinetics at elevated temperatures. Through activation energy calculations based on the impedance data, it was revealed that the 5BImi-PCNF separator demonstrates the lowest desolvation activation energy (21.9 kJ mol^-1^), compared to CNF (36.1 kJ mol^-1^), PCNF (32.8 kJ mol^-1^), 5BThi-PCNF (29.0 kJ mol^-1^), and 5BOxa-PCNF (26.5 kJ mol^-1^), further clearly confirming that the 5BImi-PCNF separator more effectively facilitates the desolvation process and the subsequent reduction of hydrated zinc ions.


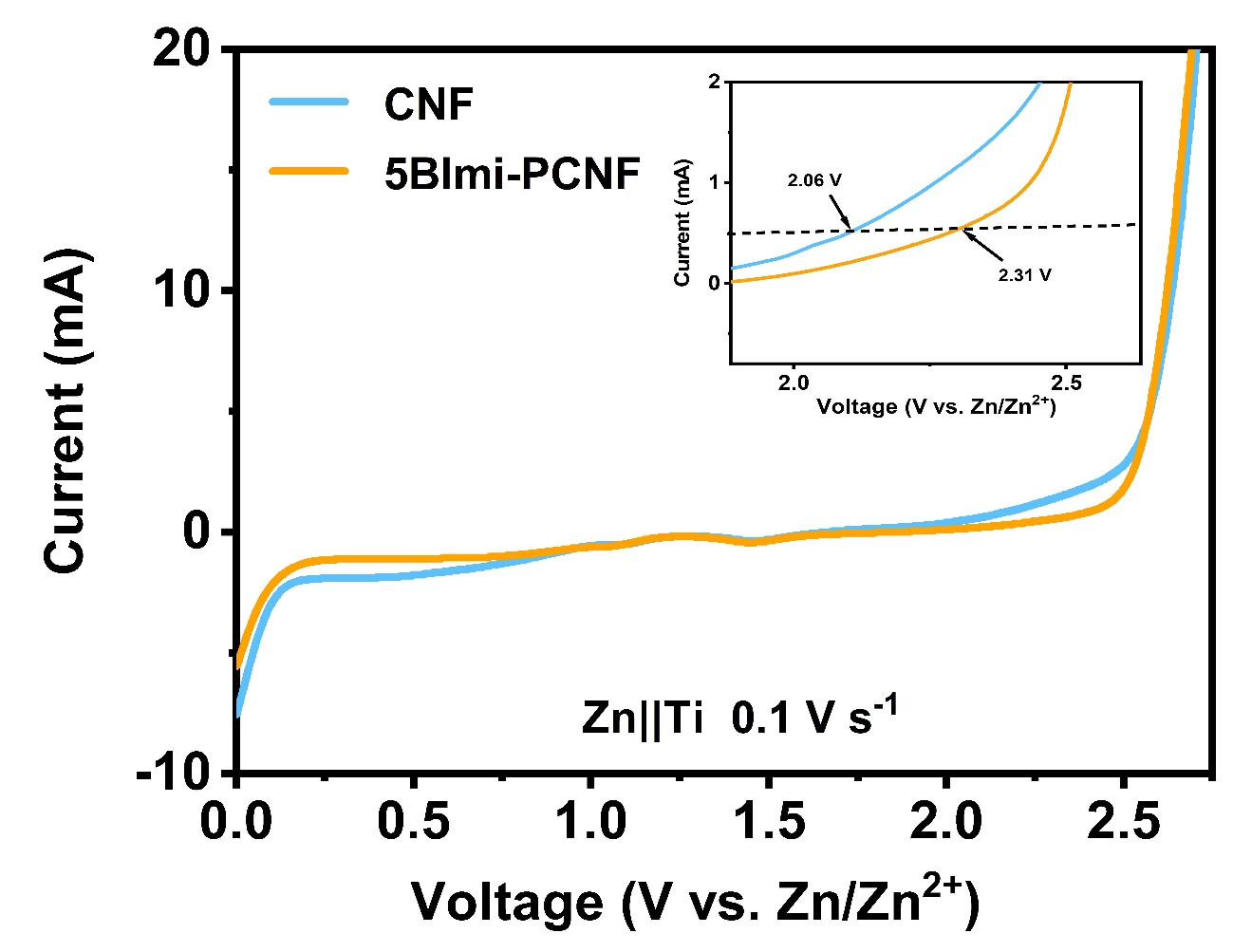


**Figure S12.** LSV curves of Zn||Ti asymmetric cells with the CNF and 5BImi-PCNF separators.

As shown in Figure S12, even after functionalization with imidazole-based molecules, the modified CNF separator displays a significantly broadened electrochemical stability window. This enhancement demonstrates that the imidazole-functionalized modification not only maintains the intrinsic structural integrity of the CNF matrix but also effectively suppresses parasitic side reactions at elevated potentials through improved interfacial charge distribution and reduced electrolyte decomposition.


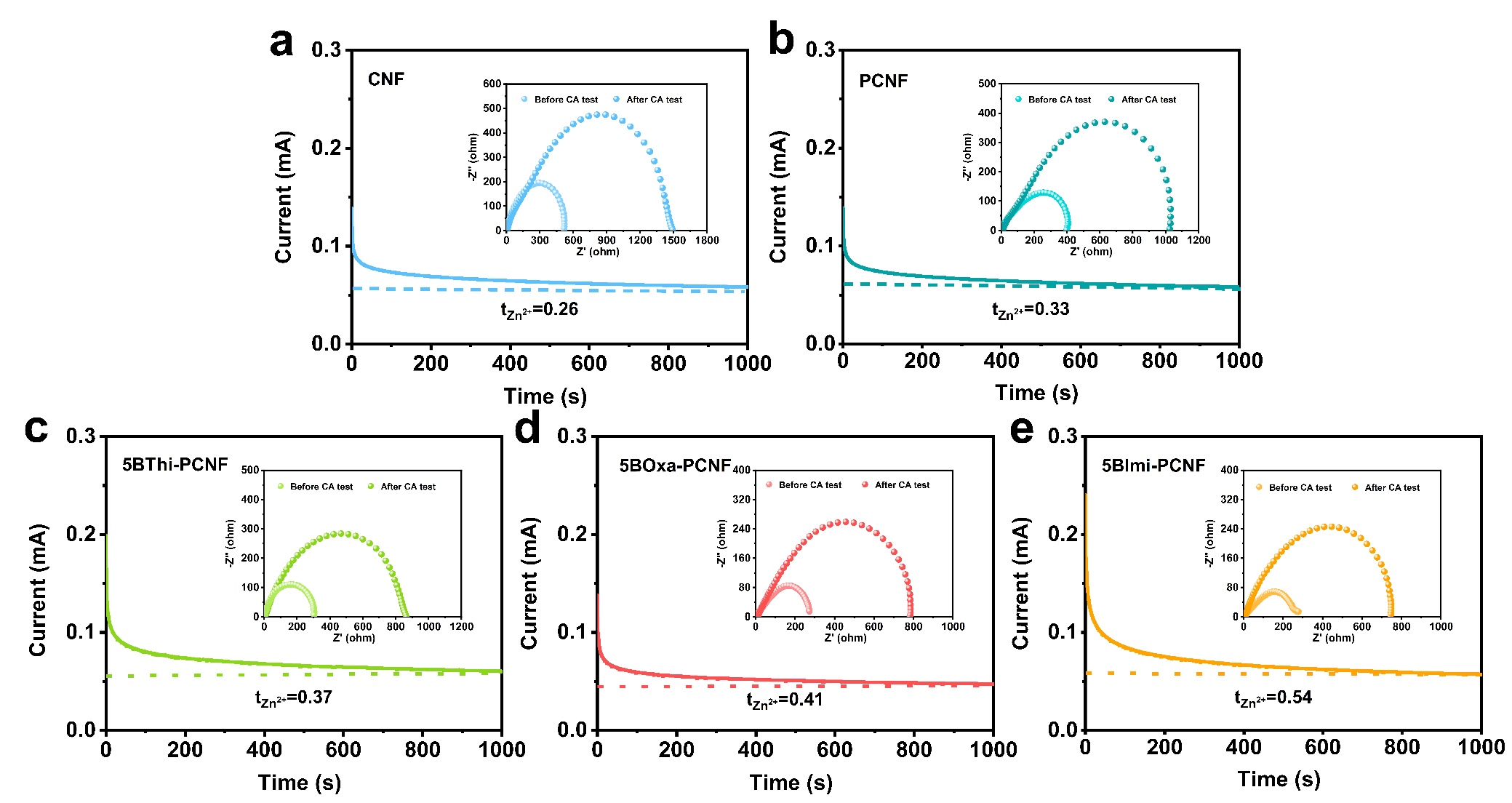


**Figure S13.** CA tests of Zn||Zn symmetric cells with (a) the CNF, (b) PCNF, (c) 5BThi-PCNF, (d) 5BOxa-PCNF and (e) 5BImi-PCNF separators. Insets show EIS spectra of these symmetric cells before and after CA tests.

As shown in Figure S13, the Zn^2+^ transference number of the Zn||Zn symmetric cells using the 5BImi-PCNF separator is 0.54, much higher than those with the CNF separator (0.26), PCNF separator (0.33), 5BThi-PCNF separator (0.37) and 5BOxa-PCNF separator (0.41). This elevated transference number helps to reduce concentration polarization, thereby effectively suppressing zinc dendrite growth.


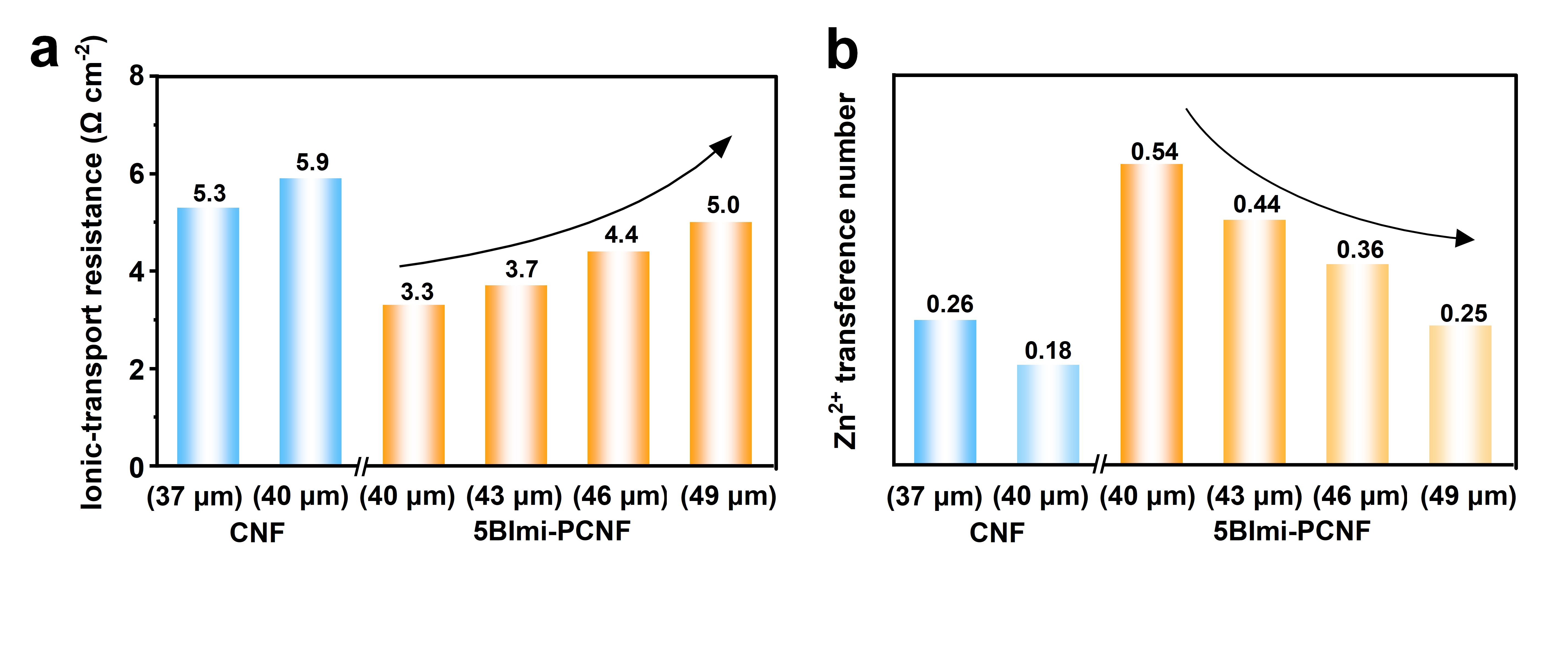


**Figure S14**. (a) EIS spectra and (b) Zn^2+^ transference numbers for CNF and 5BImI-PCNF separators across various thicknesses.

As shown in Figure S14, increasing the thickness of the CNF separator from 37 to 40 μm causes the bulk resistance (R_s_) to rise from 5.3 to 5.9 Ω cm^-2^ and the Zn^2+^ transference number to decrease from 0.26 to 0.18. By contrast, the 5BImi-PCNF modified separator, maintained at the same 40 μm thickness, exhibits a markedly lower R_s_ of 3.3 Ω cm^-2^ and a substantially higher Zn^2+^ transference number of 0.54, demonstrating superior ion transport kinetics and enhanced Zn^2+^ selectivity compared with the unmodified CNF. When the thickness of the 5BImi-PCNF separator is further increased to 43, 46, and 49 μm, the R_s_ rises progressively to 3.7, 4.4, and 5.0 Ω cm^-2^, while the Zn^2+^ transference number declines correspondingly to 0.44, 0.36, and 0.25. These systematic variations underscore the fundamental trade-off between separator thickness and ionic transport efficiency, arising from increased bulk resistance and intensified electrode polarization. Therefore, these results confirm that 5BImi functionalization effectively reduces interfacial resistance and enhances Zn^2+^ selectivity, with peak performance observed at approximately 40 μm.


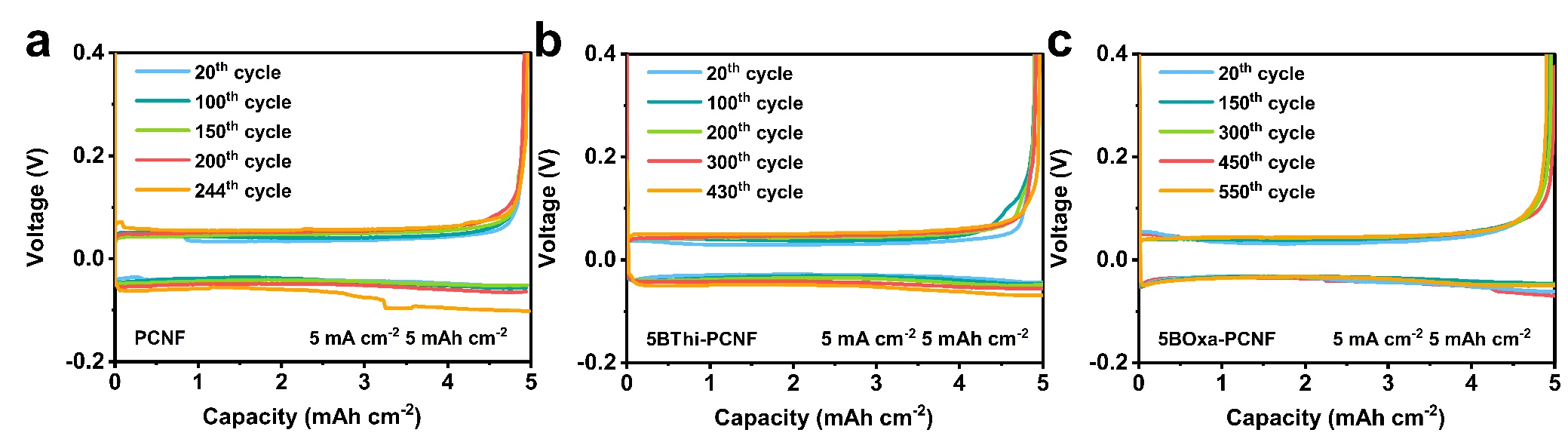


**Figure S15.** The capacity-voltage profiles of Zn||Cu cells with the (a) PCNF, (b) 5BThi-PCNF, and (c) 5BOxa-PCNF separators at various cycles.

As shown in Figure S15, the Zn||Cu cell with the CNF separator suffers from poor stability after 244 cycles, whereas the cells with the 5BThi-PCNF and 5Boxa-PCNF separators exhibit stable voltage profiles after 430 and 550 cycles, respectively.


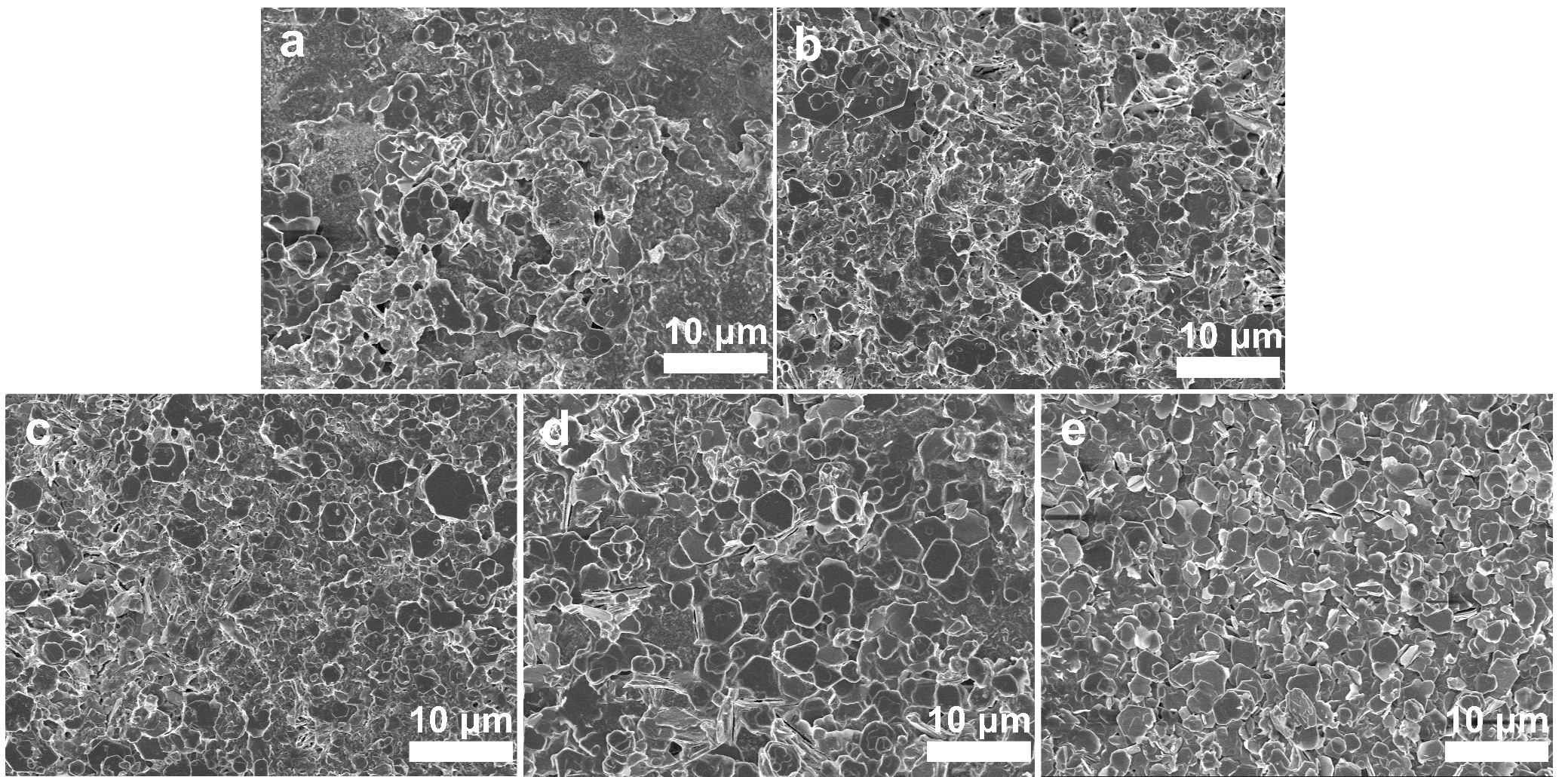


**Figure S16.** SEM images of Cu electrodes from Zn||Cu cells with the (a) CNF, (b) PCNF, (c) 5BThi-PCNF, (d) 5BOxa-PCNF, and (e) 5BImi-PCNF separators after Zn deposition for 1 h at 5 mA cm^-2^.

As shown in Figure S16, the Zn^2+^ deposition on the Cu electrode surface with the CNF separator gives rise to remarkable aggregation, which leads to the formation of irregular, loosely packed dendrites, accompanied by disordered flake-like structures. In contrast, the use of PCNF, 5BThi-PCNF, and 5BOxa-PCNF separators promotes more uniform Zn deposition, resulting in smoother surfaces with fewer byproducts and more consistent coverage. Notably, with the 5BImi-PCNF separator, Zn^2+^ deposition is highly uniform, forming flat, densely packed flakes with reduced surface roughness. This morphology is indicative of Zn growth along the (002) crystallographic plane, which is characteristic of stable, compact Zn deposition.


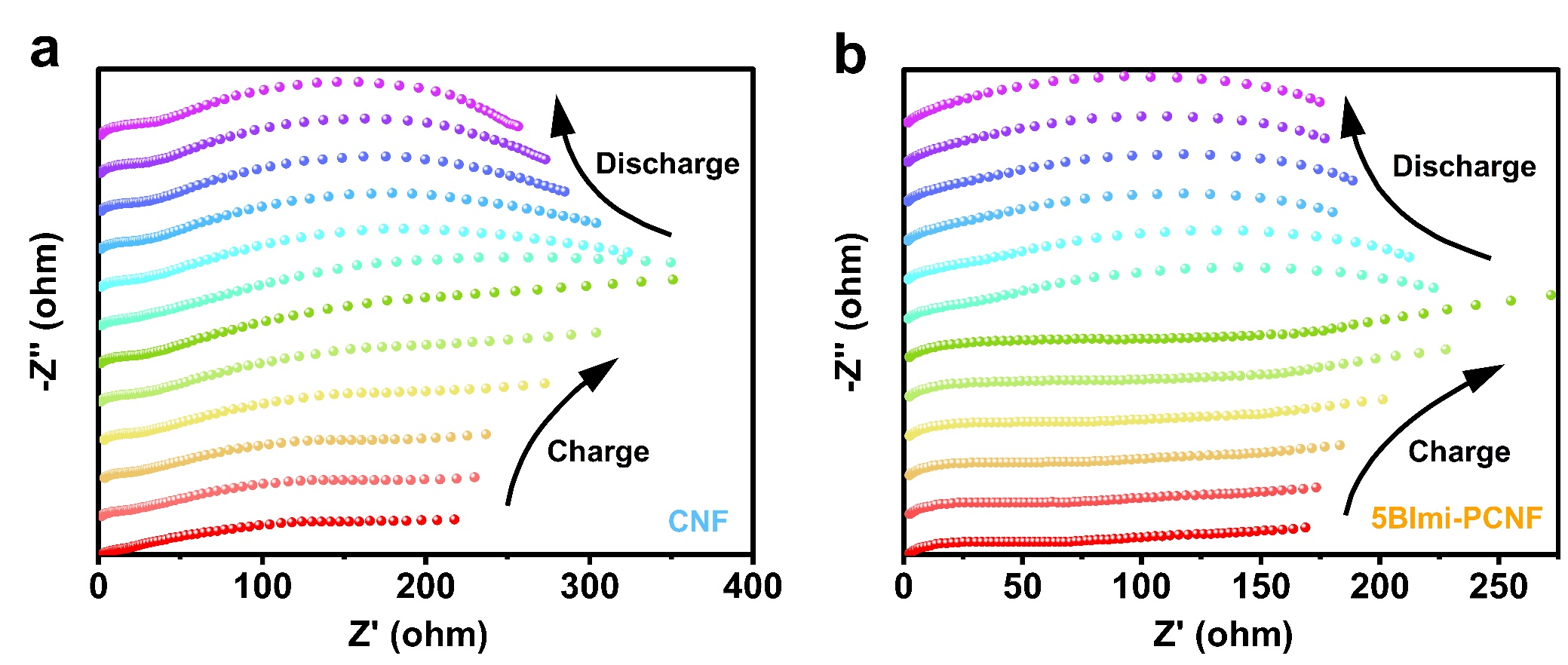


**Figure S17.** In situ EIS of Zn||Zn symmetric cells with (a) the CNF and (b) 5BImi-PCNF separators at the 3^rd^ cycle.

To further investigate the interfacial electrochemical stability and the evolution of Zn^2+^ transport kinetics, in situ electrochemical impedance spectroscopy (EIS) was conducted on symmetric cells. As illustrated in Figure S17, the CNF separator demonstrates a notable increase in interfacial impedance, which arises from the sluggish electrochemical reduction kinetics of Zn^2+^ ions, resulting in non-uniform Zn deposition. In contrast, the 5BImi-PCNF separator maintains a significantly lower interfacial impedance throughout the cycling process, which can be attributed to the enhanced zincophilicity of the separator, thereby promoting Zn^2+^ transport and facilitating uniform deposition.


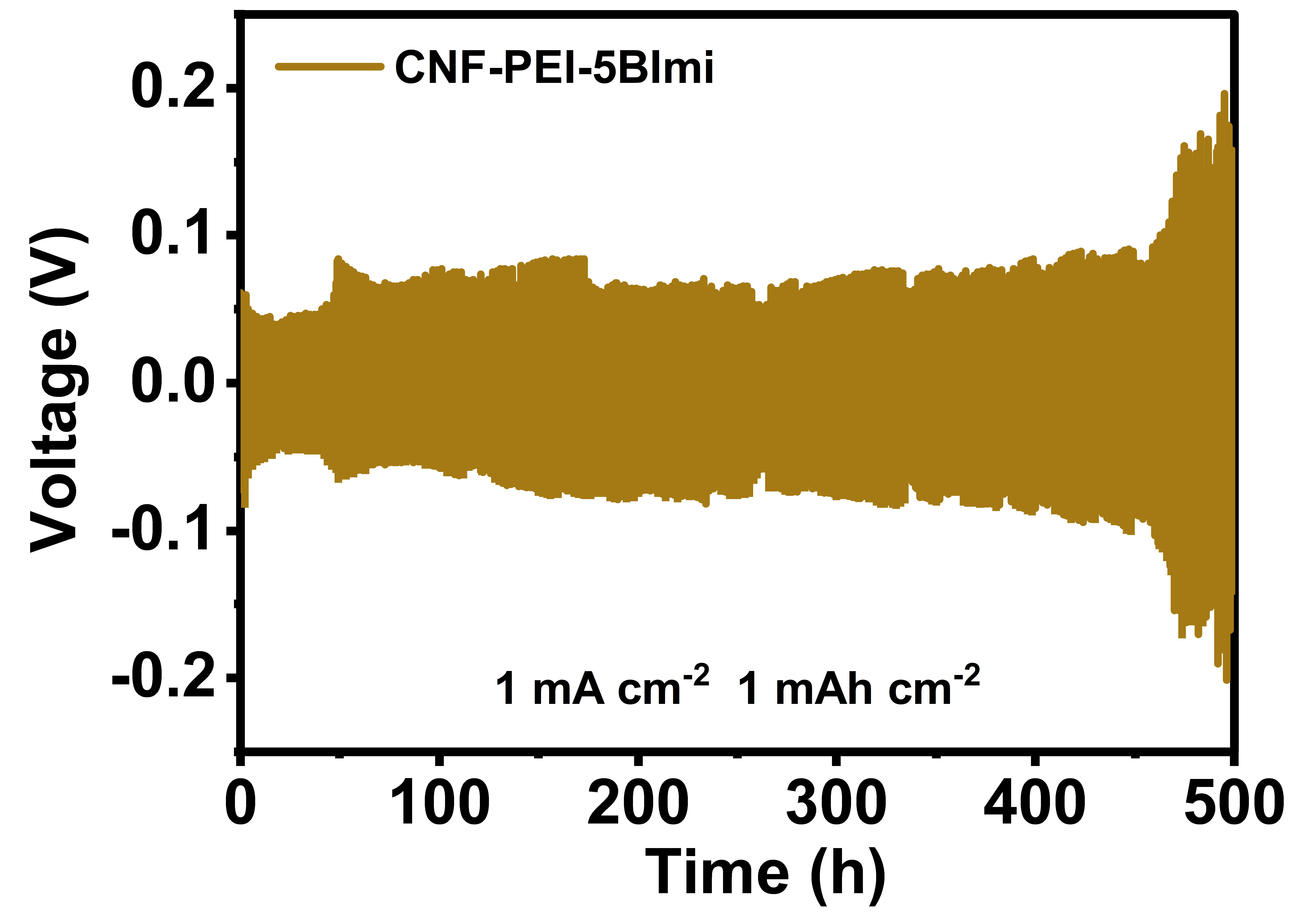


**Figure S18.** Cycling performance of Zn||Zn symmetric cells with the laminated CNF-PEI-5BImi separator at 1 mA cm^-2^ and 1 mAh cm^-2^.


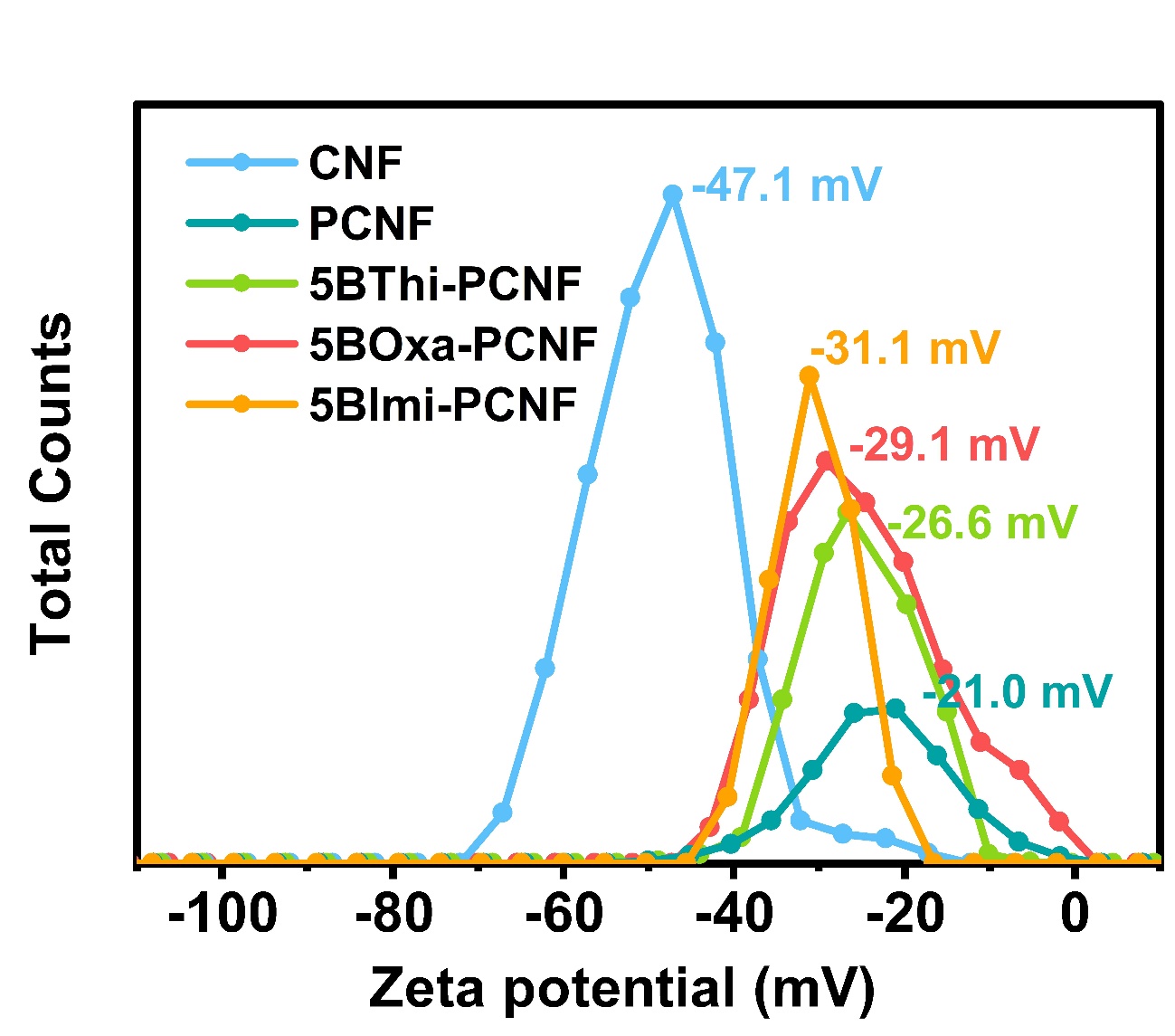


**Figure S19.** Zeta potentials of the CNF, PCNF, 5BThi-PCNF, 5BOxa-PCNF, and 5BImi-PCNF suspension.

As shown in Figure S19, the PCNF separator exhibits a considerable positive shift in zeta potential (-21.0 mV) compared to pristine CNF (-47.1 mV), which can be attributed to covalent amidation reactions between the amine groups of PEI and the carboxyl groups on the CNF surface. The incorporation of the electronegative imidazole-based molecule further reduces the zeta potential of PCNF to -31.1 mV for 5BImi-PCNF, which is still less negative than that of pristine CNF. This result confirms that imidazole grafting induces both steric and electronic modulations at the interface, thereby modifying the interfacial charge environment.





**Figure S20.** Surface potential mappings of the CNF, PCNF, and 5BImi-PCNF separators.

As illustrated in Figure S20, CNF, PCNF and 5Bim-PCNF separators all exhibit uniform surface potential at the macroscopic scale, and no obvious through‑thickness potential gradient was observed. The measured surface potentials were -272 mV for CNF, -64 mV for PCNF, and -197 mV for 5BImi‑PCNF, respectively. This systematic potential evolution verifies that the enhanced performance of the separators stems from nanoscale interfacial electronic modulation instead of a macroscopic bulk electrostatic gradient.


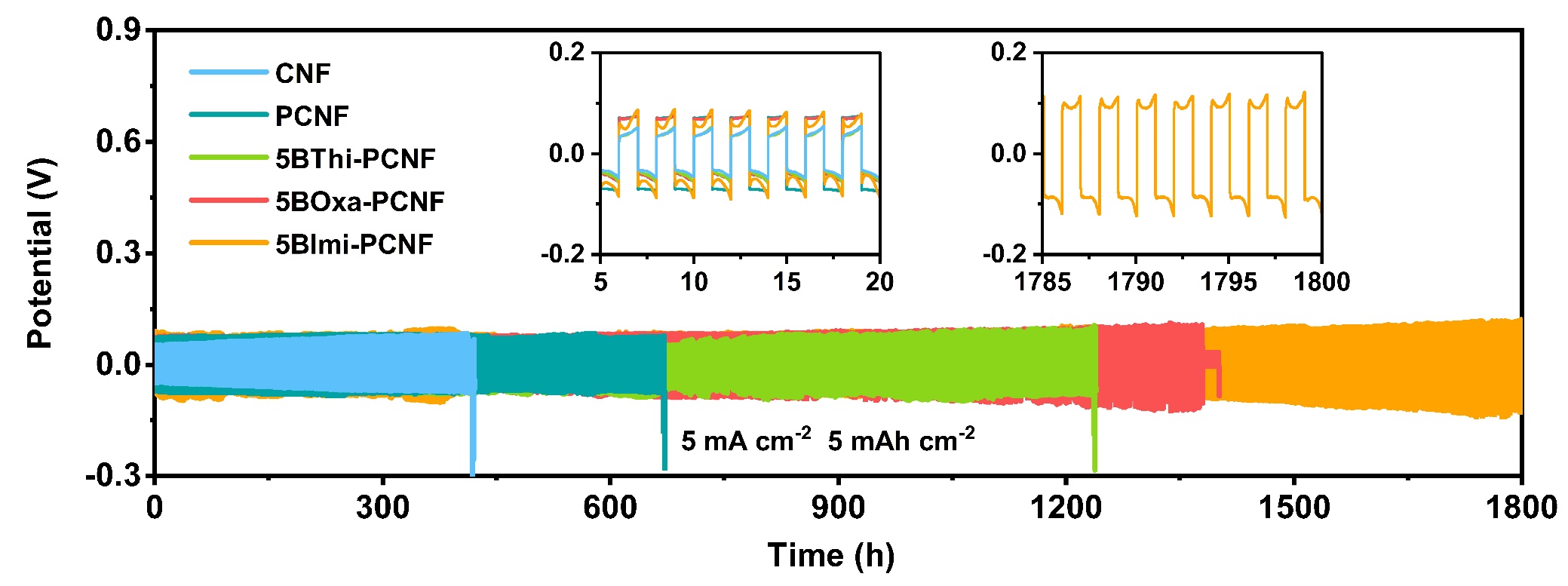


**Figure S21.** The cycling performance of Zn||Zn symmetric cells with different separators at 5 mA cm^-2^ and 5 mAh cm^-2^.

As shown in Figure S21, owing to highly reversible Zn plating/stripping behavior enabled by 5BImi-PCNF separator, stable operation for over 1800 h is achieved at 5 mA cm^-2^ and 5 mAh cm^-2^. In contrast, Zn||Zn cells using CNF, PCNF, 5BThi-PCNF, and 5BOxa-PCNF separators encounter failure after 420, 670, 1240, and 1390 h, respectively.


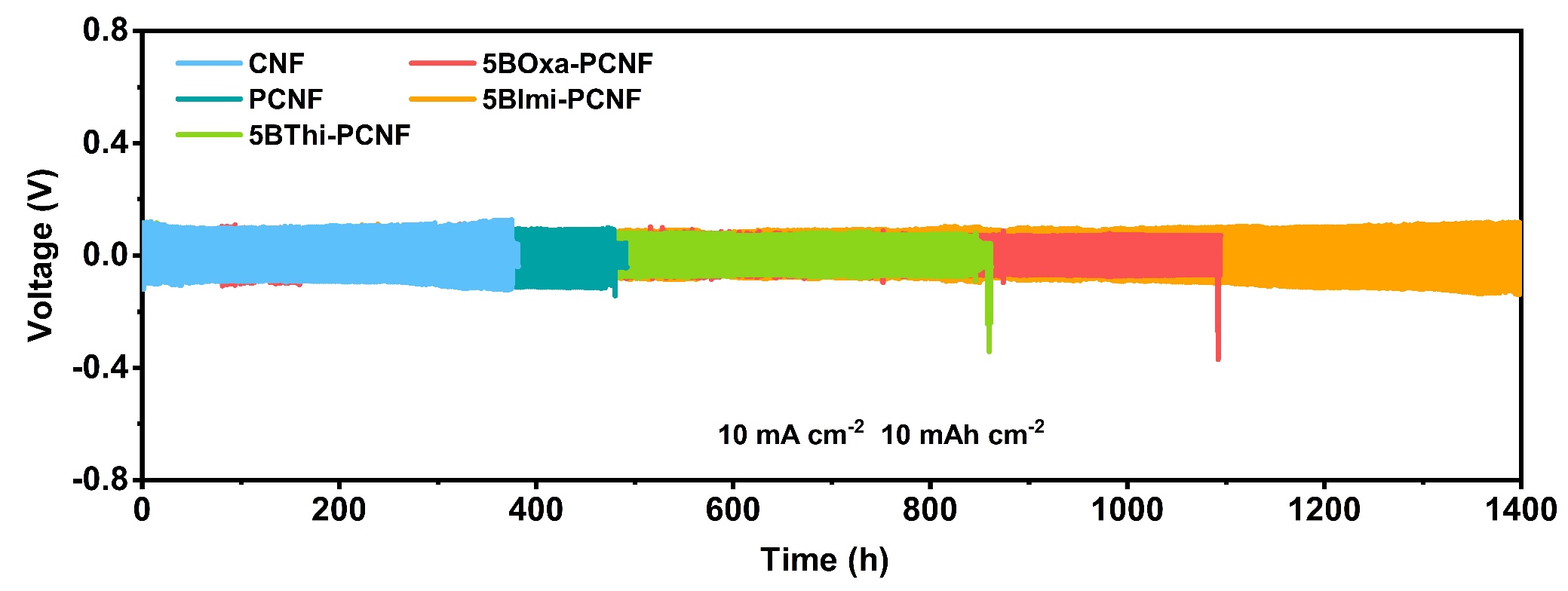


**Figure S22.** The cycling performance of Zn||Zn symmetric cells with different separators at 10 mA cm^-2^ and 10 mAh cm^-2^.





**Figure S23.** Voltage-time profiles of Zn||Zn cells with the CNF, PCNF, 5BThi-PCNF, 5BOxa-PCNF and 5BImi-PCNF separators at (a) 10 mA cm^-2^, 10 mAh cm^-2^ and (c) 20 mA cm^-2^, 20 mAh cm^-2^. The correspinding cycling lifetimes of Zn||Zn cells with the CNF and 5BImi-PCNF separators at (b) 10 mA cm^-2^, 10 mAh cm^-2^ and (d) 20 mA cm^-2^, 20 mAh cm^-2^.

**
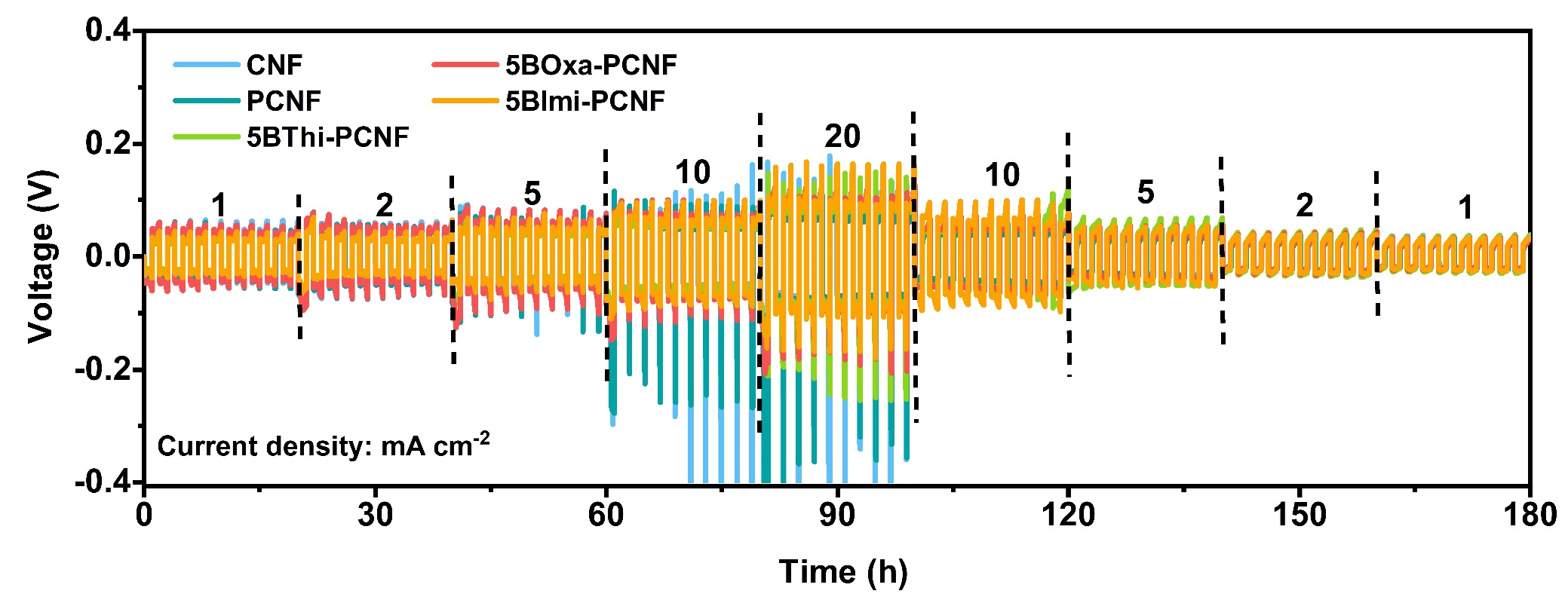
**

**Figure S24.** Rate performance of the Zn||Zn cells with the CNF, PCNF, 5BThi-PCNF, 5BOxa-PCNF, and 5BImi-PCNF separators.


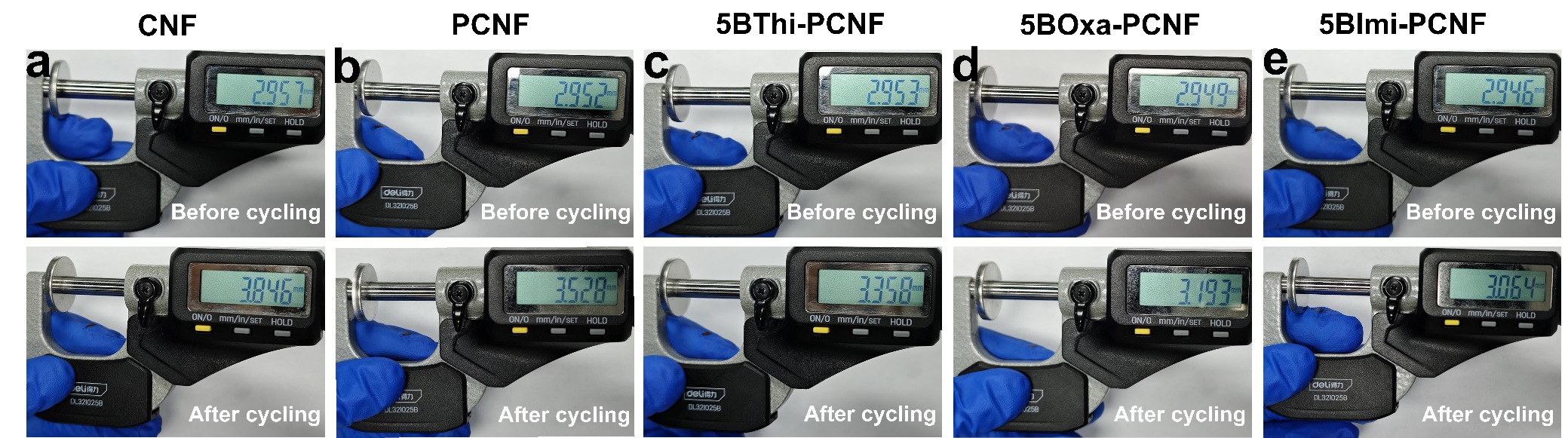


**Figure S25.** Digital images showing the thickness variations of Zn||Zn cells after cycling with (a) CNF, (b) PCNF, (c) 5BThi-PCNF, (d) 5BOxa-PCNF, and (e) 5BImi-PCNF separators.

The thickness variation of the cycled Zn||Zn cells, a reliable indicator of HER intensity, is found to be highly dependent on the type of separator employed. As shown in Figure S25, cells using CNF, PCNF, 5BThi-PCNF, and 5BOxa-PCNF separators exhibit progressive thickness increases of 30.1%, 19.5%, 13.7%, and 8.3%, respectively, reflecting varying degrees of HER severity. These significant changes in thickness provide evidence for the extent of parasitic gas evolution occurring at the Zn anode surface during the cycling process. Strikingly, the implementation of the 5BImi-PCNF separator leads to a significant reduction of the thickness variation to only 4.1%, indicating a substantial suppression of the HER.

**
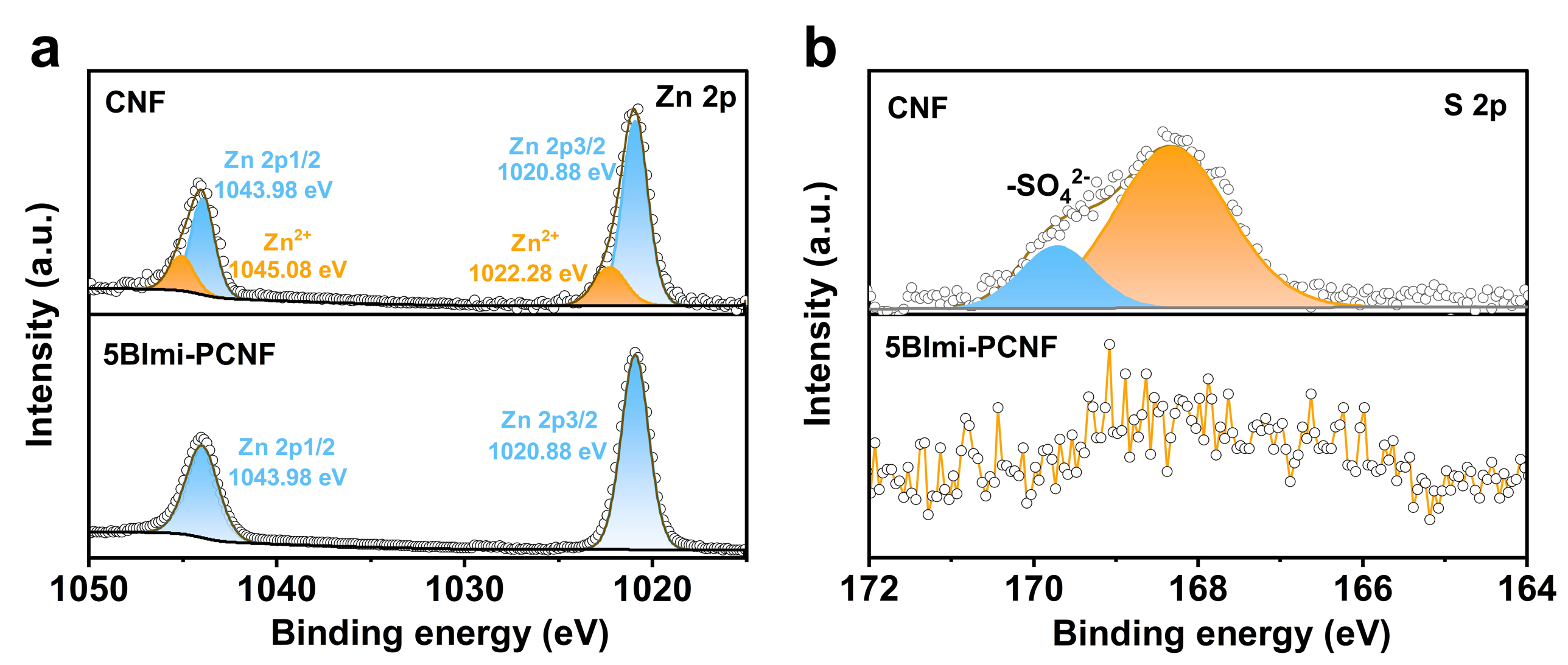
**

**Figure S26.** (a) Zn 2p and (b) S 2p XPS spectra of the Zn anode with CNF and 5BImi-PCNF after cycling.

As shown in Figure S26, the Zn 2p spectra of Zn anodes with CNF and 5BImi-PCNF separators show the characteristic signals of metallic Zn at 1020.88 eV (Zn 2p_3/2_) and 1043.98 eV (Zn 2p_1/2_). Notably, the Zn anode cycled with the CNF separator displays two additional peaks at ~1022.28 and ~1045.08 eV, which can be associated with Zn_4_SO_4_(OH)_6_·5H_2_O (ZSH) byproducts. In contrast, these ZSH-realted features are absent in the Zn anode cycled with the 5BImi-PCNF separator. This is further supported by the S 2p spectra, where sulfate-related peaks (168.8 and 169.8 eV) are detected only in the Zn anode cycled with the CNF separator. These findings confirm that the 5BImi-PCNF separator effectively prevents the formation of sulfate byproducts on the Zn anode surface.


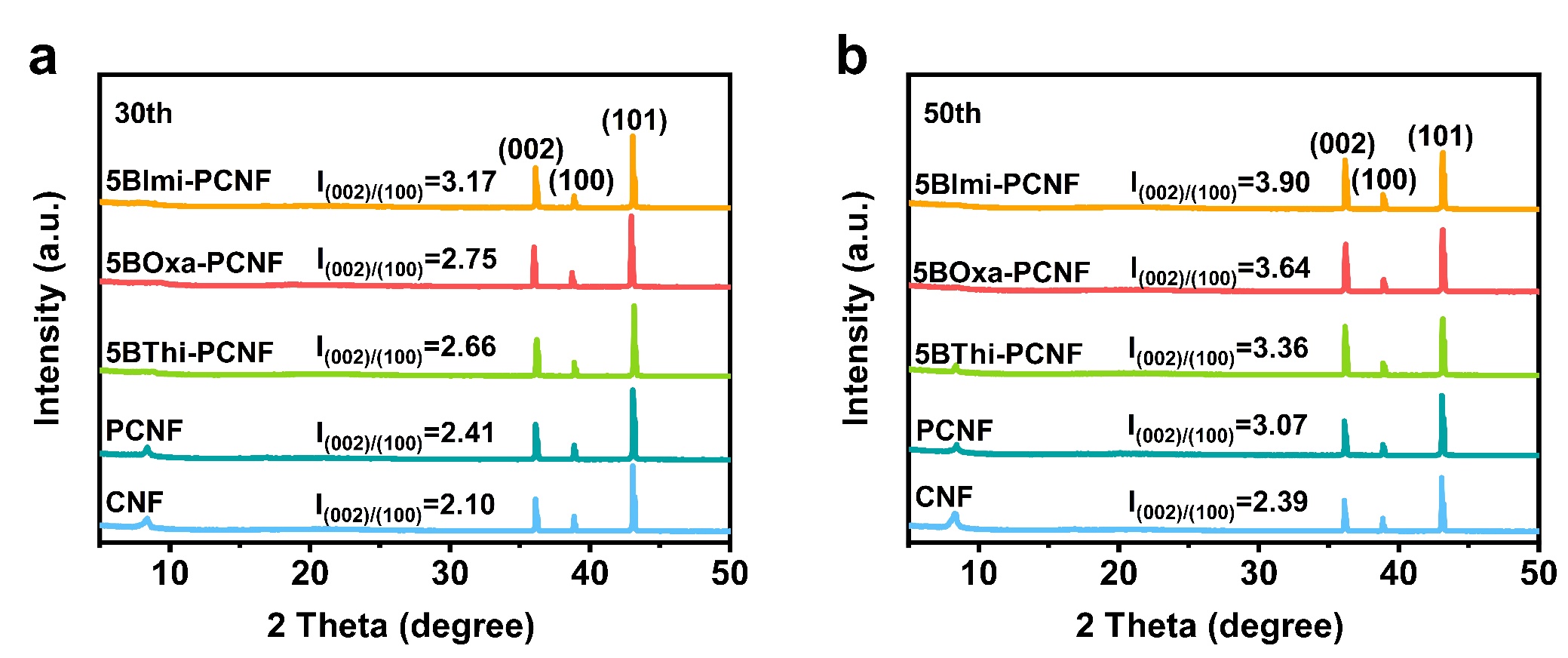


**Figure S27.** XRD patterns of the Zn electrode surfaces from Zn||Zn cells with CNF, PCNF, 5BThi-PCNF, 5BOxa-PCNF, and 5BImi-PCNF separators after (a) 30 cycles and (b) 50 cycles.

As illustrated in Figure S27a, after 30 cycles, the Zn||Zn cell with the 5BImi-PCNF separator exhibits the highest I_(002)/(100)_ intensity ratio of 3.17, compared to CNF (2.10), PCNF (2.41), 5BThi-PCNF (2.66), and 5BOxa-PCNF (2.75). A similar trend is observed after 50 cycles, as shown in Figure S27b, where the I_(002)/(100)_ ratio further increases to 3.90 for 5BImi-PCNF, remaining higher than that of CNF (2.39), PCNF (3.07), 5BThi-PCNF (3.36), and 5BOxa-PCNF (3.64). These results demonstrate that the 5BImi-PCNF separator effectively promotes Zn deposition along the (002) crystal plane, which is favorable for uniform and stable Zn growth.


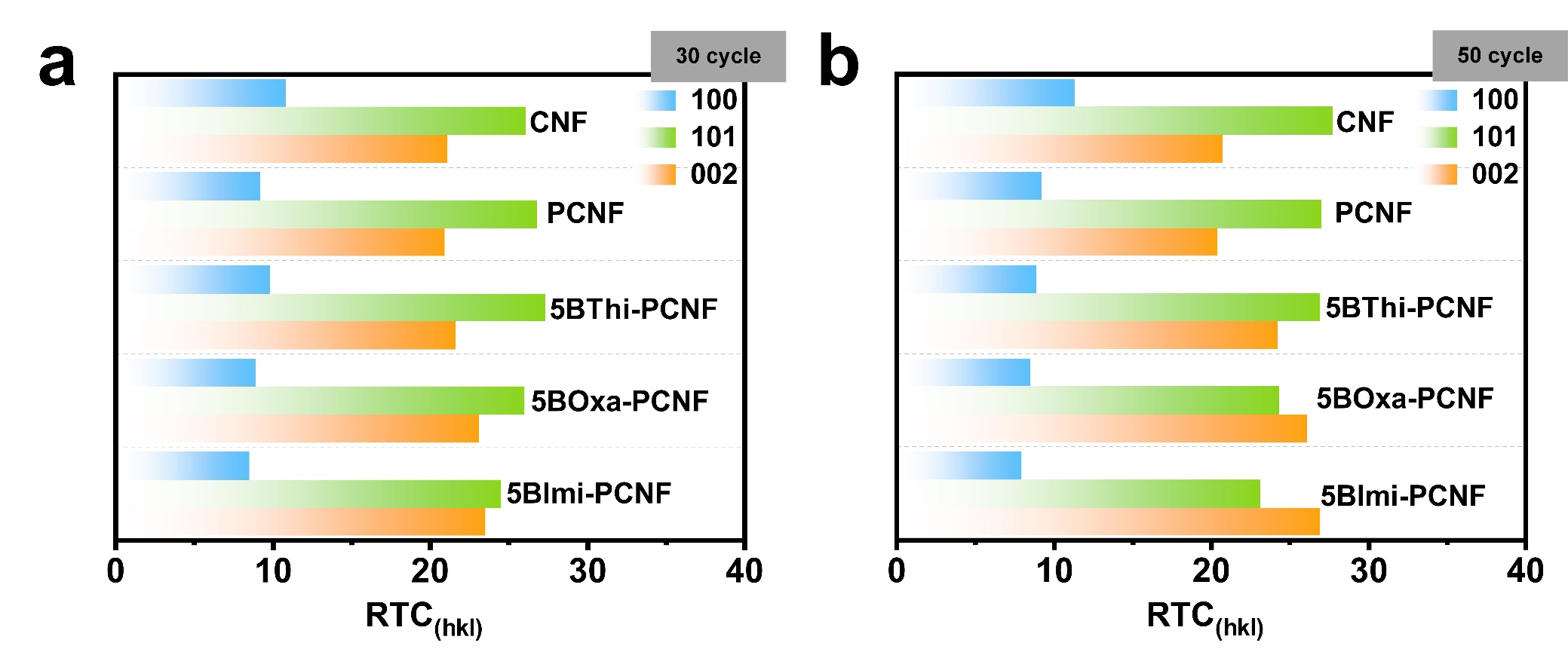


**Figure S28.** The RTC value of deposited Zn anodes with CNF, PCNF, 5BThi-PCNF, 5BOxa-PCNF, and 5BImi-PCNF separators after (a) 30 cycles and (b) 50 cycles.

As illustrated in Figure S28, the RTC values of the Zn (002) crystallographic plane progressively increase throughout the cycling process with the 5BImi-PCNF separator, indicating a gradually enhanced preferential deposition of Zn along this plane. In contrast, the RTC of the Zn (100) plane sharply declines under the same conditions, reflecting a clear suppression of Zn deposition on this less favorable orientation. Comparative analysis across various separators, including CNF, PCNF, 5BThi-PCNF, and 5BOxa-PCNF, reveals that the 5BImi-PCNF separator consistently yields markedly higher RTC values for the (002) plane compared to all other tested materials. This pronounced disparity underscores the unique electrochemical ability of the 5BImi-PCNF separator to direct Zn deposition toward the more favorable (002) crystallographic plane.


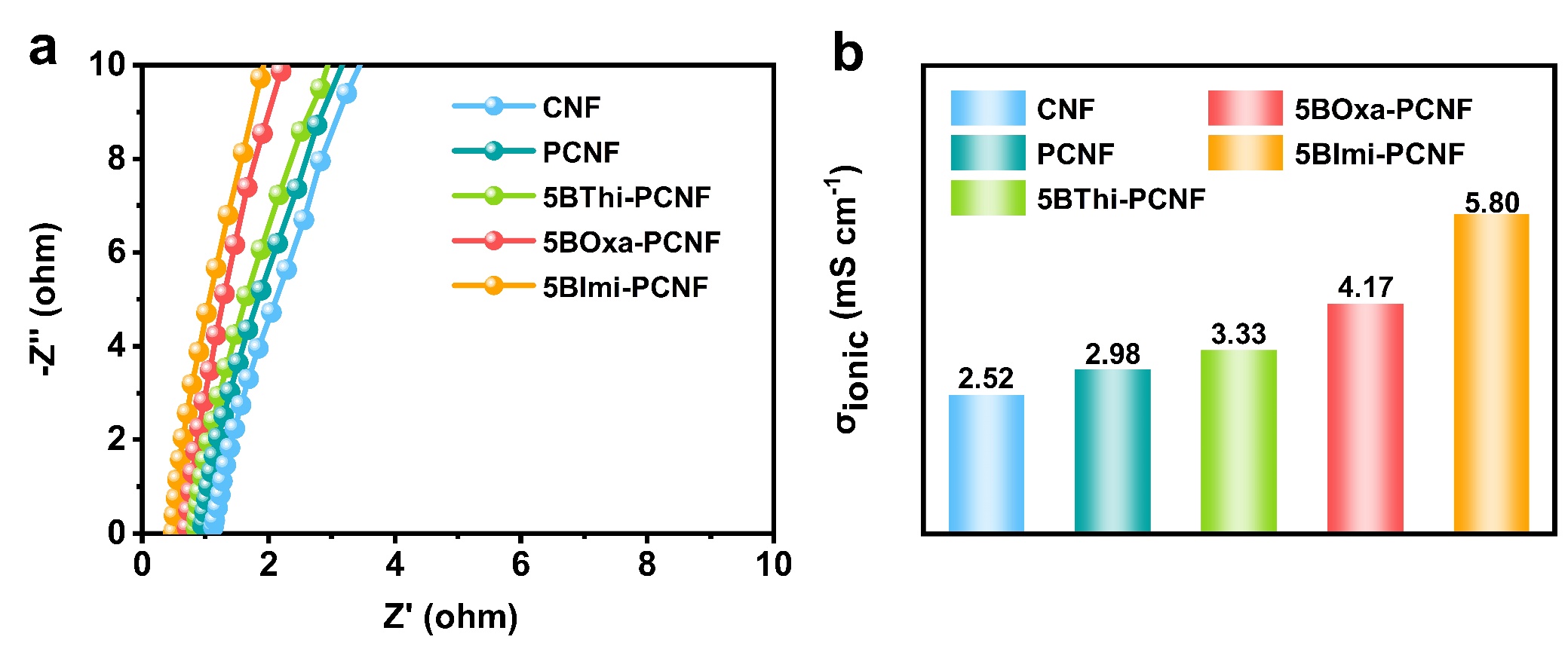


**Figure S29.** (a) EIS and (b) the ionic conductivity of CNF, PCNF, 5BThi-PCNF, 5BOxa-PCNF, and 5BImi-PCNF separators.

As shown in Figure S29, the ionic conductivity of the 5BImi-PCNF separator reaches 5.80 mS cm^-1^, representing a substantial improvement over the CNF (2.52 mS cm^-1^), PCNF (2.98 mS cm^-1^), 5BThi-PCNF (3.33 mS cm^-1^), and 5BOxa-PCNF (4.17 mS cm^-1^) separators. These results indicate that the 5BImi-PCNF separator facilitates faster ion transport, contributing to its enhanced performance.

**
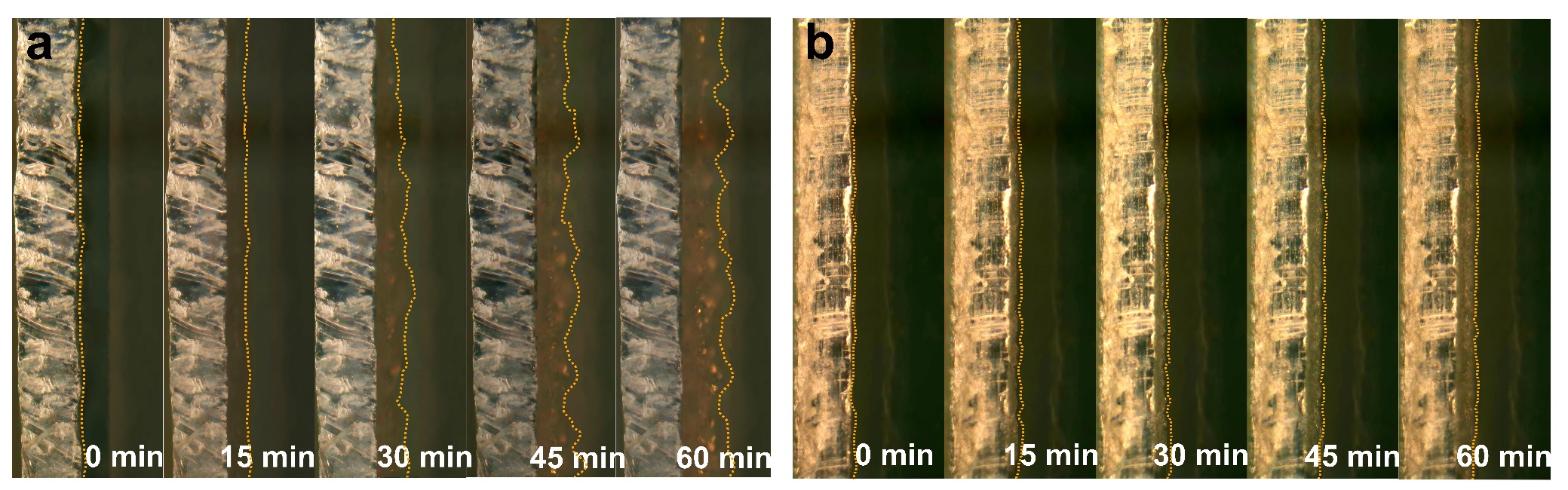
**

**Figure S30.** In-situ optical observation of the Zn plating behavior with (a) CNF and (b) 5BImi-PCNF separators.

In situ optical microscopy was employed to examine the difference in electrochemical behavior at 1 mA cm^-2^ and 1 mAh cm^-2^. The utilization of the CNF separator leads to the rapid formation of rough and irregular Zn protrusions after only 15 min of electroplating, which display uncontrolled growth at the electrode-electrolyte interface (Figure S30a). In contrast, the implementation of the 5BImi-PCNF separator promotes uniform Zn deposition, preserving a dense, smooth, and dendrite-free morphology throughout the complete plating process (Figure S30b). This distinct contrast emphasizes the enhanced performance of the 5BImi-PCNF separator in regulating the Zn^2+^ ion flux, homogenizing the local electric field distribution, and effectively suppressing dendritic growth.





**Figure S31**. FTIR spectra of (a) 5BThi-PCNF, (b) 5BOxa-PCNF, and (c) 5BImi-PCNF separators after soaking.


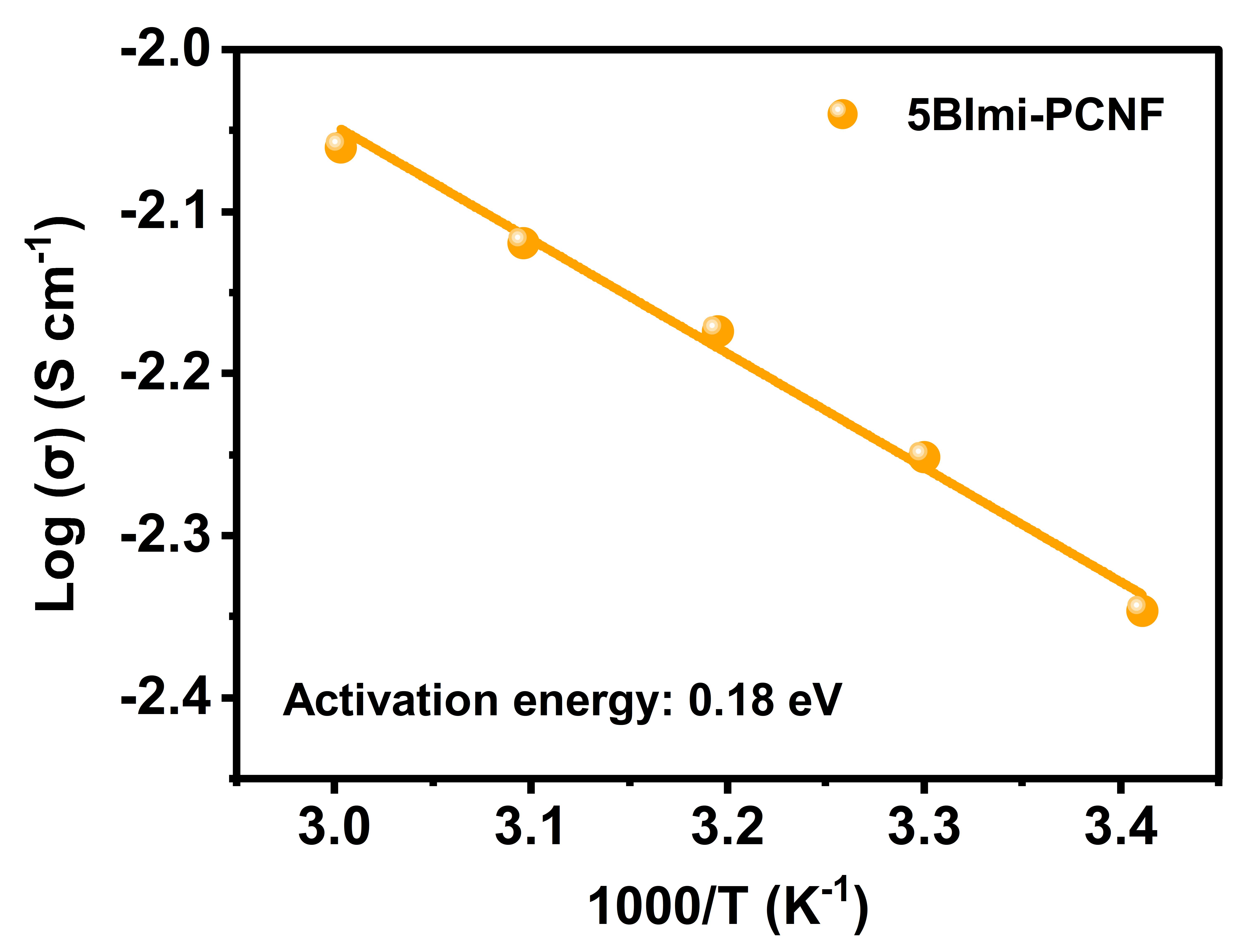


**Figure S32**. Temperature-dependent ionic conductivity measured in 2 M ZnSO_4_ with activation energy labels.





**Figure S33.** (a) FTIR, (b) high-resolution C 1s XPS and (c) high-resolution N 1s spectra of the 5BImi-PCNF separator after cycling in Zn||Zn cells.

As shown in Figure S33, the cycled 5BImi-PCNF separator retains the characteristic FTIR bands at 1646 cm^-1^ (HN-C=O), 1242 cm^-1^ (C-N) and 1026 cm^-1^ (C-O-C), whereas the C=N stretching band redshifted from 1542 to 1521 cm^-1^. In the high-resolution C 1s XPS spectra, the peaks assigned to C=O-N, O-C=O and C-N remain essentially unchanged, while the C=N-related peak shifts from 285.8 to 286.1 eV. A new Zn-N peak is observed in the N 1s spectrum, indicating coordination between Zn^2+^ ions and the imidazole nitrogen atoms.


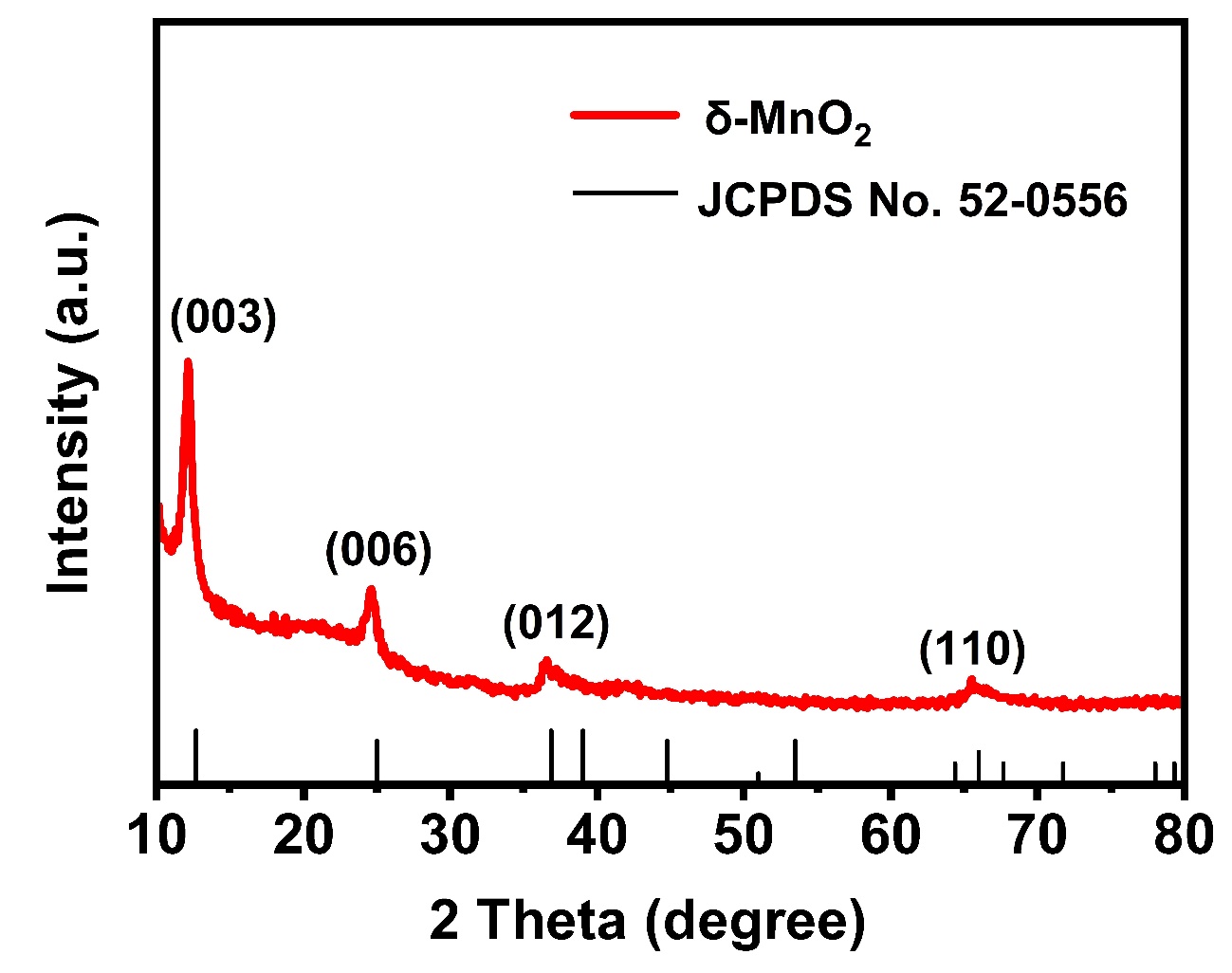


**Figure S34.** XRD pattern of the as-fabricated δ-MnO_2_ sample.

As shown in Figure S34, all diffraction peaks are well indexed to the rhombohedral structure of K_0.27_MnO_2_·0.54H_2_O (JCPDS No. 52-0556), which consists of loosely stacked layers of edge-sharing MnO_6_ octahedra with an interlayer spacing of approximately 0.74 nm, where K^+^ ions reside between the layers.


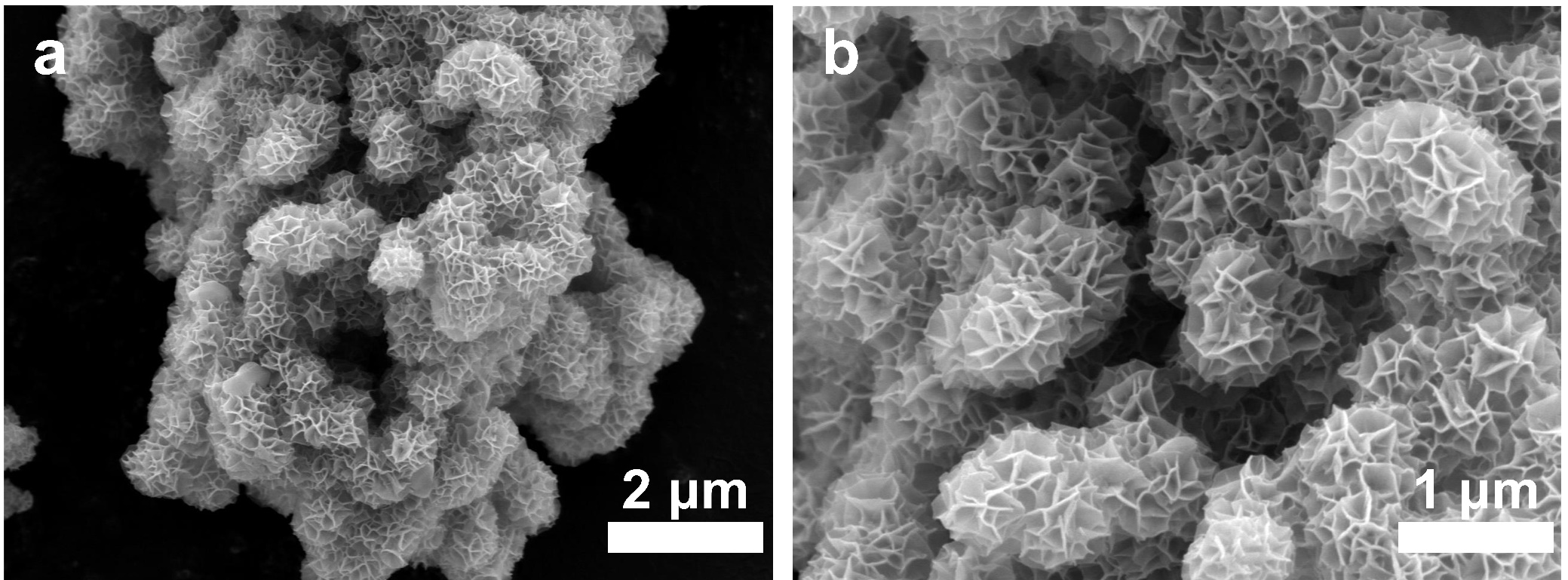


**Figure S35.** SEM images of δ-MnO_2_ at varying magnifications.

As shown in Figure S35, the δ-MnO_2_ features a uniform flower-like architecture composed of interconnected microscale flakes. These flakes are radially arranged and densely packed, forming a highly porous structure with a large surface area. This effectively promotes electrolyte penetration and facilitates efficient ion transport, which are essential for enhanced electrochemical performance.


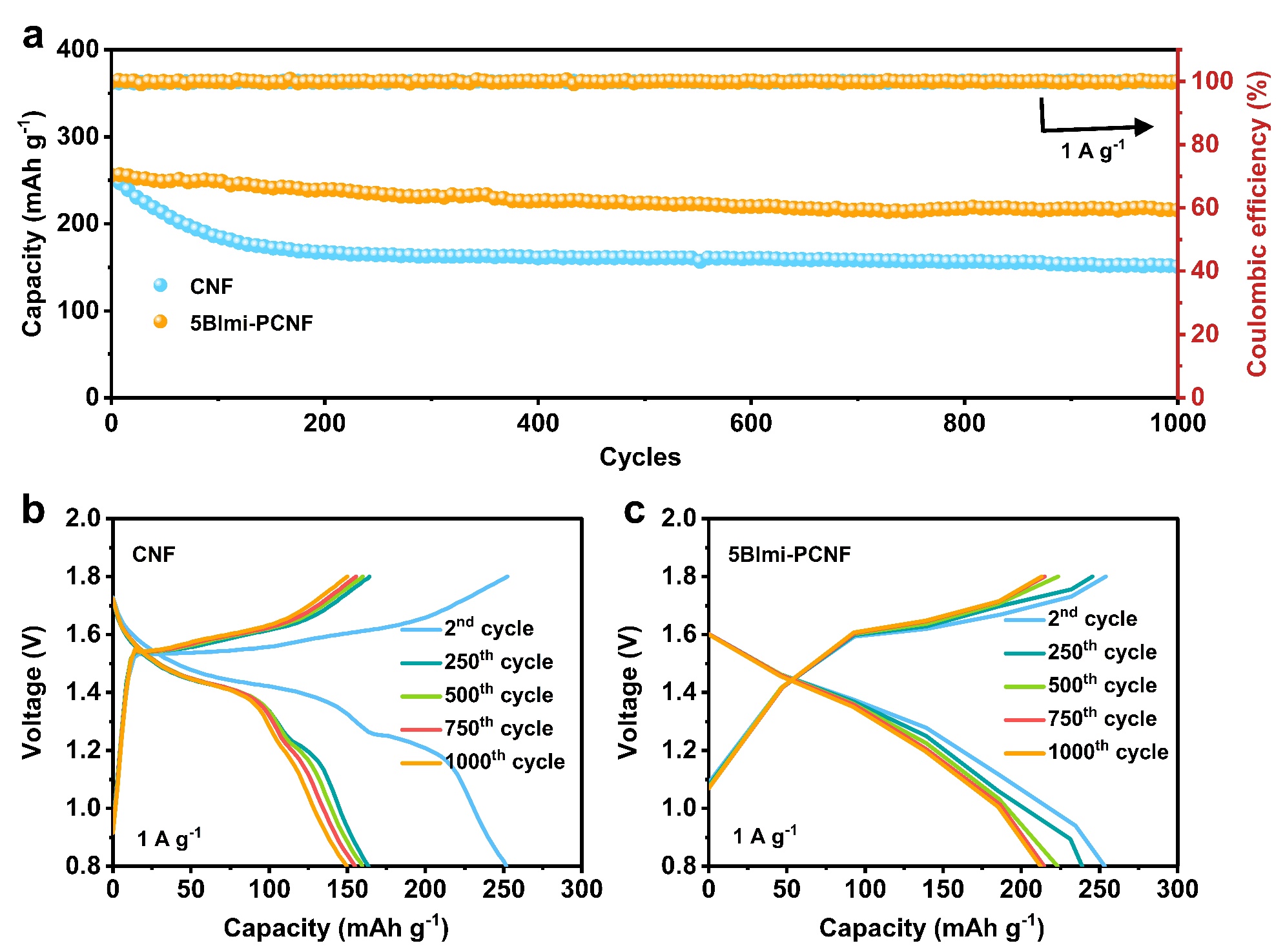


**Figure S36.** (a) Cycling performance of the Zn||MnO_2_ full cells with CNF and 5BImi-PCNF separators at 1 A g^-1^. Discharge-charge profiles of the Zn||MnO_2_ full cells with the (b) CNF and (c) 5BImi-PCNF separators at different cycles.

As illustrated in Figure S36, the capacity of Zn||MnO_2_ full cell with CNF separator sharply decreases to 59.2 % after 1000 cycles at 1 A g^-1^. In contrast, the Zn||MnO_2_ full cell with 5BImi-PCNF separator retains 84.2 % of its initial capacity after 1000 cycles, highlighting the great potential of the 5BImi-PCNF separator in the realm of high-performance AZIBs.

**
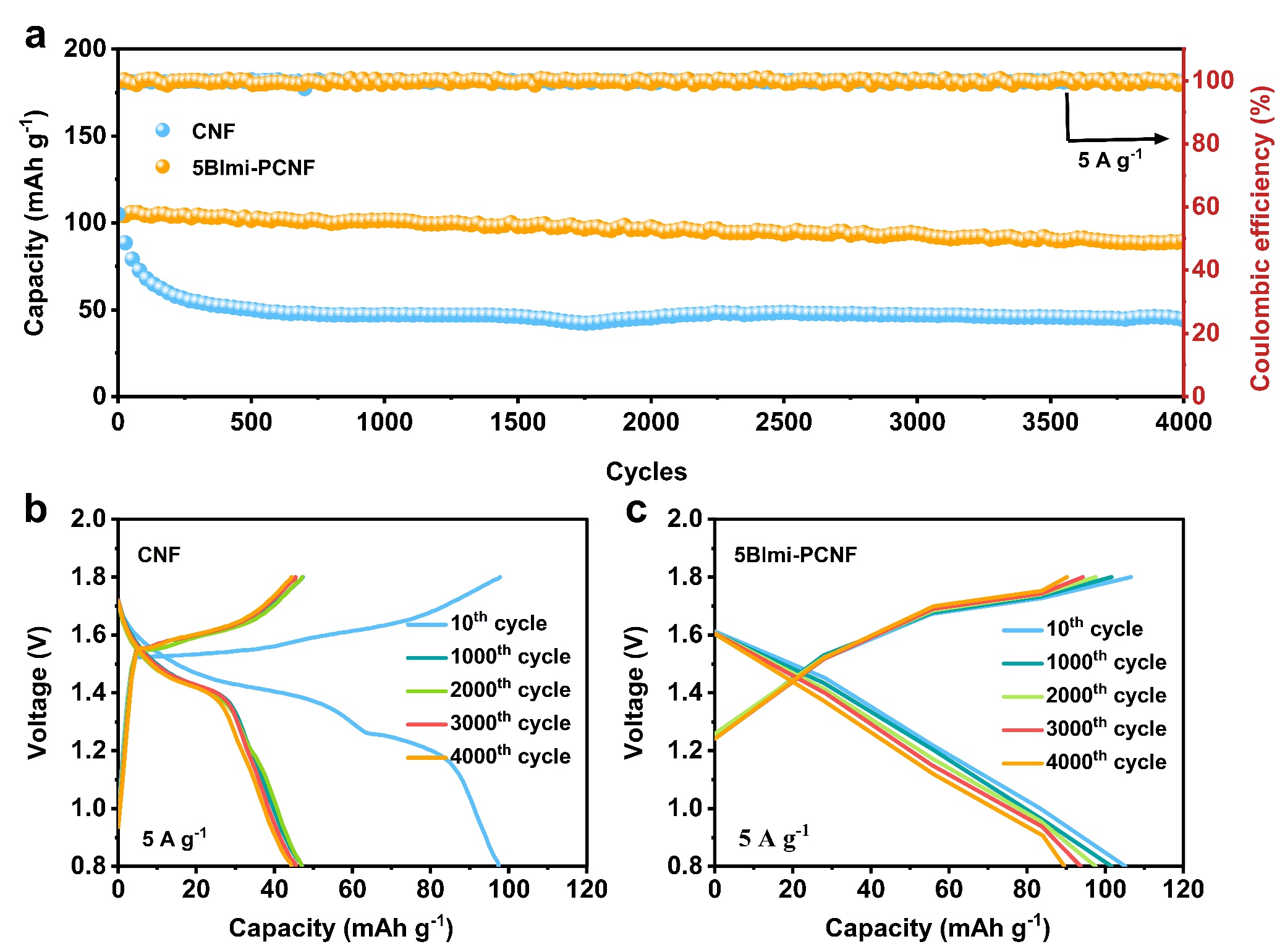
**

**Figure S37.** (a) Cycling performance of the Zn||MnO_2_ full cells with CNF and 5BImi-PCNF separators at 5 A g^-1^. Discharge-charge profiles of the Zn||MnO_2_ full cells with the (b) CNF and (c) 5BImi-PCNF separators at different cycles.

As illustrated in Figure S37, the Zn||MnO_2_ cell with the 5BImi-PCNF separator delivers a stable capacity of 105.4 mAh g^-1^ after 4000 cycles at a current density of 5 A g^-1^, corresponding to a high capacity retention of 86.5%. However, the Zn||MnO_2_ cell using the CNF separator suffers from severe capacity degradation, retaining only 44.4 mAh g^-1^ after the same number of cycles. This result highlights the superior cycling stability imparted by the 5BImi-PCNF separator.


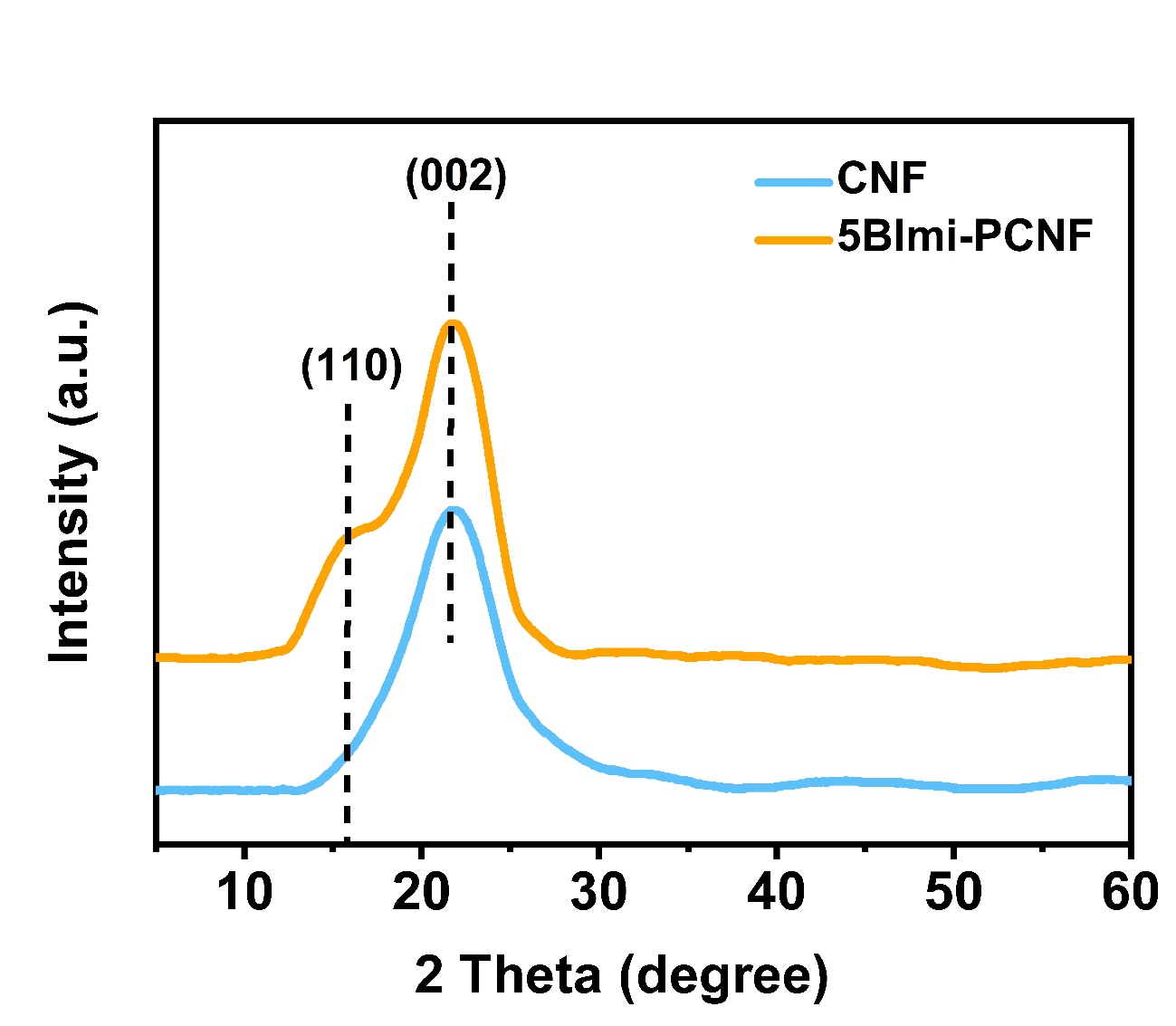


**Figure S38.** XRD patterns of the CNF and 5BImi-PCNF separators after cycling in Zn||MnO_2_ full cells.

As shown in Figure S38, the diffraction peak at 16.2°, assigned to the (110) plane of CNF, disappears after cycling with the CNF separator, mainly due to hydrogen bond rearrangement that disrupts the semi-crystalline structure. In contrast, the 5BImi-PCNF separator retains strong peaks at both 16.2° and 22.6° after cycling, demonstrating superior structural stability of the CNF framework within the composite.


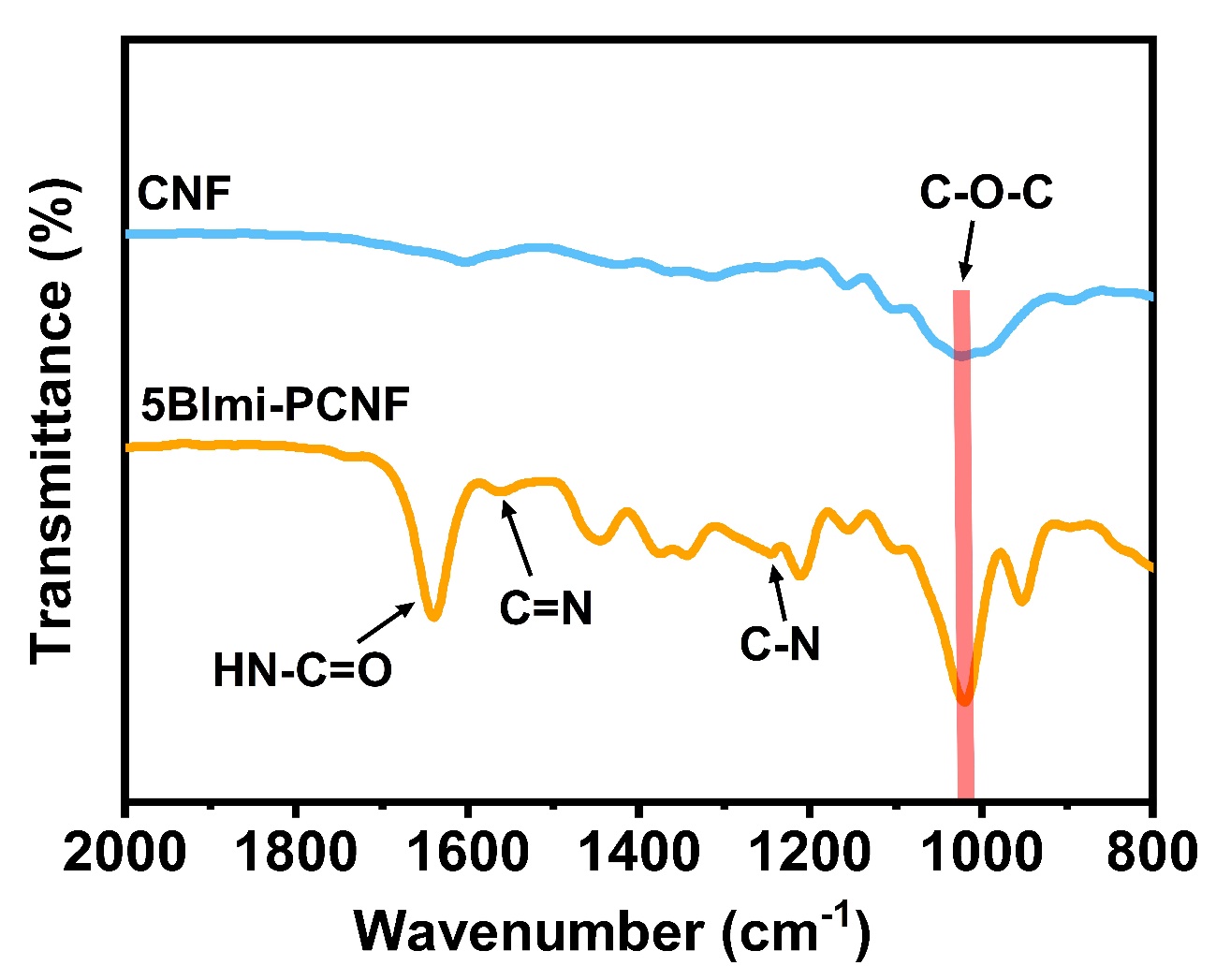


**Figure S39.** FTIR spectra of the CNF and 5BImi-PCNF separators after cycling in Zn||MnO_2_ full cells.

As shown in Figure S39, the C-O-C stretching vibration peak of the CNF separator shifts from 1024 cm^-1^ to 1011 cm^-1^ after cycling, accompanied by significant broadening and a notable decrease in peak intensity. These spectral changes suggest a disruption of the semi-crystalline structure, primarily caused by the hydrogen bond rearrangement during repeated cycling. In contrast, the 5BImi-PCNF separator maintains sharp and intense C-O-C peaks, indicating that its composite architecture effectively stabilizes the hydrogen bonding network and preserves structural integrity.


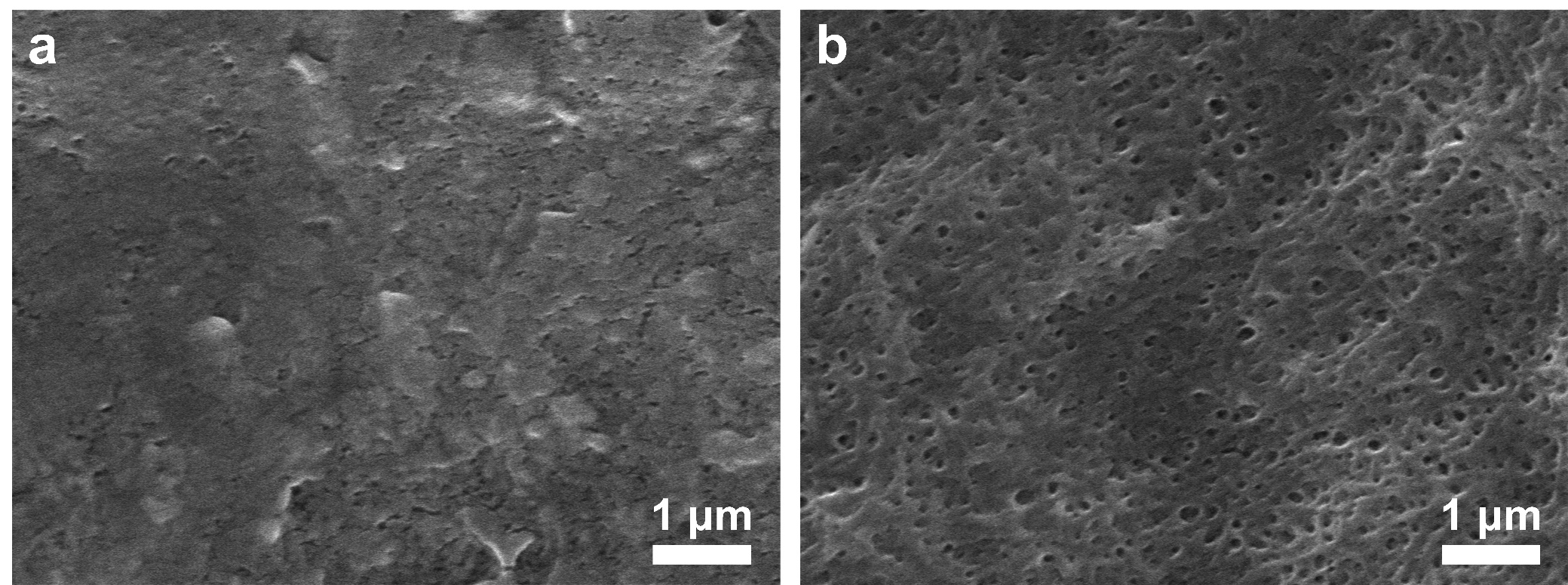


**Figure S40.** SEM images of the (a) CNF and (b) 5BImi-PCNF separators after cycling in Zn||MnO_2_ full cells.

As illustrated in Figure S40, the CNF separator undergoes substantial structural degradation following electrochemical cycling, with no identifiable porous architecture remaining. This degradation can be primarily attributed to hydrogen bond rearrangement, which compromises the structural integrity of the CNF network and ultimately leads to the collapse of its internal pore system. In stark contrast, the 5BImi-PCNF separator retains its original nanoporous structure even after prolonged electrochemical cycling, demonstrating the capability of the composite design to effectively stabilize the CNF framework and prevent pore collapse under repeated operating conditions.


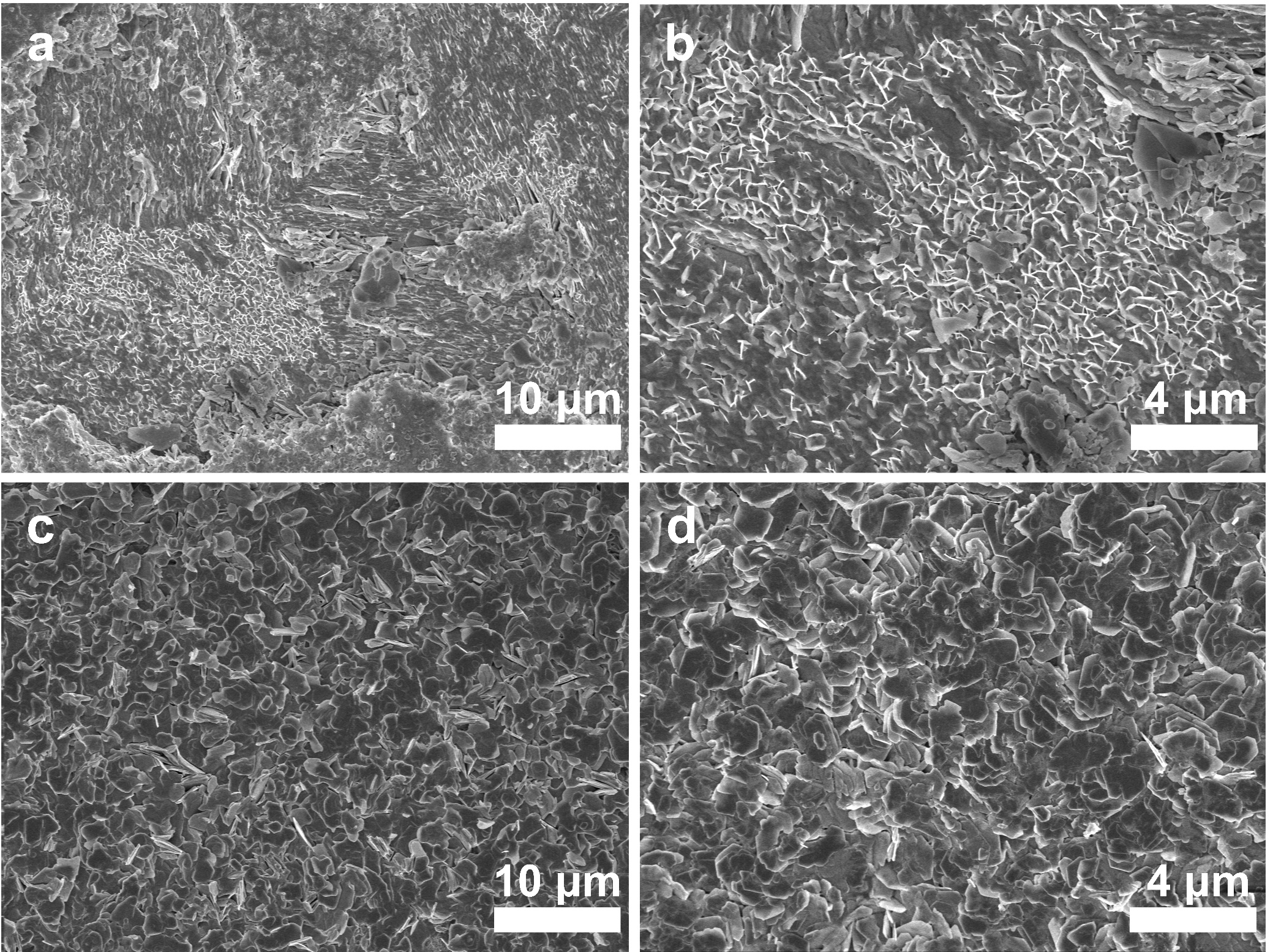


**Figure S41.** SEM images of the Zn anodes in Zn||MnO_2_ full cells assembled with the (a, b) CNF and (c, d) 5BImi-PCNF separators after cycling.

As depicted in Figure S41, the Zn electrode that undergoes cycling in Zn||MnO₂ full cells with the CNF separator presents a rough and inhomogeneous surface, which is covered with irregular, chunky byproducts. These byproducts result from unwanted side reactions between Zn and the aqueous electrolyte. Conversely, the cycled Zn electrode with the 5BImi-PCNF separator showcases a significantly smoother and more uniform surface, with no observable dendrite formation or byproduct accumulation. This finding evidently shows that the 5BImi-PCNF separator effectively inhibits dendrite growth and alleviates side reactions, thus enhancing interfacial stability during the cycling process.


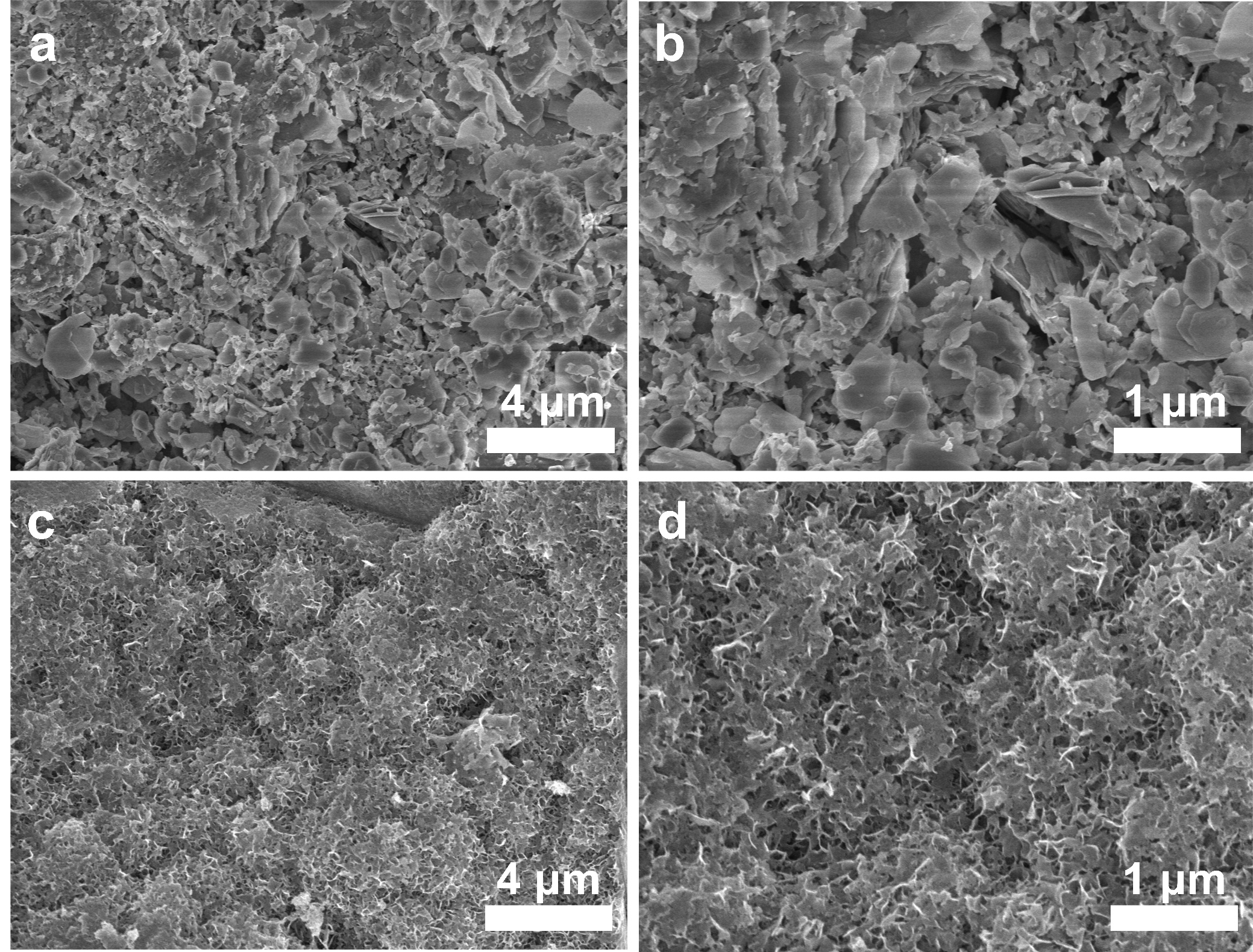


**Figure S42.** SEM images of the MnO_2_ cathodes in Zn||MnO_2_ full cells assembled with the (a, b) CNF and (c, d) 5BImi-PCNF separators after cycling.

As shown in Figure S42, the MnO_2_ cathode in the Zn||MnO_2_ full cell with the CNF separator undergoes noticeable structural degradation after cycling. This degradation is primarily attributed to the repeated insertion and extraction of H^+^ and Zn^2+^ ions within the layered δ-MnO_2_ framework, leading to dissolution and severe distortion of its characteristic flower-like morphology. In contrast, the MnO_2_ cathode in the cell employing the 5BImi-PCNF separator largely preserves its original flower-like architecture, even after prolonged cycling, indicating enhanced structural integrity. This sharp difference underscores the crucial impact of separator design on the morphological stability and long-term durability of MnO_2_ cathodes in Zn||MnO_2_ full cells.

**
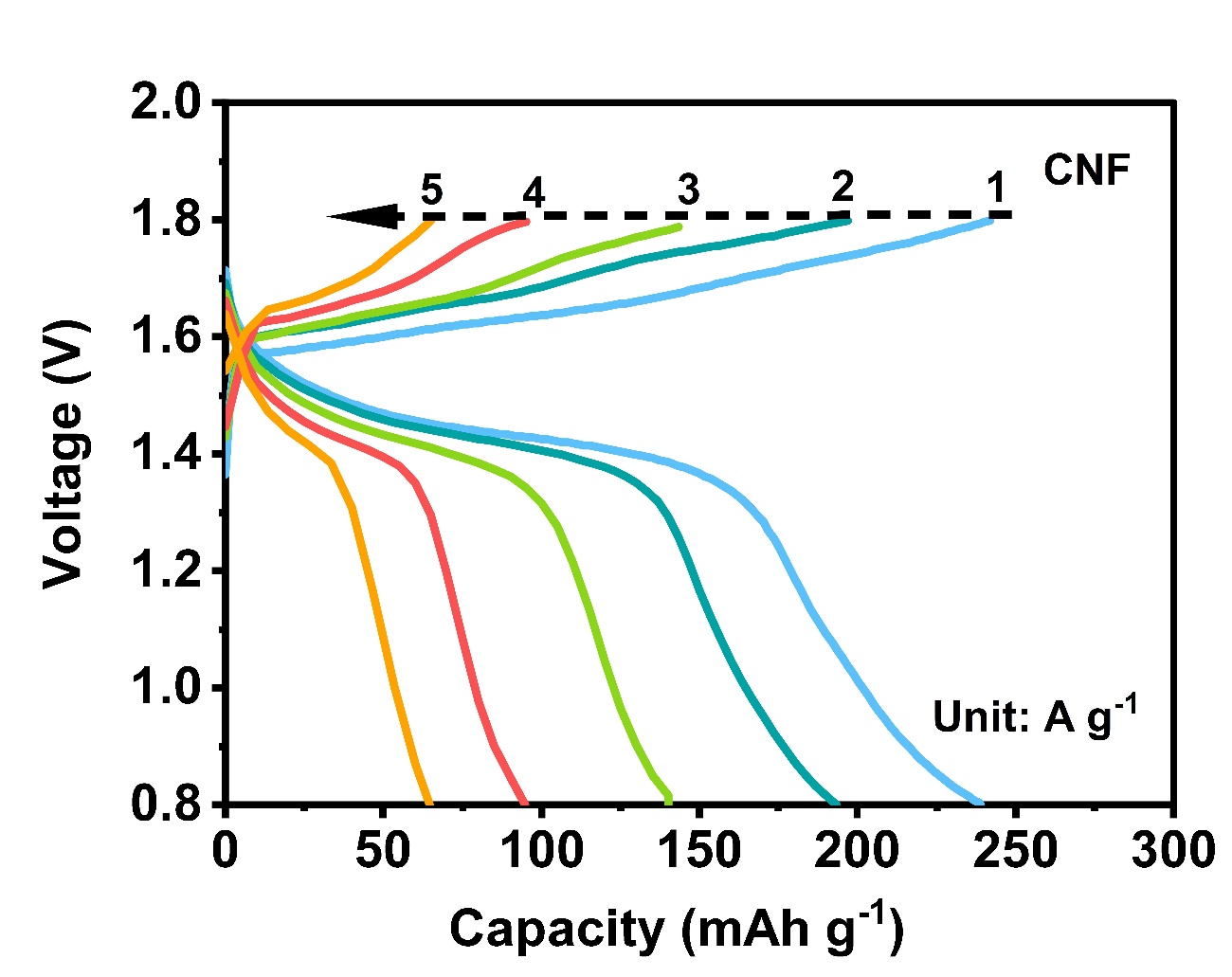
**

**Figure S43.** Discharge-charge profiles of Zn||MnO_2_ full cells with the CNF separator at different current densities.

As shown in Figure S43, Zn||MnO_2_ battery with the CNF separator delivers capacities of 239.1, 193.3, 140.2, 94.8, and 64.6 mAh g^-1^ at current densities of 1, 2, 3, 4, and 5 A g^-1^, respectively.

**
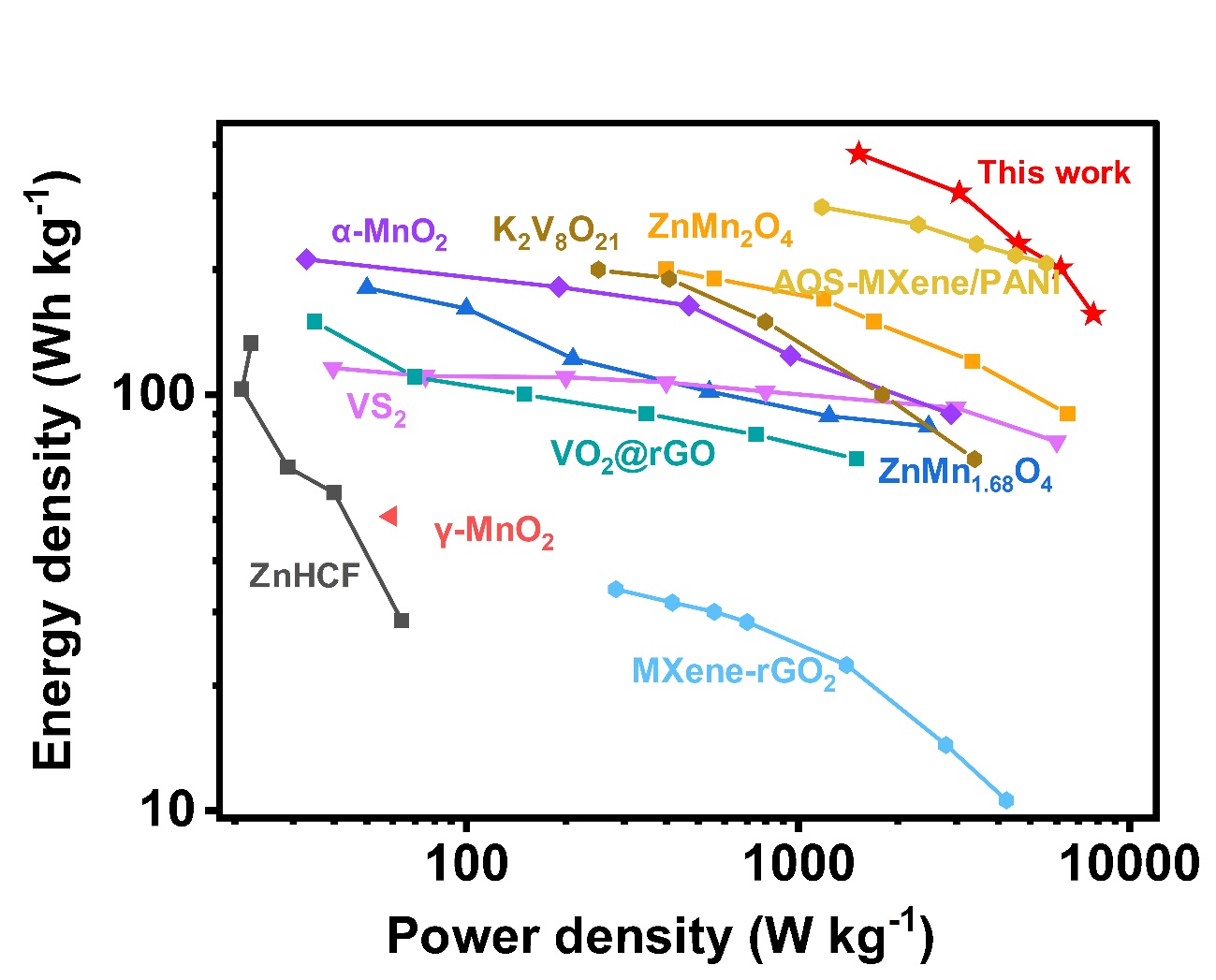
**

**Figure S44.** Comparative Ragone plot of Zn||MnO_2_ full cells utilizing the 5BImi-PCNF separator and other reported ZIB systems.

As shown in Figure S44, the Ragone plots of Zn||MnO_2_ full cells employing the 5BImi-PCNF separator outputs an energy density of 156 Wh kg^-1^ at the power density of 7.8 kW kg^-1^, surpassing previously reported for ZIBs using α-MnO_2_, γ-MnO_2_, ZnMn_2_O_4_, ZnHCF, ZnMn_1.68_O_4_, AQS-MXene/PANI, MXene-rGO_2_, K_2_V_8_O_21_, VO@rGO, and VS_2._^[21–30]^ This improved performance is attributed to the structurally stable architecture of the 5BImi-PCNF separator, which facilitates efficient ion transport while maintaining structural integrity under high-rate charge/discharge conditions. The results unequivocally demonstrate the viability of the 5BImi-PCNF separator for application in high-power Zn-ion batteries, providing significant advantages over state-of-the-art ZIB configurations.

**References**

[1] X. Shi, Z. Wang, S. Liu, Q. Xia, Y. Liu, W. Chen, H. Yu, K. Zhang, *Nat. Sustain.* **2024**, *7*, 315.

[2] H.-Y. Mi, X. Jing, Q. Zheng, L. Fang, H.-X. Huang, L.-S. Turng, S. Gong, *Nano Energy* **2018**, *48*, 327.

[3] J. Zhao, Y. Xu, S. Ma, Y. Wang, Z. Huang, H. Qu, H. Yao, Y. Zhang, G. Wu, L. Huang, W. Song, Z. Tang, X. Chen, *Adv. Mater.* **2022**, *34*, 2109254.

[4] R. Zhao, X. Dong, P. Liang, H. Li, T. Zhang, W. Zhou, B. Wang, Z. Yang, X. Wang, L. Wang, Z. Sun, F. Bu, Z. Zhao, W. Li, D. Zhao, D. Chao, *Adv. Mater.* **2023**, *35*, 2209288.

[5] Y. Wang, Z. Wang, W. K. Pang, W. Lie, J. A. Yuwono, G. Liang, S. Liu, A. M. D. Angelo, J. Deng, Y. Fan, K. Davey, B. Li, Z. Guo, *Nat. Commun.* **2023**, *14*, 2720.

[6] Z. Liu, R. Wang, Q. Ma, J. Wan, S. Zhang, L. Zhang, H. Li, Q. Luo, J. Wu, T. Zhou, J. Mao, L. Zhang, C. Zhang, Z. Guo, *Adv. Funct. Mater.* **2024**, *34*, 2214538.

[7] X. Shen, R. Zhang, P. Shi, X. Chen, Q. Zhang, *Adv. Energy Mater.* **2021**, *11*, 2003416.

[8] Z. Hong, Z. Ahmad, V. Viswanathan, *ACS Energy Lett.* **2020**, *5*, 2466.

[9] W. Yang, W. Yang, Y. Huang, Y. Wu, X. Ma, L. Dong, X. Peng, *Energy Storage Mater.* **2025**, *80*, 104436.

[10] Y. Chen, G. Zhou, X. Huang, Y. Liu, X. Tian, L. Wang, X. Liu, X. Ning, D. Zhu, Z. Bai, N. Wang, X. Ren, S. Dou, *Energy Storage Mater.* **2025**, *78*, 104247.

[11] C. Miao, D. Chen, W. Shen, Y. Zhang, T. Yao, L. Shen, W. Han, *Adv. Funct. Mater.* **2025**, e19971.

[12] Y. Dong, W. Fan, X. Wang, H. Huang, Y. Zhu, J. Chen, W. Tian, Y. Huang, J. Wu, *Adv. Funct. Mater.* **2026**, *36*, e13685.

[13] Y. Zhang, Y. Sun, K. Liu, Q. Xia, S. Zhao, Q. Ying, P. Chen, H. Lu, T. Shahid, X. Peng, Z. Ye, Y. Yang, *Adv. Funct. Mater.* **2025**, e20280.

[14] Q. Wang, X. Deng, X. Xue, J. Zhang, J. Zhao, Z. Sui, Y. Zou, L. Luo, W. Zhang, X. Liu, C. Lu, *Energy Environ. Sci.* **2025**, *18*, 5309.

[15] X. Li, J. Li, Q. Yang, J. Feng, X. Wang, L. Ye, Z. Liu, N. Jiang, Y. Duan, Z. Tao, S. Chen, F. Xu, J. Qiu, *Adv. Funct. Mater.* **2025**, e19947.

[16] R. Xue, Z. Wang, N. Yao, Y. Liu, H. Wang, M. Zhang, A. Shao, X. Tang, J. Liu, J. Tang, Z. Wang, Y. Ma, *Adv. Funct. Mater.* **2024**, *34*, 2400959.

[17] L. Cheng, W. Li, M. Li, S. Zhou, J. Yang, W. Ren, L. Chen, Y. Huang, S. Yu, J. Wei, *Adv. Funct. Mater.* **2024**, *34*, 2408863.

[18] X. Zhu, Z. Xu, T. Zhang, J. Zhang, Y. Guo, M. Shan, K. Wang, T. Shi, G. Cui, F. Wang, G. Xu, M. Zhu, *Adv. Funct. Mater.* **2024**, *34*, 2407262.

[19] J. Cao, X. Rao, S. Qian, D. Zhang, Y. Jin, X. Yang, J. Lu, *Adv. Energy Mater.* **2025**, *15*, e03368.

[20] L. Yang, Y.-J. Zhu, H.-P. Yu, Z.-Y. Wang, L. Cheng, D.-D. Li, J. Tao, G. He, H. Li, *Adv. Energy Mater.* **2024**, *14*, 2401858.

[21] C. Xu, B. Li, H. Du, F. Kang, *Angew. Chem., Int. Ed.* **2012**, *51*, 933.

[22] M. H. Alfaruqi, V. Mathew, J. Gim, S. Kim, J. Song, J. P. Baboo, S. H. Choi, J. Kim, *Chem. Mater.* **2015**, *27*, 3609.

[23] V. Soundharrajan, B. Sambandam, S. Kim, S. Islam, J. Jo, S. Kim, V. Mathew, Y. K. Sun, J. Kim, *Energy Storage Mater.* **2020**, 28, 407.

[24] L. Zhang, L. Chen, X. Zhou, Z. Liu, *Adv. Energy Mater.* **2015**, *5*, 1400930.

[25] N. Zhang, F. Cheng, Y. Liu, Q. Zhao, K. Lei, C. Chen, X. Liu, J. Chen, *J. Am. Chem. Soc.* **2016**, *138*, 12894.

[26] Q. Wang, S. Wang, X. Guo, L. Ruan, N. Wei, Y. Ma, J. Li, M. Wang, W. Li, W. Zeng, *Adv. Electron. Mater.* **2019**, *5*, 1900537.

[27] Y. Liu, Z. Dai, W. Zhang, Y. Jiang, J. Peng, D. Wu, B. Chen, W. Wei, X. Chen, Z. Liu, Z. Wang, F. Han, D. Ding, L. Wang, L. Li, Y. Yang, Y. Huang, *ACS Nano* **2021**, *15*, 9065.

[28] B. Tang, G. Fang, J. Zhou, L. Wang, Y. Lei, C. Wang, T. Lin, Y. Tang, S. Liang, *Nano Energy* **2018**, *51*, 579.

[29] X. Dai, F. Wan, L. Zhang, H. Cao, Z. Niu, *Energy Storage Mater.* **2019**, *17*, 143.

[30] P. He, M. Yan, G. Zhang, R. Sun, L. Chen, Q. An, L. Mai, *Adv. Energy Mater.* **2017**, *7*, 1601920.
